# Novel 5′-Norcarbocyclic Derivatives of Bicyclic Pyrrolo- and Furano[2,3-d]Pyrimidine Nucleosides

**DOI:** 10.3390/molecules23102654

**Published:** 2018-10-16

**Authors:** Anna A. Klimenko, Elena S. Matyugina, Evgeniya B. Logashenko, Pavel N. Solyev, Marina A. Zenkova, Sergey N. Kochetkov, Anastasia L. Khandazhinskaya

**Affiliations:** 1 Engelhardt Institute of Molecular Biology, Russian Academy of Sciences, 32 Vavilov St., Moscow 119991, Russia; kanna76v@gmail.com (A.A.K.); matyugina@gmail.com (E.S.M.); solyev@gmail.com (P.N.S.); kochet@eimb.ru (S.N.K.); 2 Institute of Chemical Biology and Fundamental Medicine, Siberian Branch of Russian Academy of Sciences, 8 Lavrentiev Ave., Novosibirsk 630090, Russia; evg_log@ngs.ru (E.B.L.); marzen@niboch.nsc.ru (M.A.Z.)

**Keywords:** 5′-norcarbocyclic nucleoside analogues, antiproliferative properties, structure–activity relationship

## Abstract

Here we report the synthesis and biological activity of new 5′-norcarbocyclic derivatives of bicyclic pyrrolo- and furano[2,3-d]pyrimidines with different substituents in the heterocyclic ring. Lead compound **3i**, containing 6-pentylphenyl substituent, displays inhibitory activity with respect to a number of tumor cells with a moderate selectivity index value. Compound **3i** induces cell death by the apoptosis pathway with the dissipation of mitochondrial potential.

## 1. Introduction

Nucleic acid components are involved in many vitally important metabolic processes (DNA and RNA synthesis, cell signaling, enzyme regulation and metabolism); this is why their synthetic analogues are convenient tools for studying and influencing these processes. Nucleoside and nucleotide analogues can interact with and inhibit essential enzymes such as human and viral polymerases (DNA-dependent DNA polymerases, RNA-dependent DNA polymerases or RNA-dependent RNA polymerases), kinases, ribonucleotide reductase, DNA methyltransferases, purine and pyrimidine nucleoside phosphorylase and thymidylate synthase [1]. As a result, nucleoside analogues have been in clinical use for almost 50 years and have become cornerstones of treatment for patients with cancer or viral infections [1]. However, the clinical use of these compounds is limited by important side-effects and primary or acquired drug resistance [2]. Thus, the development of new antiviral and anticancer agents is of crucial importance.

Bicyclic furano[2,3-d]pyrimidine nucleosides were first developed by McGuigan et al. as herpes virus family inhibitors [3]. The compounds bearing the 2′-deoxyribose residue are non-toxic and are highly effective inhibitors of the Varicella–Zoster virus [4], and analogues containing the 2′,3′-dideoxyribose or acyclic fragments suppress human cytomegalovirus [5]. The corresponding carbocyclic analogue was also synthesized by the same group but turned out to be less active [6]. It was shown that in order to display antiviral activity bicyclic furano[2,3-d]pyrimidine nucleosides have to be phosphorylated by viral deoxythymidine kinase, but the complete mechanism of their inhibitory effect has not yet been elucidated [5]. At the same time, significant anticancer activity was found for several small molecules which include a furo[2,3-d]pyrimidine scaffold due to their inhibitory effect against different protein kinases [7,8]. Recent data have shown that some pyrrolo- and furano[2,3-d]pyrimidine nucleosides are able not only to suppress the growth of various lines of tumor cells, but also to induce apoptosis [9,10,11]. The first 5′-norcarbocyclic derivatives of bicyclic furano[2,3-d]pyrimidines with various alkyl substituents at the 6-position of the heterocyclic base have shown antitumor activity against different cell lines [12]. Here we synthesized new representatives of bicyclic furano[2,3-d]pyrimidine nucleosides and novel bicyclic pyrrolo[2,3-d]pyrimidine nucleosides to obtain structure–activity relationship data for this family of compounds and to get additional information on the mechanisms of action and potential cellular targets for these bicyclic nucleosides.

## 2. Results and Discussion

### 2.1. Chemistry

**Scheme 1:**
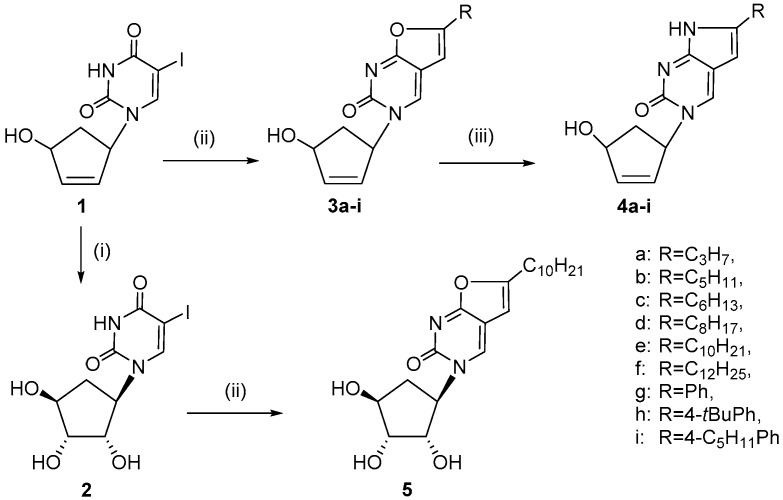
Synthesis of the compounds. Reaction conditions: (i) OsO_4_, NMMO; (ii) CuI, 10%Pd/C, RC≡CH, NEt_3_, CH_3_CN; (iii) NH_3_/MeOH.

**Figure 1:**
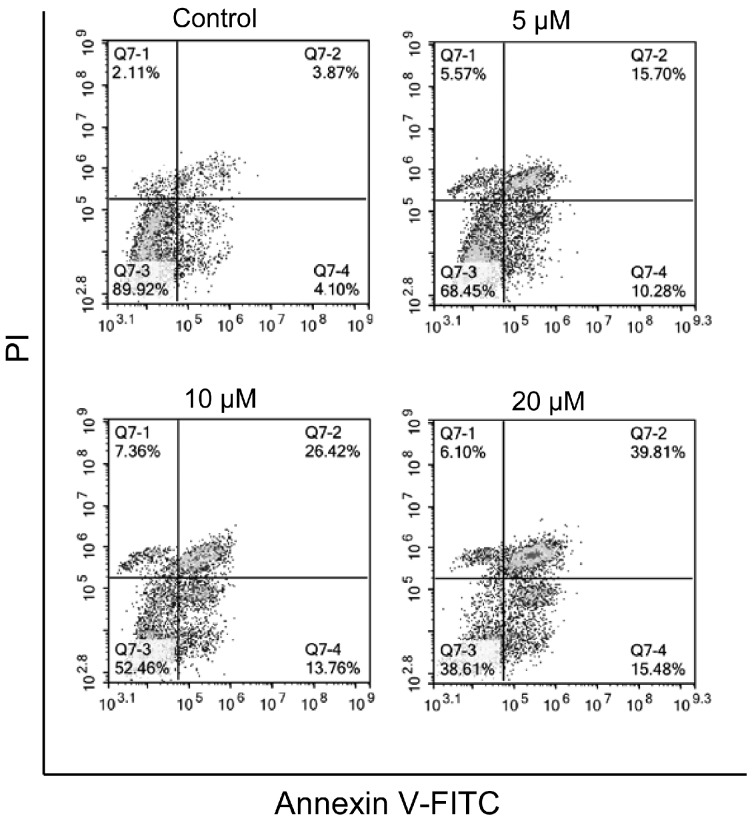
Quantification of apoptosis with annexin V binding to KB-3-1 cells. Cells were incubated in the presence of **3i** (5, 10 or 20 µM), or in the presence of DMSO (0.1% *v*/*v*) for 48 h and then Annexin V/PI staining was analyzed by flow cytometry. Etoposide was used as a standard apoptosis inducer to confirm the correct work of the system (data not shown). The results of one of three independent experiments are represented.

All the 5′-norcarbocyclic analogs of bicyclic furano- and pyrrolo[2,3d]pyrimidine nucleosides were synthesized starting from the general precursor racemic 1-(4′-hydroxy-2′-cyclopentene-1′-yl)-5-ioduracil **1** (Figure 1) which was obtained as described earlier [12,13]. 1-(2′,3′,4′-Trihydroxycyclopent-1′-yl)-5-iodouracil **2** was synthesized by oxidation of compound **1** using osmium tetroxide in the presence of *N*-methylmorpholine-*N-*oxide (NMMO) [14]. This procedure allows the *cis*-2′,3′-diol to be obtained selectively [15,16]. To prepare furano[2,3d]pyrimidine nucleosides we used Cu/Pd-catalyzed cyclisation of **1** (for **3a**–**i**) or its oxidized derivative **2** (for **5**) with corresponding alkynes in refluxing CH_3_CN. This afforded target furano[2,3d]pyrimidine nucleosides in good yields (36–82%). Such a deviation in yields was due both to the difference in alkyne structures and to the fact that isolation and purification of some products turned to be laborious. Subsequent treatment of compounds **3a**–**i** with 32% ammonia in methanol resulted in corresponding pyrrolo[2,3-d]pyrimidine analogs **4a**–**i** (Scheme 1). The reactions at 40 °C were rather slow, but such mild conditions gave us an opportunity to obtain products **4a**–**i** with good yields (57–89%) without using a bomb. It is worth remarking that preparative liquid chromatography on silica gel plates turned to be more effective for the isolation of pyrrolo[2,3-d]pyrimidine analogues **4a**–**i** than the column chromatography on silica gel, which was our choice in the case of furano[2,3-d]pyrimidine derivatives **3a**–**i** and **5**. All the compounds were synthesized as racemic mixtures.

As a result, a set of novel bicyclic furano[2,3-d]pyrimidine nucleosides **3a**–**i**, early unknown bicyclic pyrrolo[2,3-d]pyrimidine nucleosides **4a**–**i** and a new 1-(2′,3′,4′-trihydroxycyclopent1′-yl)6-decyl-3*H*-furano[2,3-d]pyrimidine-2-one **5** were obtained. The last one was synthesized as a first representative of trihydroxycyclopentyl derivatives of furano[2,3-d]pyrimidine-2-one in order to estimate the potential of this modification for antitumor activity and to gain a better structure activity relationship (SAR) understanding.

### 2.2. Biological Evaluation

#### 2.2.1. Cell Viability Assay

The target compounds were tested on different lines of tumor cells, HuTu-80 (human duodenal cancer), B16 (mouse melanoma), A549 (human lung adenocarcinoma), KB-3-1 (human squamous cell carcinoma), HeLa (human squamous cell carcinoma of the cervix), as well as on human noncancer cells hFF3.

Compounds **3b** and **4a** had no toxic effect on either normal untransfected hFF3 cells or on tumor cells in concentrations up to 100 μM. Compounds **3d**–**f**,**h** and **4f**,**i** were almost equally toxic for cancer and noncancer cells (Table 1). The pyrrole-containing compounds **4e** and **4h** were less toxic than the corresponding furan analogues **3e** and **3h**, but also reduced the viability of all tested cell lines with IC_50_ in the range from 11 μM (HeLa) to 63 μM (hFF3) for **4e** and from 15 μM (KB-3-1) to 70 μM (hFF3) for **4h**. First 2′,3′-dihydroxy derivative **5**, proved to be less toxic than the corresponding 2′,3′-didehydro-2′,3′-dideoxy analogue **3e**. Compounds **3a**, **3c**, **3g**, **3i**, **4b**–**d**, **4g** and **5** inhibited the growth of some tested tumor cells, mainly KB-3-1 and HeLa (Table 1), while they were not toxic for normal cells in concentrations up to 100 μM. Melanoma cells B16 were the most resistant towards action of these new nucleoside analogues. Only compounds **3f**, **3i**, **4e**, **4h** and **4i** had the selective toxic effect on this line with IC_50_ 4.5, 21, 25, 35 and 13.4 μM, respectively.

The nucleoside analogue **3i** was among most active compounds (Table 1) and had the most selective antiproliferative antitumor effect, especially against the HuTu80, KB-3-1 and HeLa cell lines (Table 2). Therefore, we used it as a lead compound to study the mechanism of induced cells death.

#### 2.2.2. Induction of Apoptosis

To examine whether the tested 5′-norcarbocyclic derivatives induce cell death via apoptosis Annexin V and propidium iodide analysis were used (Figure 1). KB-3-1 cells were exposed to **3i,** the most active among tested compounds, for 48 h and then flow cytometric analysis was undertaken. Annexin V binds phosphatidylserine residues, which are asymmetrically distributed toward the inner plasma membrane, and migrate to the outer plasma membrane during apoptosis [17]. The data shows that **3i** induces apoptotic cell death in 26% of KB-3-1 cells at concentrations of 5 µM. The increasing of **3i** concentration to 20 µM resulted in 55.3% apoptotic cells after 48 h of incubation of KB-3-1 cells with the analogue. Hence, the 5′-norcarbocyclic derivative **3i** induced dose-dependent apoptotic cell death.

**Figure 2:**
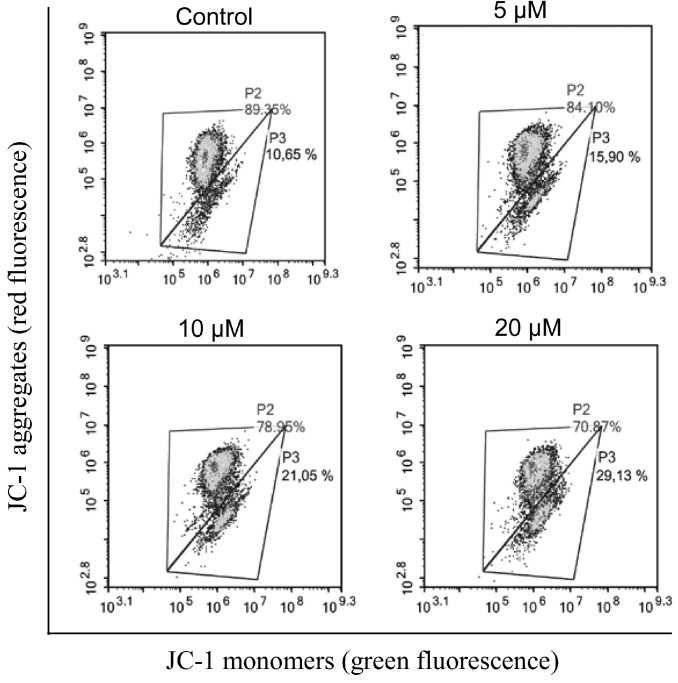
Analysis of mitochondrial transmembrane potential of KB-3-1 cells treated with the compound **3i** by flow cytometry after JC-1 staining. Cells were incubated with **3i** (5, 10 or 20 µM) or DMSO (0.1% *v*/*v*) for 48 h. In normal cells, the dye is aggregated in mitochondria, and fluoresces red. In cells with altered mitochondrial potential, the dye fails to accumulate in the mitochondria, remained as monomers in the cytoplasm, and fluoresces green.

We next investigated whether **3i** utilizes the mitochondrial ‘intrinsic’ pathway in the apoptotic death of KB-3-1 cells, since the pivotal role of mitochondria in triggering apoptosis is well established. We evaluated the mitochondrial transmembrane potential (∆*Ψ*_m_) in KB-3-1 cells exposed to **3i** using cytofluorometric analysis. Cells were stained with the specific mitochondrial cationic dye JC-1 (5,5′,6,6′-tetrachloro-1,1′,3,3′-tetraethyl benzimidazole carbocyanine iodide) that accumulates in the transmembrane space of polarized mitochondria and forms the so–called «J–aggregates», emitting red fluorescence. A decrease in ∆*Ψ*_m_ results in disappearance of J–aggregates and formation of JC-1 monomers, which emit in a green fluorescence. The cytometric analysis of KB-3-1 cells stained with JC-1 is shown in Figure 2.

In the control cells incubated in the presence of 0.1% DMSO the majority of cells shows a high emission of fluorescence in both channels due to the equilibrium between J-aggregates and monomers (Figure 2). The exposure of KB-3-1 cells to 20 µM of compound **3i** leads to a decrease of the red fluorescence value as compared to the control (0.1% DMSO).

## 3. Materials and Methods

### 3.1. Chemistry

*N*-Methylmorpholine-*N*-oxide (NMMO), peracetic acid, Pd(PPh_3_)_4_, CuI, 10% Pd/C, triethylamine, 3-(4,5-dimethylthiazol-2-yl)-2,5-diphenyltetrazolium bromide, propidium iodide and organic solvents were obtained from “Acros” (Belgium) or “Aldrich” (USA) and were used without further purification. 1-(4′-Hydroxy-2′-cyclopenten-1′-yl)-5-iodouracil (**1**) was synthesized according an earlier published protocol [12]; 1-(4′-hydroxy-2′-cyclopenten-1′-yl)-6-octyl-3Hfurano[2,3-d]-pyrimidine-2-one (**3d**), 1-(4′-hydroxy-2′-cyclopenten-1′-yl)-6-decyl-3Hfurano[2,3-d]-pyrimidine-2-one (**3e**) and 1-(4′-hydroxy-2′-cyclopenten-1′-yl)-6-dodecyl-3Hfurano[2,3-d]-pyrimidine-2-one (**3f**) were prepared as described earlier [12,13]. Annexin-FITC apoptosis staining/detection kit was from “Abcam” (Eugene, CA USA); 5,5′,6,6′-tetrachloro-1,1′,3,3′tetraethylbenzimidazolylcarbocyanine iodide (JC-1) was from “Invitrogen” (San Diego, CA, USA).

Column chromatography was performed on Silica Gel 60 0.040–0.063 mm (Merck, Germany), and systems for elution are indicated in the text. Thin layer chromatography (TLC) was performed on TLC Silica gel 60 F_254_ plates (Merck, Germany) in chloroform–methanol, 9:1 (A), or chloroform–methanol, 4:1 (B) systems. Preparative layer chromatography (PLC) was performed on PLC Silica gel 60 F_254_ plates (Merck, Germany), systems for elution are indicated in the text.

^1^H and ^13^C nuclear magnetic resonance (NMR) spectra were registered on a Bruker Avance 400 spectrometer (Bruker, Newark, Germany) using tetramethylsilane (TMS) in CDCl_3_, CD_3_OD, CDCl_3_/CD_3_OD mixture, or DMSO-*d*_6_ as internal standard. Chemical shifts are given in ppm, and the letter “*J*” indicates normal ^3^*J*_HH_ couplings and all *J* values are given in Hz.

High-resolution mass spectra (HRMS) were registered on a Bruker Daltonics micrOTOF-Q II instrument using electrospray ionization (ESI). The measurements were acquired in a negative ion mode with the following parameters: interface capillary voltage—3700 V; mass range from *m*/*z* 50 to 3000; external calibration (Electrospray Calibrant Solution, Fluka); nebulizer pressure—0.3 Bar; flow rate—3 µL/min; dry gas nitrogen (4.0 L/min); interface temperature was set at 180 or 190 °C. A syringe injection was used.

The absorbance (MTT assay) was measured on a plate reader Multiscan RC (Thermo LabSystems, Vantaa, Finland) at 570 nm. Mitochondrial transmembrane potential and the amount of apoptotic cells in samples were analyzed by flow cytometer «FC500» (Beckman Coulter, Indianapolis, IN, USA).

#### 3.1.1. General Method for the Synthesis of 1-(4′-hydroxy-2′-cyclopenten-1′-yl)-6-alkyl-3*H*-furano[2,3-d]pyrimidine-2-ones and 1-(4′-hydroxy-2′-cyclopenten-1′-yl)-6-aryl-3*H*-furano[2,3-d]-pyrimidine-2-ones (**3a**–**i**)

To the solution of 1-(4′-hydroxy-2′-cyclopenten-1′-yl)-5-iodouracil (100 mg, 0.31 mmol) in acethonitrile (5 mL) CuI (19 mg, 0.1 mmol), 10% Pd/C (15 mg) and appropriate 1-alkyne (0.38 mmol) were added and the reaction mixture was refluxed for 4 h. The progress of the reaction was monitored by TLC. Reaction mixtures were evaporated to dryness in vacuo, the residues were dissolved in the appropriate solvent mixture and **3a**–**i** were isolated and purified using column chromatography on silica gel.

*1-(4′-Hydroxy-2′-cyclopenten-1′-yl)-6-propyl-3H-furano[2,3-d]-pyrimidine-2-one* (**3a**) was purified on a silica gel column using chloroform: methanol (97:3) as an eluent with 41% yield. R*_f_* 0.35 (system A). ^1^H-NMR (CD_3_OD): 8.29 (1H, s, H4), 6.39 (1H, m, H5), 6.34-6.31 (1H, m, H2′), 6.00–5.97 (1H, m, H3′), 5.77–5.75 (1H, m, H1′), 4.87–4.82 (1H, m, H4′), 3.07–3.02 (1H, m, H5′a), 2.71–2.66 (2H, m, CH_2_CH_2_CH_3_), 1.81–1.69 (2H, m, CH_2_CH_2_CH_3_), 1.59–1.52 (1H, m, H5′b), 1.03 (3H, t, *J* = 8 Hz, CH_2_CH_2_CH_3_). ^13^C-NMR (CD_3_OD): 172.4, 160.9, 157.2, 141.1, 139.2, 131.8, 109.7, 100.2, 75.1, 63.2, 41.9, 30.5, 20.9, 13.4. HRMS (ESI, *m*/*z*) of C_14_H_16_N_2_O_3_: calcd. for [M + Na]^+^ 283.1053, found 283.1061, see Supplementary Materials.

*1-(4′-Hydroxy-2′-cyclopenten-1′-yl)-6-pentyl-3H-furano[2,3-d]-pyrimidine-2-one* (**3b**) was purified on silica gel column using chloroform: methanol (98:2 to 95:5) as an eluent with 69% yield. R*_f_* 0.36 (system A). ^1^H-NMR (CD_3_OD): 8.29 (1H, s, H4), 6.38 (1H, m, H5), 6.35–6.31 (1H, m, H2′), 6.00–5.97 (1H, m, H3′), 5.81–5.75 (1H, m, H1′), 4.87–4.82 (1H, m, H4′), 3.05–3.00 (1H, m, H5′a), 2.71 (2H, t, *J* = 8 Hz, CH_2_CH_2_(CH_2_)_2_CH_3_), 1.75–1.70 (2H, m, CH_2_CH_2_(CH_2_)_2_CH_3_), 1.59–1.52 (1H, m, H5′b), 1.43–1.37 (4H, m, CH_2_CH_2_(CH_2_)_2_CH_3_), 0.94 (3H, t, *J* = 8 Hz, CH_2_CH_2_(CH_2_)_2_CH_3_). ^13^C-NMR (CD_3_OD): 171.4, 160.1, 156.3, 140.1, 138.2, 130.8, 108.7, 99.1, 74.1, 62.2, 40.9, 30.9, 27.5, 26.3, 21.9, 12.8. HRMS (ESI, *m*/*z*) of C_16_H_20_N_2_O_3_: calcd. for [M + H]^+^ 289.1547, found 289.1545.

*1-(4′-Hydroxy-2′-cyclopenten-1′-yl)-6-hexyl-3H-furano[2,3-d]-pyrimidine-2-one* (**3c**) was purified using two-step chromatography on silicа gel. The first column was eluted with chloroform: methanol (98:2 to 95:5) and the second one with hexane: ethyl acetate (1:4) to ethyl acetate: methanol (97:3) as an eluent to give (**3c**) with 52% yield. R*_f_* 0.36 (system A). ^1^H-NMR (CD_3_OD): 8.27 (1H, s, H4), 6.36 (1H, s, H5), 6.32–6.30 (1H, m, H2′), 5.98–5.96 (1H, m, H3′), 5.76–5.75 (1H, m, H1′), 4.85–4.83 (1H, m, H4′), 3.05–3.01 (1H, m, H5′a), 2.69 (2H, t, *J* = 8Hz, CH_2_CH_2_(CH_2_)_3_CH_3_), 1.76–1.68 (2H, m, CH_2_CH_2_(CH_2_)_3_CH_3_), 1.56–1.51 (1H, m, H5′b), 1.35–1.29 (6H, m, CH_2_CH_2_(CH_2_)_3_CH_3_), 0.92 (3H, t, *J* = 8Hz, CH_2_CH_2_(CH_2_)_3_CH_3_). ^13^C-NMR (CD_3_OD): 172.8, 161.5, 157.7, 141.5, 139.6, 132.2, 110.1, 100.5, 75.5, 63.6, 42.3, 32.6, 29.7, 29.0, 28.0, 23.6, 14.3. HRMS (ESI, *m*/*z*) of C_17_H_22_N_2_O_3_: calcd. for [M + H]^+^ 303.1703, found 303.1698.

*1-(4′-Hydroxy-2′-cyclopenten-1′-yl)-6-phenyl-3H-furano[2,3-d]-pyrimidine-2-one* (**3g**) was purified using column chromatography with chloroform: methanol (97:3) as an eluent and then on a PLC in ethyl acetate with 37% yield. R*_f_* 0.34 (system A). ^1^H-NMR (CDCl_3_-CD_3_OD): 8.17 (1H, s, H4), 7.71–7.69 (2H, m, Ph), 7.41–7.32 (3H, m, Ph), 6.69 (1H, s, H5), 6.27–6.26 (1H, m, H2′), 5.87–5.86 (1H, m, H3′), 5.81–5.80 (1H, m, H1′), 4.85–4.83 (1H, m, H4′), 3.01–2.94 (1H, m, H5′a), 1.60–1.56 (1H, m, H5′b). ^13^C-NMR (CDCl_3_-CD_3_OD): 171.3, 156.0, 140.2, 138.4, 131.7, 129.80, 129.0 × 2, 128.3, 125.0 × 2, 109.1, 97.7, 74.4, 62.4, 41.1, 29.7. HRMS (ESI, *m*/*z*) of C_17_H_14_N_2_O_3_: calcd. for [M + H]^+^ 295.1077, found 295.1077.

*1-(4′-Hydroxy-2′-cyclopenten-1′-yl)-6-tertbutylphenyl-3H-furano[2,3-d]-pyrimidine-2-one* (**3h**) was purified on a silica gel column using chloroform: methanol (98:2) as an eluent with 36% yield. R*_f_* 0.36 (system A). ^1^H-NMR (CDCl_3_-CD_3_OD): 8.14 (1H, s, H4), 7.63 (2H, d, *J* = 8 Hz, Ph), 7.41 (2H, d, *J* = 8 Hz, Ph), 6.63 (1H, s, H5), 6.26 (1H, m, H2′), 5.86 (1H, m, H3′), 5.80 (1H, m, H1′), 4.84 (1H, m, H4′), 3.01–2.93 (1H, m, H5′a), 1.61–1.57 (1H, m, H5′b), 1.28 (9H, s, *t*Bu). ^13^C-NMR (CDCl_3_-CD_3_OD): 171.3, 156.2, 155.9, 153.4, 140.1, 137.9, 131.7, 125.9 × 2, 125.5, 124.8 × 2, 109.2, 96.9, 74.4, 62.3, 41.2, 34.9, 31.1 × 3. HRMS (ESI, *m*/*z*) of C_21_H_22_N_2_O_3_: calcd. for [M + H]^+^ 351.1703, found 351.1698.

*1-(4′-Hydroxy-2′-cyclopenten-1′-yl)-6-(4-pentylphenyl)-3H-furano[2,3-d]-pyrimidine-2-one* (**3i**) was purified using two-step chromatography on silicа gel. The first column was eluted with chloroform: methanol (98:2 to 97:3) and the second one with hexane: ethyl acetate (1:4) to ethyl acetate: methanol (97:3) as an eluent to give (**3i**) with 82% yield. R*_f_* 0.36 (system A). ^1^H-NMR (CDCl_3_-CD_3_OD): 8.33 (1H, s, H4), 7.68 (2H, d, *J* = 8.5 Hz, Ph), 7.27 (2H, d, *J* = 8.5 Hz, Ph), 6.84 (1H, s, H5), 6.35–6.32 (1H, m, H2′), 5.95–5.93 (1H, m, H3′), 5.85–5.82 (1H, m, H1′), 4.88–4.86 (1H, m, H4′), 3.07–3.00 (1H, m, H5′a), 2.65 (2H, t, *J* = 9Hz, CH_2_CH_2_(CH_2_)_2_CH_3_), 1.66–1.57 (3H, m, H5′b and CH_2_CH_2_(CH_2_)_2_CH_3_), 1.35–1.32 (4H, m, CH_2_CH_2_(CH_2_)_2_CH_3_), 0.89 (3H, t, *J* = 9Hz, CH_2_CH_2_(CH_2_)_2_CH_3_). ^13^C-NMR (CDCl_3_-CD_3_OD): 171.1, 156.3, 156.0, 145.0, 140.0, 138.4, 130.09, 128.8 × 2, 125.5, 124.6 × 2, 109.5, 96.8, 73.9, 62.1, 41.9, 35.4, 31.1, 30.6, 22.1, 13.3. HRMS (ESI, *m*/*z*) of C_22_H_24_N_2_O_3_: calcd. for [M + H]^+^ 365.1861, found 365.1860.

*1-(2′,3′,4′-Trihydroxycyclopent1’-yl)-5-iodouracil* (**2**) was synthesized using the common procedure [14] ^1^H-NMR(DMSO-*d*_6_): 11.58 (1H, s, NH), 8.12 (1H, s, H6), 5.14 (1H, d, *J* = 4 Hz, OH), 4.95 (1H, d, *J* = 6 Hz, OH), 4.77 (1H, d, *J* = 4 Hz, OH), 4.73–4.68 (1H, m, H2′), 4.17–4.12 (1H, m, H1′), 3.82–3.79 (1H, m, H3′), 3.69–3.66 (1H, m, H4′), 2.43–2.40 (1H, m, H5′a), 1.42–1.37 (1H, m, H5′b).

*1-(2′,3′,4′-Trihydroxycyclopent-1′-yl)-6-decyl-3H-furano[2,3-d]-pyrimidine-2-one* (**5**) was synthesized as described for compounds **3a**–**i**, starting from 1-(2′,3′,4′-trihydroxycyclopenten-1′-yl)-5-iodouracil **2**. The product was purified using chloroform: methanol (4:1) as an eluent with 54% yield. R*_f_* 0.25 (system B). ^1^H-NMR(CD_3_OD): 8.44 (1H, s, H4), 6.36 (1H, s, H5), 5.05–4.97 (1H, m, H2′), 4.55–4.50 (1H, m, H1′), 4.06–4.03 (1H, m, H3′), 3.94–3.92 (1H, m, H4′), 2.76–2.71 (1H, m, H5′a), 2.69–2.64 (2H, m, CH_2_(CH_2_)_8_CH_3_), 1.73–1.66 (3H, m, H5′b + CH_2_CH_2_(CH_2_)_7_CH_3_), 1.39–1.26 (14H, m, CH_2_CH_2_(CH_2_)_7_CH_3_), 0.68 (3H, t, *J* = 8 Hz, CH_2_(CH_2_)_8_CH_3_). ^13^C-NMR(CDCl_3_-CD_3_OD): 171.35, 160.15, 156.56, 139.75, 108.87, 99.08, 76.68, 75.61, 73.77, 65.02, 35.54, 31.63, 29.25, 29.19, 29.00, 28.94, 28.66, 27.56, 26.57, 22.29, 13.01. HRMS: found *m*/*z* 393.2384, calculated for C_21_H_32_N_2_O_5_ [M + H]^+^ 393.2388.

#### 3.1.2. General Method for the Synthesis of 1-(4′-hydroxy-2′-cyclopenten-1′-yl)-6-alkyl-3*H*-pyrrolo[2,3-d]pyrimidine-2-ones and 1-(4′-hydroxy-2′-cyclopenten-1′-yl)-6-aryl-3*H*-pyrrolo[2,3-d]-pyrimidine-2-ones (**4a**–**i**).

To the corresponding 1-(4′-hydroxy-2′-cyclopenten-1′-yl)-6-alkyl-3*H*-furano[2,3-d]pyrimidine-2-one or 1-(4′-hydroxy-2′-cyclopenten-1′-yl)-6-aryl-3H-furano[2,3-d]-pyrimidine-2-one (50 mg) a solution of 32% NH_3_ in MeOH (15 mL) was added. The reaction mixture was kept at 40 °C for 48 h. Solvent then was evaporated in vacuo and a new portion of 32% NH_3_ in MeOH was added (15 mL). The procedure was repeated three times controlling the progress of the reaction by TLC.

*1-(4′-Hydroxy-2′-cyclopenten-1′-yl)-6-propyl-3H-pyrrolo[2,3-d]-pyrimidine-2-one* (**4a**) was purified using PLC with chloroform: methanol (9:1) as an eluent with 70% yield. R*_f_* 0.33 (system A). ^1^H-NMR (DMSO-*d*_6_): 11.05 (1H, s, NH), 8.03 (1H, s, H4), 6.18–6.16 (1H, m, H2′), 5.91 (1H, s, H5), 5.87–5.86 (1H, m, H3′), 5.69–5.66 (1H, m, H1′), 5.21 (1H, m, OH), 4.68 (1H, m, H4′), 2.87–2.83 (1H, m, H5′a), 2.50 (2H, t, *J* = 8 Hz, CH_2_CH_2_CH_3_), 1.64–1.59 (2H, m, CH_2_CH_2_CH_3_), 1.38–1.34 (1H, m, H5′b), 0.89 (3H, t, *J* = 8 Hz, CH_2_CH_2_CH_3_). ^13^C-NMR (DMSO-*d*_6_): 158.9, 154.4, 142.1, 139.8, 135.9, 131.4, 109.2, 96.2, 73.5, 60.4, 41.2, 29.4, 20.9, 13.4. HRMS (ESI, *m*/*z*) of C_14_H_17_N_3_O_2_: calcd. for [M + Na]^+^ 282.1213, found 282.1211.

*1-(4′-Hydroxy-2′-cyclopenten-1′-yl)-6-pentyl-3H-pyrrolo[2,3-d]-pyrimidine-2-one* (**4b**). The product was purified using PLC with chloroform: methanol (9:1) as an eluent with 57% yield. R*_f_* 0.33 (system A). ^1^H-NMR (CD_3_OD): 8.18 (1H, s, H4), 6.31–6.28 (1H, m, H2′), 6.01 (1H, m, H5), 5.99–5.96 (1H, m, H3′), 5.85–5.81 (1H, m, H1′), 4.89 (1H, m, H4′), 3.08–3.03 (1H, m, H5′a), 2.64 (2H, t, *J* = 8 Hz, CH_2_CH_2_(CH_2_)_2_CH_3_), 1.75–1.68 (2H, m, CH_2_CH_2_(CH_2_)_2_CH_3_), 1.57–1.49 (1H, m, H5′b), 1.40–1.35 (4H, m, CH_2_CH_2_(CH_2_)_2_CH_3_), 0.93 (3H, t, *J* = 8 Hz, CH_2_CH_2_(CH_2_)_2_CH_3_). ^13^C-NMR (CD_3_OD): 158.6, 156.3, 143.8, 139.5, 136.0, 131.3, 111.2, 96.4, 74.2, 61.7, 41.2, 31.1, 27.6, 27.5, 22.0, 12.9. HRMS (ESI, *m*/*z*) of C_16_H_21_N_3_O_2_: calcd. for [M + H]^+^ 288.1706, found 288.1710.

*1-(4′-Hydroxy-2′-cyclopenten-1′-yl)-6-hexyl-3H-pyrrolo[2,3-d]-pyrimidine-2-one* (**4c**). The product was purified using PLC with chloroform: methanol (9:1) as an eluent with 64% yield. R*_f_* 0.33 (system A). ^1^H-NMR (CD_3_OD): 8.16 (1H, s, H4), 6.28–6.27 (1H, m, H2′), 6.00 (1H, m, H5), 5.96–5.95 (1H, m, H3′), 5.81 (1H, m, H1′), 4.83–4.80 (1H, m, H4′), 3.08–3.00 (1H, m, H5′a), 2.62 (2H, t, *J* = 8 Hz, CH_2_CH_2_(CH_2_)_3_CH_3_), 1.70–1.66 (2H, m, CH_2_CH_2_(CH_2_)_3_CH_3_), 1.54–1.48 (1H, m, H5′b), 1.39–1.33 (6H, m, CH_2_CH_2_(CH_2_)_3_CH_3_), 0.90 (3H, t, *J* = 8 Hz,CH_2_CH_2_(CH_2_)_3_CH_3_). ^13^C-NMR (CD_3_OD): 160.0, 157.8, 145.2, 140.9, 137.4, 132.8, 112.6, 97.8, 75.6, 63.1, 42.6, 32.7, 29.9, 29.3, 29.0, 23.6, 14.3. HRMS (ESI, *m*/*z*) of C_17_H_23_N_3_O_2_: calcd. for [M + H]^+^ 302.1863, found 302.1868.

*1-(4′-Hydroxy-2′-cyclopenten-1′-yl)-6-octyl-3H-pyrrolo[2,3-d]-pyrimidine-2-one* (**4d**). The product was purified using PLC with chloroform: methanol (9:1) as an eluent with 89% yield. R*_f_* 0.33 (system A). ^1^H-NMR (CD_3_OD): 8.18 (1H, s, H4), 6.31–6.29 (1H, m, H2′), 6.02 (1H, s, H5), 5.98–5.96 (1H, m, H3′), 5.83 (1H, m, H1′), 4.85–4.82 (1H, m, H4′), 3.08–3.00 (1H, m, H5′a), 2.64 (2H, t, *J* = 8 Hz, CH_2_CH_2_(CH_2_)_5_CH_3_), 1.72–1.67 (2H, m, CH_2_CH_2_(CH_2_)_5_CH_3_), 1.56–1.48 (1H, m, H5′b), 1.35–1.27 (10H, m, CH_2_CH_2_(CH_2_)_5_CH_3_), 0.91 (3H, t, *J* = 8 Hz, CH_2_CH_2_(CH_2_)_5_CH_3_). ^13^C-NMR (DMSO-*d*_6_): 160.0, 157.8, 145.2, 140.9, 137.4, 132.8, 112.6, 97.9, 75.6, 63.1, 42.6, 33.0, 30.4, 30.3, 30.2, 29.3, 29.0, 23.7, 14.4. HRMS (ESI, *m*/*z*) of C_19_H_27_N_3_O_2_: calcd. for [M + Na]^+^ 352.2176, found 352.2177.

*1-(4′-Hydroxy-2′-cyclopenten-1′-yl)-6-decyl-3H-pyrrolo[2,3-d]-pyrimidine-2-one* (**4e**) was purified using PLC with chloroform: methanol (9:1) as an eluent with 67% yield. R*_f_* 0.33 (system A). ^1^H-NMR (CD_3_OD): 8.18 (1H, s, H4), 6.31–6.28 (1H, m, H2′), 6.02 (1H, s, H5), 5.99–5.96 (1H, m, H3′), 5.85–5.81 (1H, m, H1′), 4.85–4.82 (1H, m, H4′), 3.08–3.01 (1H, m, H5′a), 2.64 (2H, t, *J* = 8 Hz, CH_2_CH_2_(CH_2_)_7_CH_3_), 1.72–1.67 (2H, m, CH_2_CH_2_(CH_2_)_7_CH_3_), 1.57–1.49 (1H, m, H5′b), 1.35–1.30 (14H, m, CH_2_CH_2_(CH_2_)_7_CH_3_), 0.91 (3H, t, *J* = 8 Hz, CH_2_CH_2_(CH_2_)_7_CH_3_). ^13^C-NMR (DMSO-*d*_6_): 160.0, 157.7, 145.2, 140.9, 137.4, 132.8, 112.6, 97.9, 75.6, 63.1, 42.6, 33.0, 30.6, 30.6, 30.4 × 2, 30.2, 29.3, 29.0, 23.7, 14.4. HRMS (ESI, *m*/*z*) of C_21_H_31_N_3_O_2_: calcd. for [M + Na]^+^ 380.2308, found 380.2309.

*1-(4′-Hydroxy-2′-cyclopenten-1′-yl)-6-dodecyl-3H-pyrrolo[2,3-d]-pyrimidine-2-one* (**4f**) was purified using PLC with chloroform: methanol (9:1) as an eluent with 66% yield. R*_f_* 0.38 (system A). ^1^H-NMR (CDCl_3_): 8.28 (1H, s, H4), 6.37–6.35 (1H, m, H2′), 5.92 (1H, s, H5), 5.88–5.87 (1H, m, H3′), 5.80–5.77 (1H, m, H1′), 4.95–4.92 (1H, m, H4′), 3.04–2.094 (1H, m, H5′a), 2.70 (2H, t, *J* = 8 Hz, CH_2_CH_2_(CH_2_)_9_CH_3_), 1.82–1.77 (2H, m, CH_2_CH_2_(CH_2_)_9_CH_3_), 1.71–1.66 (1H, m, H5′b), 1.36–1.26 (18H, m, CH_2_CH_2_(CH_2_)_9_CH_3_), 0.89 (3H, t, *J* = 8 Hz, CH_2_CH_2_(CH_2_)_9_CH_3_). ^13^C-NMR (CDCl_3_): 156.9, 153.8, 144.6, 140.4, 138.2, 131.6, 111.2, 96.9, 74.9, 62.5, 41.3, 32.0, 29.7 × 4, 29.5, 29.4, 29.3, 28.1 × 2, 22.7, 14.2. HRMS (ESI, *m*/*z*) of C_23_H_35_N_3_O_2_: calcd. for [M + H]^+^ 386.2802, found 386.2804.

*1-(4′-Hydroxy-2′-cyclopenten-1′-yl)-6-phenyl-3H-pyrrolo[2,3-d]-pyrimidine-2-one* (**4g**) was purified using PLC with chloroform: methanol (9:1) as an eluent with 71% yield. R*_f_* 0.33 (system A). ^1^H-NMR (CD_3_OD): 8.33 (1H, s, H4), 7.75–7.73 (2H, m, Ph), 7.45–7.42 (2H, m, Ph), 7.38–7.36 (1H, m, Ph), 6.66 (1H, s, H5), 6.32–6.30 (1H, m, H2′), 6.00–5.98 (1H, m, H3′), 5.82 (1H, m, H1′), 4.86–4.82 (1H, m, H4′), 3.10–3.02 (1H, m, H5′a), 1.59–1.53 (1H, m, H5′b). ^13^C-NMR (CD_3_OD): 160.7, 157.8, 142.4, 141.1, 139.1, 132.6, 132.0, 130.1 × 2, 129.8, 126.4 × 2, 112.8, 97.9, 75.6, 63.4, 42.7. HRMS (ESI, *m*/*z*) of C_17_H_15_N_3_O_2_: calcd. for [M + H]^+^ 294.1237, found 294.1233.

*1-(4′-Hydroxy-2′-cyclopenten-1′-yl)-6-(4-tertbutylphenyl)-3H-pyrrolo[2,3-d]-pyrimidine-2-one* (**4h**) was purified using PLC with chloroform: methanol (9:1) as an eluent with 61% yield. R*_f_* 0.40 (system A). ^1^H-NMR (CDCl_3_): 8.12 (1H, s, H4), 7.58 (2H, d, *J* = 8 Hz, Ph), 7.43 (2H, d, *J* = 8 Hz, Ph), 6.39 (1H, s, H5), 6.29–6.27 (1H, m, H2′), 5.88–5.86 (1H, m, H3′), 5.82 (1H, m, H1′), 4.87 (1H, m, H4′), 3.06–3.00 (1H, m, H5′a), 1.66–1.62 (1H, m, H5′b), 1.32 (9H, s, *t*Bu). ^13^C-NMR (CDCl_3_): 159.0, 155.8, 152.2, 140.9, 139.7, 137.2, 131.9, 127.5, 126.0 × 2, 124.9 × 2, 111.5, 95.6, 74.6, 62.3, 41.3, 34.7, 31.1 × 3. HRMS (ESI, *m*/*z*) of C_21_H_23_N_3_O_2_: calcd. for [M + H]^+^ 350.1863, found 350.1864.

*1-(4′-Hydroxy-2′-cyclopenten-1′-yl)-6-(4-pentylphenyl)-3H-pyrrolo[2,3-d]-pyrimidine-2-one* (**4i**). The product was purified using two-step chromatography. The first column was eluted with chloroform: methanol (98:2 to 97:3) and the second one with hexane: ethyl acetate (1:4) to ethyl acetate: methanol (97:3) as an eluent to give (**4i**) with 72% yield. R*_f_* 0.36 (system A). ^1^H-NMR (CDCl_3_-CD_3_OD): 8.27 (1H, s, H4), 7.62 (2H, d, *J* = 8.5 Hz, Ph), 7.23 (2H, d, *J* = 8.5 Hz, Ph), 6.58 (1H, s, H5), 6.29–6.27 (1H, m, H2′), 5.97–5.95 (1H, m, H3′), 5.81–5.79 (1H, m, H1′), 4.83–4.80 (1H, m, H4′), 3.09–2.97 (1H, m, H5′a), 2.62 (2H, t, *J* = 9 Hz, CH_2_CH_2_(CH_2_)_2_CH_3_), 1.64–1.55 (3H, m, H5′b and CH_2_CH_2_(CH_2_)_2_CH_3_), 1.34–1.29 (4H, m, CH_2_CH_2_(CH_2_)_2_CH_3_), 0.88 (3H, t, *J* = 9 Hz, CH_2_CH_2_(CH_2_)_2_CH_3_). ^13^C-NMR (CDCl_3_-CD_3_OD): 159.2, 156.4, 143.8, 141.2, 139.67, 137.2, 131.3, 128.7 × 2, 125.7, 125.0 × 2, 111.6, 95.8, 78.0, 74.2, 61.9, 41.3, 35.2, 31.2, 30.8, 22.2, 13.0. HRMS (ESI, *m*/*z*) of C_22_H_25_N_3_O_2_: calcd. for [M + H]^+^ 364.2020, found 364.2020.

### 3.2. Biological Assay

#### 3.2.1. Cell Cultures

Human KB-3-1 epidermoid carcinoma cell line, human HeLa cervical epithelioid carcinoma cell line, human HuTu-80 duodenal cancer cells, human A549 lung carcinoma epithelial cells, mouse B16 melanoma cell line and human hFF3 fibroblast cells were obtained from the Russian Cell Culture Collection (St. Petersburg, Russia) and were cultured in DMEM (hFF3 cells in IMDM) supplemented with 10% (*v*/*v*) heat-inactivated fetal bovine serum, penicillin (100 U mL^−1^), streptomycin (100 µg mL^−1^) and amphotericin (250 µg mL^−1^). Cells were maintained in a humidified atmosphere of 5% CO_2_ at 37 °C.

All compounds were dissolved in dimethylsulfoxide (DMSO) and stock solutions (10 mmol L^−1^) were stored at −20 °C.

After treatments, both floating and adherent scrapped cells were collected by centrifugation and used for further analysis.

#### 3.2.2. Cell Viability Analysis by MTT Assay

Cells growing in the logarithmic phase were seeded in triplicate in 96-well plates at a density of 5 × 10^3^ cells per well for HeLa and HuTu-80 cells, 7 × 10^3^ cells per well for KB-3-1 and hFF3, 10 × 10^3^ for A549 and 20 × 10^3^ for B16. The plates were incubated at 37 °C in a humidified 5% CO_2_ atmosphere. Cells were allowed to adhere to the surface for 24 h and then tested compounds were added at different concentrations and incubation was continued for 48 h. Then [3-(4,5-dimethylthiazol-2-yl)-2,5-diphenyltetrazolium bromide] (MTT) solution (10 µL, 5 mg mL^−1^) was added to each well and the incubation was continued for an additional 3 h. The dark blue formazan crystals formed within the healthy cells were solubilized with DMSO and the absorbance was measured using a Multiscan RC plate reader at 570 nm. The IC_50_ was determined as the compound concentration required to decrease the A_570_ to 50% compared to the control (no tested compounds, DMSO) and was determined by interpolation from dose-response curves.

#### 3.2.3. Apoptosis Detection by Annexin V Staining

Exponentially growing KB-3-1 cells in 6–well plates (5 × 10^5^ cells per well) were treated with **3i** (5, 10 and 20 µM) or with 0.1% (*v*/*v*) DMSO as a control for 48 h. The cells were stained with Annexin V-FITC and propidium iodide by the Annexin-FITC apoptosis staining/detection kit (Abcam) according to the instruction of the manufacturer. Briefly, cells were collected by scrapping, washed twice with cold PBS, and centrifuged (400 g, 5 min). Cells were resuspended in binding buffer (500 µL) and Annexin V–FITC (5 µL) and PI (5 µL) were added. Cells were incubated for 5 min at 20 °C in the dark. Finally, binding buffer (300 µL) was added to each tube, and the amount of apoptotic cells in samples were analyzed by flow cytometry. For each sample, 10,000 ungated events were acquired. Annexin V + PI − cells represent the early apoptotic populations. Annexin V + PI + cells represent either late apoptotic or secondary necrotic populations.

#### 3.2.4. Mitochondria Depolarization Analysis

Mitochondria involvement in apoptosis was measured by mitochondrial depolarization occurring early during onset of apoptosis. KB-3-1 cells were incubated with **3i** (5, 10 and 20 µM) or 0.1% (*v*/*v*) DMSO as a control for 48 h. Then, cells were collected and incubated in complete media in the dark with mitochondrial potential sensor JC-1 (5 µg mL^−1^) at 37 °C for 30 min, washed with cold PBS and resuspended in PBS (400 µL). Fluorescences of J–aggregate and J–monomer were recorded in the fluorescence channels 2 (FL2) and 1 (FL1), respectively, with flow cytometer «FC500». Necrotic fragments were electronically gated out, on the basis of morphological characteristics on the forward light scatter versus side light scatter dot plot.

## 4. Conclusions

The comparative evaluation of the effects of synthesized nucleoside analogues on the growth and viability of tumor cell cultures from various origins and on normal cells has revealed that cytotoxicity depends on both the type of bicyclic system (pyrrolo- or furano[2,3-d]pyrimidine) and the structure of a substituent in the 6th position of the heterocyclic base. Furano[2,3-d]pyrimidine **3i**, bearing pentylphenyl substituent, is the most promising among synthesized 5′-norcarbocyclic derivatives of 6-substituted bicyclic pyrrolo- and furano[2,3-d]pyrimidines. This demonstrated inhibitory activities with respect to tumor cells with the selectivity index value about 15–20 depending on the nature and origin of tumor cells. In an attempt to understand the mechanism of the action, we showed that **3i** induces cell death by apoptosis pathway with the dissipation of mitochondrial potential.

## Figures and Tables

**Table 1:**
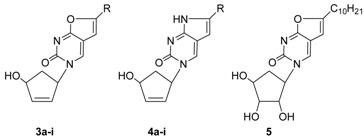
Antiproliferative activity of the compounds, IC_50_ (µM).

Compound	R	HuTu80	B16	A549	KB-3-1	HeLa	hHFF3
**3a**	C_3_H_7_	>100	>100	>100	40.1 ± 5.2	63.4 ± 6.8	>100
**4a**		>100	>100	>100	>100	>100	>100
**3b**	C_5_H_11_	>100	>100	>100	>100	>100	>100
**4b**		80.4 ± 0.9	>100	45.3 ± 3.9	25.2 ± 3.4	50.1 ± 4.7	>100
**3c**	C_6_H_13_	100	>100	50.3 ± 6.1	45.3 ± 5.1	>100	>100
**4c**		20.4 ± 3.9	>100	35.2 ± 4.1	25.6 ± 2.9	50.7 ± 6.2	>100
**3d**	C_8_H_17_	46.3 ± 5.7	46.2 ± 3.4	54.1 ± 6.1	47.3 ± 4.2	40.6 ± 3.9	48.2 ± 5.1
**4d**		30.2 ± 2.9	>100	100	25.4 ± 3.1	20.1 ± 1.9	>100
**3e**	C_10_H_21_	7.3 ± 2.4	21.3 ± 3.1	36.2 ± 11.3	11.2 ± 5.3	4.5 ± 0.9	11.6 ± 2.1
**4e**		23.4 ± 7.8	25.1 ± 2.4	36.2 ± 6.2	18.4 ± 7.1	11 ± 2.1	62.5 ± 8.3
**3f**	C_12_H_25_	7.1 ± 0.8	4.5 ± 0.3	10.5 ± 1.9	10.2 ± 4.1	2.5 ± 0.3	10.2 ± 1.8
**4f**		3.1 ± 0.4	3.1 ± 0.3	4.5 ± 0.2	4.5 ± 0.9	3.2 ± 0.4	3.4 ± 0.7
**3g**	Ph	>100	>100	>100	30.1 ± 4.1	45.3 ± 5.1	>100
**4g**		>100	>100	50.2 ± 6.7	45.4 ± 5.3	90.2 ± 9.6	>100
**3h**	4-*t*BuPh	9.9 ± 1.7	10.1 ± 2.3	7.2 ± 0.8	1.7 ± 0.2	2.3 ± 0.3	5.1 ± 0.7
**4h**		25.4 ± 3.1	35.6 ± 4.2	25.3 ± 2.9	15.3 ± 1.1	25.8 ± 4.6	70.9 ± 9.1
**3i**	4-C_5_H_11_Ph	5.1 ± 0.6	21.3 ± 2.9	47.5 ± 5.8	8.2 ± 1.3	6.5 ± 0.9	>100
**4i**		8.5 ± 1.1	13.4 ± 3.2	15.6 ± 4.1	9.8 ± 0.7	11.1 ± 0.9	21.9 ± 4.8
**5**	C_10_H_21_	>100	>100	>100	50.3 ± 9.8	70.2 ± 8.9	>100

**Table 2:** Selectivity index of the tested compounds.

Compound	R	HuTu80	B16	A549	KB-3-1	HeLa
**3a**	C_3_H_7_	1	1	1	>2.5	>1.6
**4a**		1	1	1	1	1
**3b**	C_5_H_11_	1	1	1	1	1
**4b**		>1.2	1	>2.2	>3.9	>1.9
**3c**	C_6_H_13_	1	1	>1.9	>2.2	1
**4c**		>4.9	1	>2.8	>3.9	>1.9
**3d**	C_8_H_17_	1	1	0.9	1	1.2
**4d**		>3.3	1	1	>3.9	>4.9
**3e**	C_10_H_21_	1.6	0.5	0.3	1	2.6
**4e**		2.6	2.5	1.7	3.4	5.7
**3f**	C_12_H_25_	1.4	2.3	1	1	4.1
**4f**		1.1	1.1	0.8	0.8	1
**3g**	Ph	1	1	1	>3.3	>2.2
**4g**		1	1	>2	>2.2	>1.1
**3h**	4-*t*BuPh	0.5	0.5	0.7	3	2.2
**4h**		2.8	1.9	2.8	4.6	2.7
**3i**	4-C_5_H_11_Ph	**>19.6**	>4.7	>2.1	**>12.2**	**>15.3**
**4i**		2.6	1.6	1.4	2.2	1.9
**5**	C_10_H_21_	1	1	1	>2	>1.4

The selectivity index (SI) was the ratio of IC_hFF3_ (cytotoxicity on normal hFF3 cells) to IC_50_ of cancer cells.

## Supplementary Materials

Copies of the NMR spectra are available online.

## Abbreviations

MTT	3-(4,5-Dimethylthiazol-2-yl)-2,5-diphenyltetrazolium bromide
NMMO	*N*-methylmorpholine *N*-oxide
JC-1	5,5′,6,6′-tetrachloro-1,1′,3,3′-tetraethylbenzimidazolylcarbocyanine iodide
PI	propidium iodide
PLC	preparative layer chromatography
IC50	the compound concentration that results in 50% cell survival as measured by the MTT assay

## References

[1] Jordheim L.P., Durantel D., Zoulim F., Dumontet C. (2013). Advances in the development of nucleoside and nucleotide analogues for cancer and viraldiseases. Nat. Rev. Drug Discov..

[2] Galmarini C., Popowycz F., Joseph B. (2008). Cytotoxic Nucleoside Analogues: Different Strategies to Improve their Clinical Efficacy. Curr. Med. Chem..

[3] McGuigan C., Yarnold C.J., Jones G., Velázquez S., Barucki H., Brancale A., Andrei G., Snoeck R., De Clercq E., Balzarini J. (1999). Potent and selective inhibition of varicella-zoster virus (VZV) by nucleoside analogues with an unusual bicyclic base. J. Med. Chem..

[4] Balzarini J., McGuigan C. (2002). Bicyclic pyrimidine nucleoside analogues (BCNAs) as highly selective and potent inhibitors of varicella-zoster virus replication. J. Antimicrob. Chemother..

[5] Jahnz-Wechmann Z., Framski G., Januszczyk P., Boryski J. (2015). Bioactive fused heterocycles: Nucleoside analogues with an additional ring. Eur. J. Med. Chem..

[6] Migliore M.D., Zonta N., McGuigan C., Henson G., Andrei G., Snoeck R., Balzarini J. (2007). Synthesis and Antiviral Activity of the Carbocyclic Analogue of the Highly Potent and Selective Anti-VZV Bicyclo Furano Pyrimidines. J. Med. Chem..

[7] Dincer S., Cetin K.T., Onay-Besikci A., Olgen S. (2013). Synthesis, biological evaluation and docking studies of new pyrrolo[2,3-d] pyrimidine derivatives as Src family-selective tyrosine kinase inhibitors. J. Enzyme Inhib. Med. Chem..

[8] Aziz M.A., Serya R.A.T., Lasheen D.S., Abouzid K.A.M. (2016). Furo[2,3-d]pyrimidine based derivatives as kinase inhibitors and anticancer agents. Future J. Pharm. Sci..

[9] Romeo R., Giofre S.V., Garozzo A., Bisignano B., Corsaro A., Chiacchio M.A. (2013). Synthesis and biological evaluation of furopyrimidine N,O-nucleosides. Bioorg. Med. Chem..

[10] Framski G., Wawrzyniak D., Jahnz-Wechmann Z., Szymanska-Michalak A., Barciszewski J., Boryski J., Kraszewski A., Stawinski J. New Applications of 6-Alkyl-2,3dihydrofurano[2,3-d]pyrimidin-2(1H)-one and 6-Alkyl-2,3-dihydropyrrolo[2,3-d]pyrimidin-2(3H,7H)-one Nucleosides: Anticancer Properties. Proceedings of the XXI Round Table on Nucleosides, Nucleotides and Nucleic Acids “Chemical Biology of Nucleic Acids”.

[11] Framski G., Wawrzyniak D., Jahnz-Wechmann Z., Szymanska-Michalak A., Kraszewski A., Barciszewski J., Boryski J., Stawinski J. (2016). Searching for anti-glioma activity. Ribonucleoside analogues with modifications in nucleobase and sugar moieties. Acta Biochim. Pol..

[12] Matyugina E., Logashenko E., Zenkova M., Kochetkov S., Khandazhinskaya A. (2015). 5′-Norcarbocyclic analogues of furano[2,3-d] pyrimidine nucleosides. Heterocycl. Commun..

[13] Matyugina E.S., Khandazhinskaya A.L., Chernousova L.N., Andreevskaya S.N., Smirnova T.G., Chizhov A.O., Karpenko I.L., Kochetkov S.N., Alexandrova L.A. (2012). The Synthesis and Antituberculosis Activity of 5’-Nor Carbocyclic Uracil Derivatives. Bioorg. Med. Chem..

[14] Khandazhinskaya A.L., Shirokova E.A., Shipitsin A.V., Karpenko I.L., Belanov E.F., Kukhanova M.K., Yasko M.V. (2006). Adenosine N1-oxide analogues as inhibitors of orthopox virus replication. Collect. Czech. Chem. Commun..

[15] Matyugina E.S., Khandazhinskaya A.L., Kochetkov S.N. (2012). Carbocyclic nucleoside analogues: Classification, target enzymes, mechanisms of action and synthesis. Russ. Chem. Rev..

[16] Ainai T., Wang Y.-G., Tokoro Y., Kobayashi Y. (2004). Highly Stereoselective Synthesis of Aristeromycin through Dihydroxylation of 4-Aryl-1-azido-2-cyclopentenes. J. Org. Chem..

[17] Fuertes M.A., Castilla J., Alonso C., Pérez J.M. (2002). Novel concepts in the development of platinum antitumor drugs. Curr. Med. Chem. Anticancer Agents.

