# Supplementary material for: Novel 5′-Norcarbocyclic Derivatives of Bicyclic Pyrrolo- and Furano[2,3-d]Pyrimidine Nucleosides
Source: Molecules. 2018 Oct 16;23(10):2654. doi: 10.3390/molecules23102654 (PMC6222425; doi:10.3390/molecules23102654)
Supplement: Supplementary file 1 [file molecules-23-02654-s001.pdf]

Novel 5'-norcarbocyclic derivatives of bicyclic pyrrolo- and furano[2,3-d]pyrimidine nucleosides

Anna A. Klimenko,[a] Elena S. Matyugina,[a] Evgeniya B. Logashenko,[b] Pavel N. Solyev,[a] Marina A. Zenkova,[b] Sergey N. Kochetkov,[a] Anastasiya L. Khandazhinskaya[a]

[a] *Engelhardt Institute of Molecular Biology, Russian Academy of Sciences, 32 Vavilov St., Moscow 119991, Russia*
[b] *Institute of Chemical Biology and Fundamental Medicine, 8 Lavrentiev Ave., Novosibirsk 630090, Russia*

Supplementary materials

Table of contents:

^{1}H NMR for compound **3a** p. 2
^{13}C NMR for compound **3a** p. 3
^{1}H NMR for compound **4a** p. 4
^{13}C NMR for compound **4a** p. 5
^{1}H NMR for compound **3b** p. 6
^{13}C NMR for compound **3b** p. 7
^{1}H NMR for compound **4b** p. 8
^{13}C NMR for compound **4b** p. 9
^{1}H NMR for compound **3c** p.10
^{13}C NMR for compound **3c** p.11
^{1}H NMR for compound **4c** p.12
^{13}C NMR for compound **4c** p.13
^{1}H NMR for compound **3d** p.14
^{13}C NMR for compound **3d** p.15
^{1}H NMR for compound **4d** p.16
^{13}C NMR for compound **4d** p.17
^{1}H NMR for compound **3e** p.18
^{13}C NMR for compound **3e** p.19
^{1}H NMR for compound **4e** p.20
^{13}C NMR for compound **4e** p.21
^{1}H NMR for compound **3f** p.22
^{13}C NMR for compound **3f** p.23
^{1}H NMR for compound **4f** p.24
^{13}C NMR for compound **4f** p.25
^{1}H NMR for compound **3g** p.26
^{13}C NMR for compound **3g** p.27
^{1}H NMR for compound **4g** p.28
^{13}C NMR for compound **4g** p.29
^{1}H NMR for compound **3h** p.30
^{13}C NMR for compound **3h** p.31
^{1}H NMR for compound **4h** p.32
^{13}C NMR for compound **4h** p.33
^{1}H NMR for compound **3i** p.34
^{13}C NMR for compound **3i** p.35
^{1}H NMR for compound **4i** p.36
^{13}C NMR for compound **4i** p.37
^{1}H NMR for compound **5** p.38
^{13}C NMR for compound **5** p.39

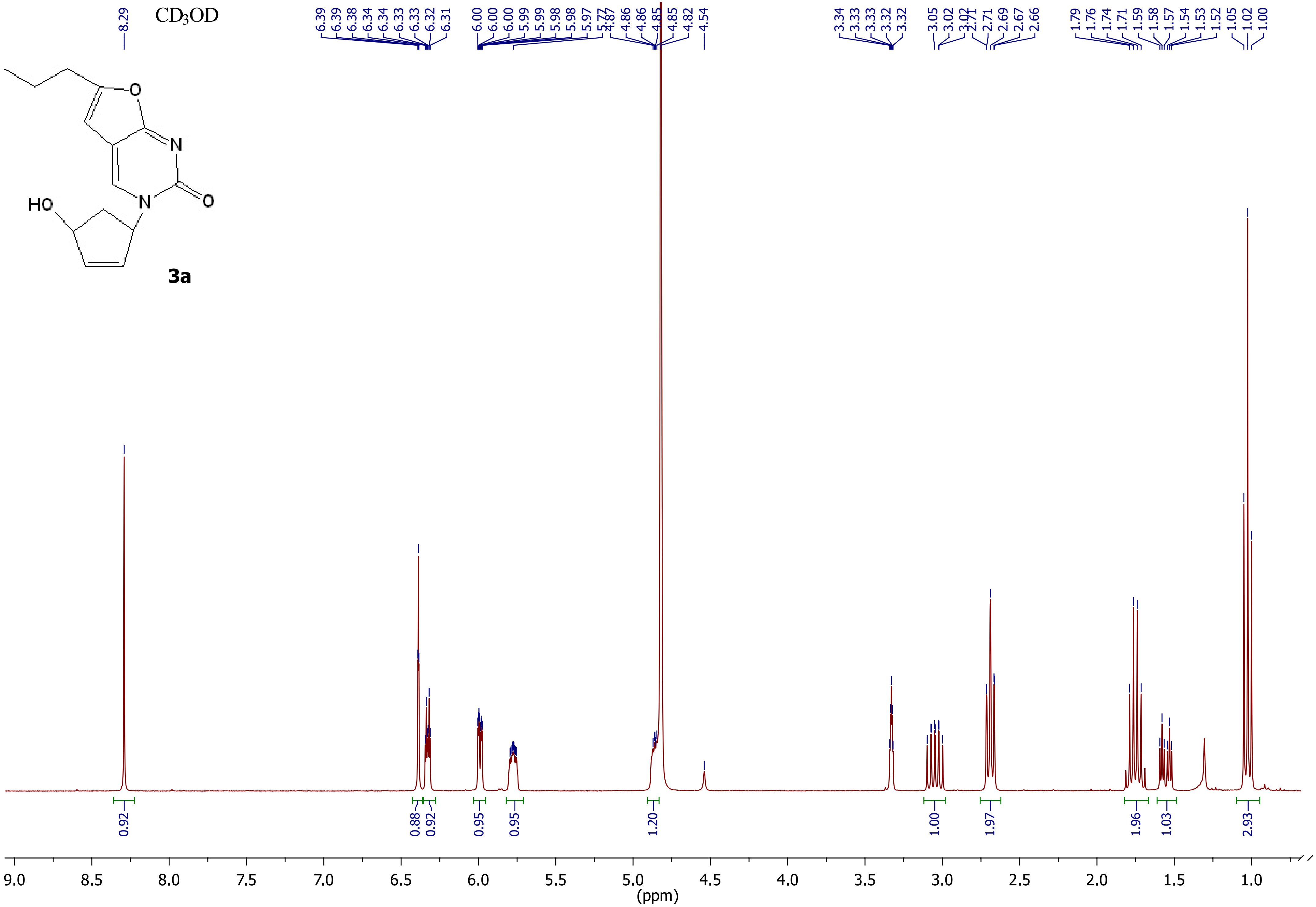
CD3OD
HO
O
N
N
O
3a
(ppm)

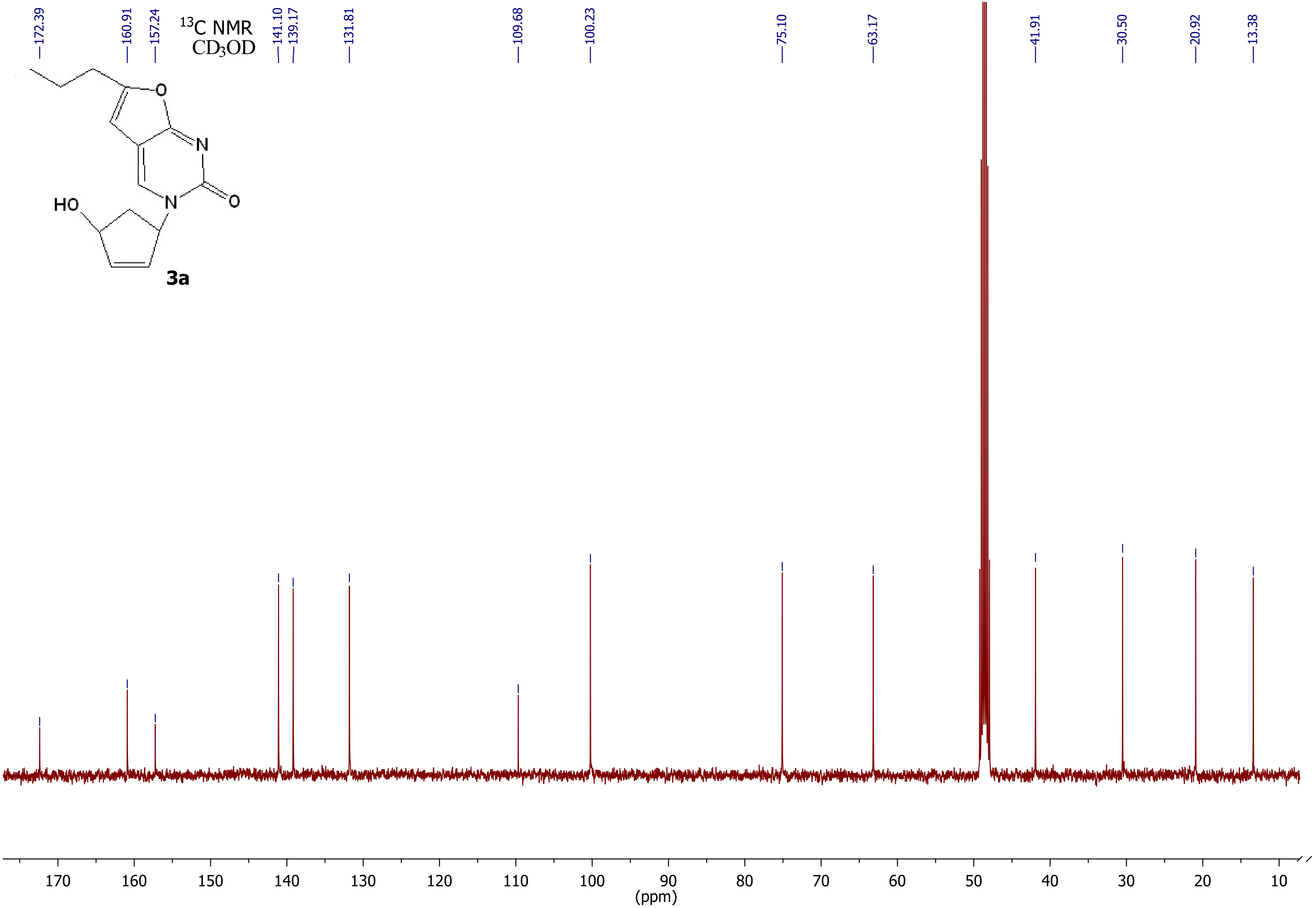

172.39
160.91
157.24
13C NMR
CD3OD
141.10
139.17
131.81
109.68
100.23
75.10
63.17
41.91
30.50
20.92
13.38
O
N
HO
N
O
3a
170
160
150
140
130
120
110
100
90
(ppm)
80
70
60
50
40
30
20
10

^{1}H NMR

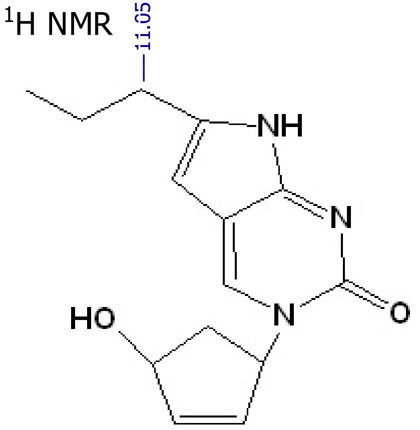

4a

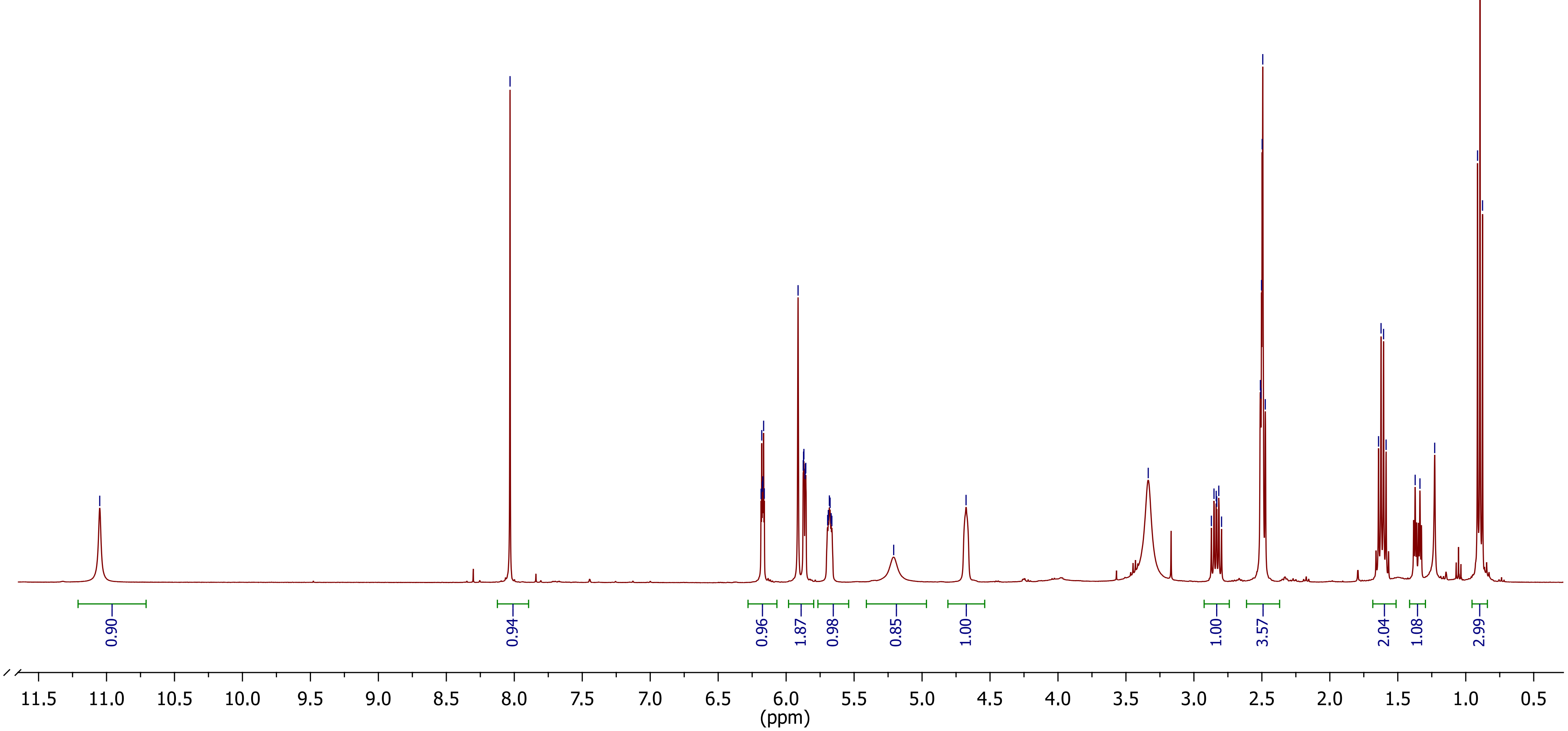

11.05
8.03
6.18
6.18
6.17
6.17
6.17
6.16
5.91
5.87
5.87
5.87
5.86
5.86
5.69
5.69
5.68
5.68
5.67
5.66
5.21
4.68
3.34
2.85
2.84
2.83
2.82
2.51
2.50
2.50
2.49
1.64
1.62
1.60
1.59
1.37
1.34
1.23
0.91
0.89
0.88
0.90
0.94
0.96
1.87
0.98
0.85
1.00
1.00
3.57
2.04
1.08
2.99
11.5
11.0
10.5
10.0
9.5
9.0
8.5
8.0
7.5
7.0
6.5
6.0
5.5
5.0
4.5
4.0
3.5
3.0
2.5
2.0
1.5
1.0
0.5
(ppm)

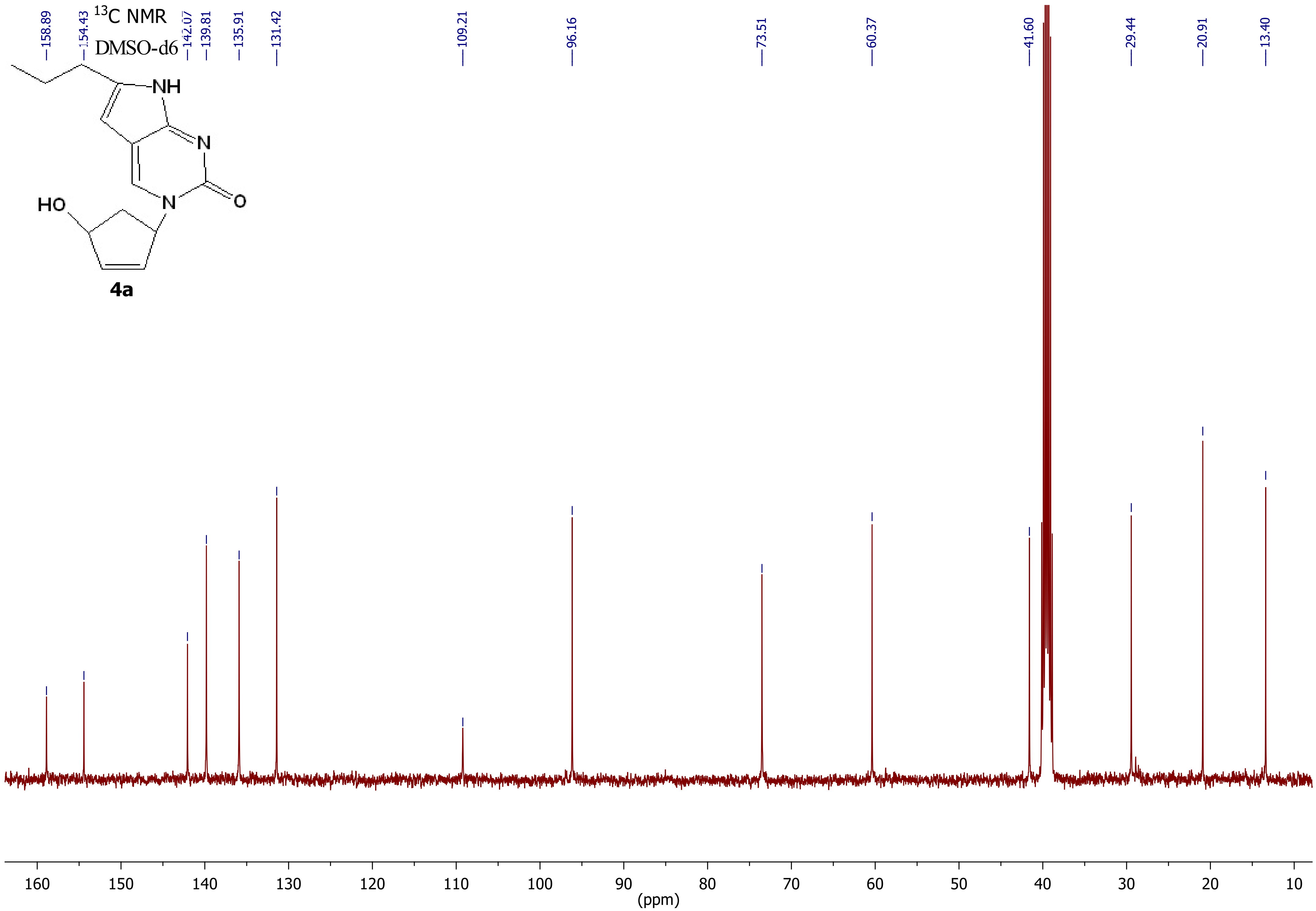

13C NMR
DMSO-d6
4a
158.89
154.43
142.07
139.81
135.91
131.42
109.21
96.16
73.51
60.37
41.60
29.44
20.91
13.40
(ppm)

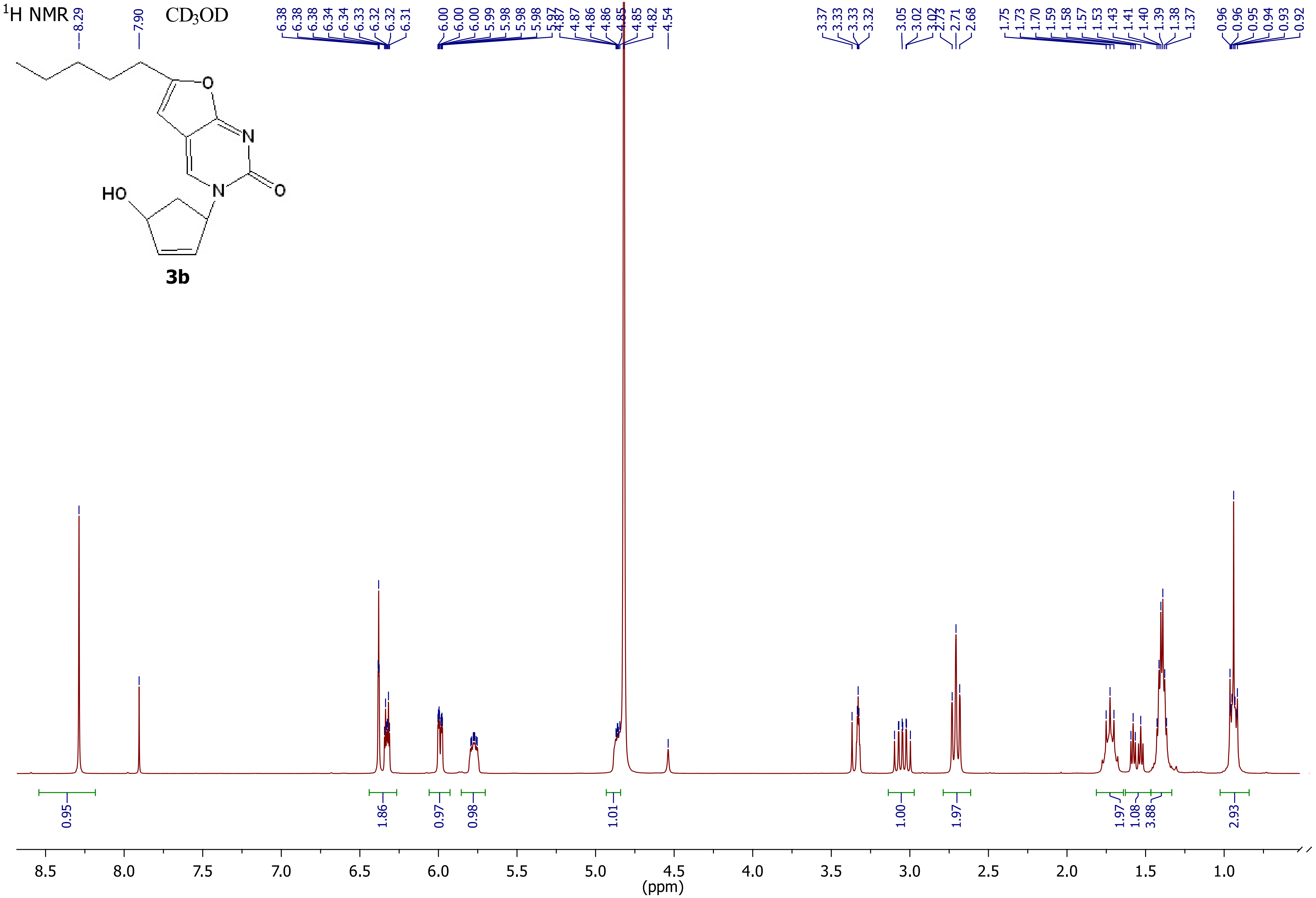
1H NMR
CD3OD
8.29
7.90
6.38 6.38 6.38 6.34 6.34 6.33 6.32 6.32 6.31
6.00 6.00 6.00 5.99 5.98 5.98 5.98 5.97
4.87 4.87 4.86 4.86 4.85 4.85 4.82
4.54
3.37 3.33 3.33 3.32
3.05 3.02 3.02
2.73 2.71 2.68
1.75 1.73 1.70 1.59 1.58 1.57 1.53 1.43 1.41 1.40 1.39 1.38 1.37
0.96 0.96 0.95 0.94 0.93 0.92
O
N
O
HO
N
3b
0.95
1.86
0.97
0.98
1.01
1.00
1.97
1.97
1.08
3.88
2.93
8.5 8.0 7.5 7.0 6.5 6.0 5.5 5.0 4.5 4.0 3.5 3.0 2.5 2.0 1.5 1.0
(ppm)

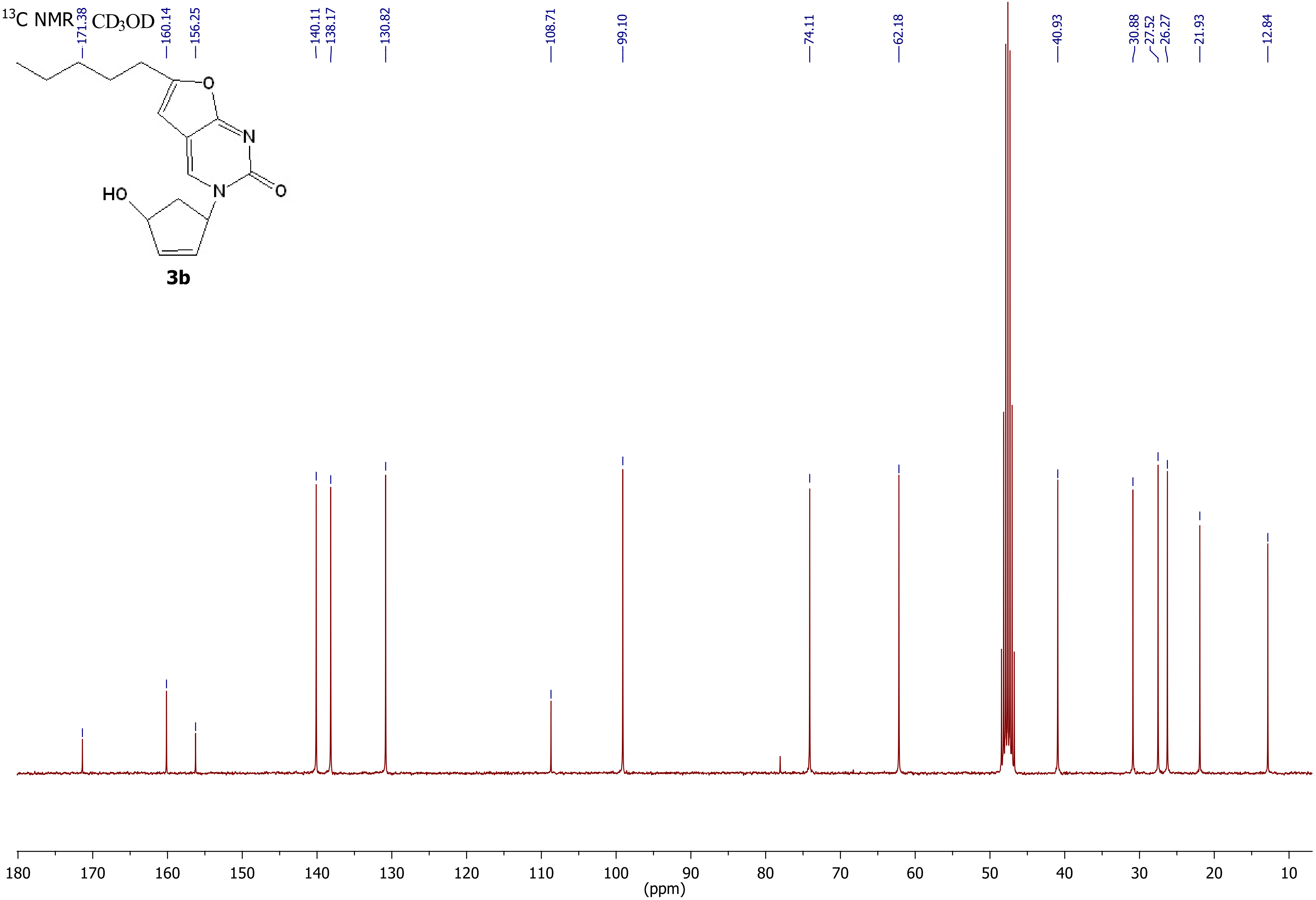

^{13}C NMR CD$_3$OD
171.38
160.14
156.25
140.11
138.17
130.82
108.71
99.10
74.11
62.18
40.93
30.88
27.52
26.27
21.93
12.84
O
N
N
O
HO
3b
180 170 160 150 140 130 120 110 100 90 80 70 60 50 40 30 20 10
(ppm)

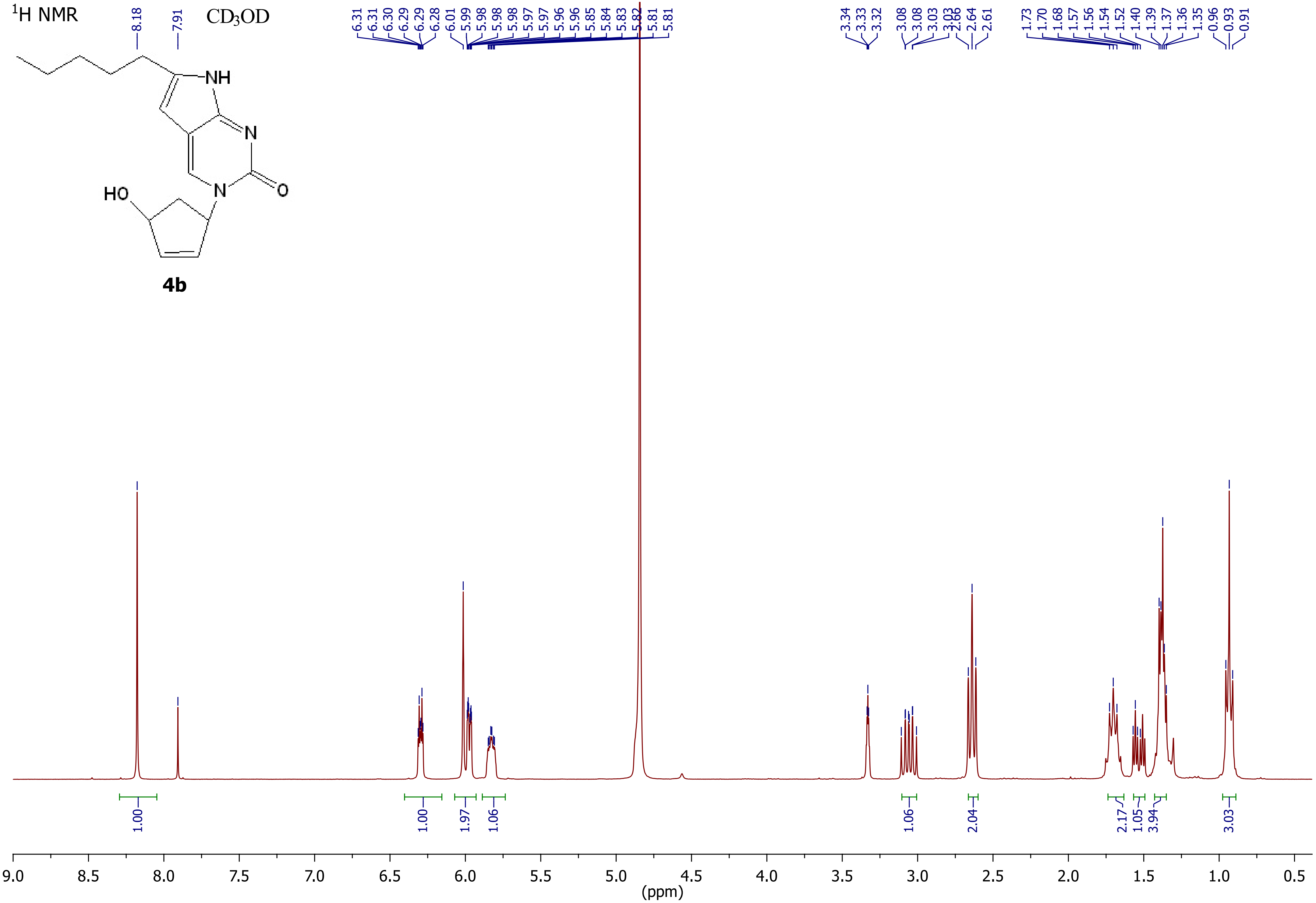

1H NMR
CD3OD
8.18
7.91
6.31 6.31 6.30 6.29 6.29 6.28 6.01 5.99 5.98 5.98 5.98 5.97 5.97 5.96 5.96 5.85 5.84 5.83 5.82 5.81 5.81
3.34 3.33 3.32 3.08 3.08 3.03 3.03 2.66 2.64 2.61
1.73 1.70 1.68 1.57 1.56 1.54 1.52 1.40 1.39 1.37 1.36 1.35 0.96 0.93 0.91
NH
N
O
HO
N
4b
1.00
1.00
1.97
1.06
1.06
2.04
2.17
1.05
3.94
3.03
9.0 8.5 8.0 7.5 7.0 6.5 6.0 5.5 5.0 4.5 4.0 3.5 3.0 2.5 2.0 1.5 1.0 0.5
(ppm)

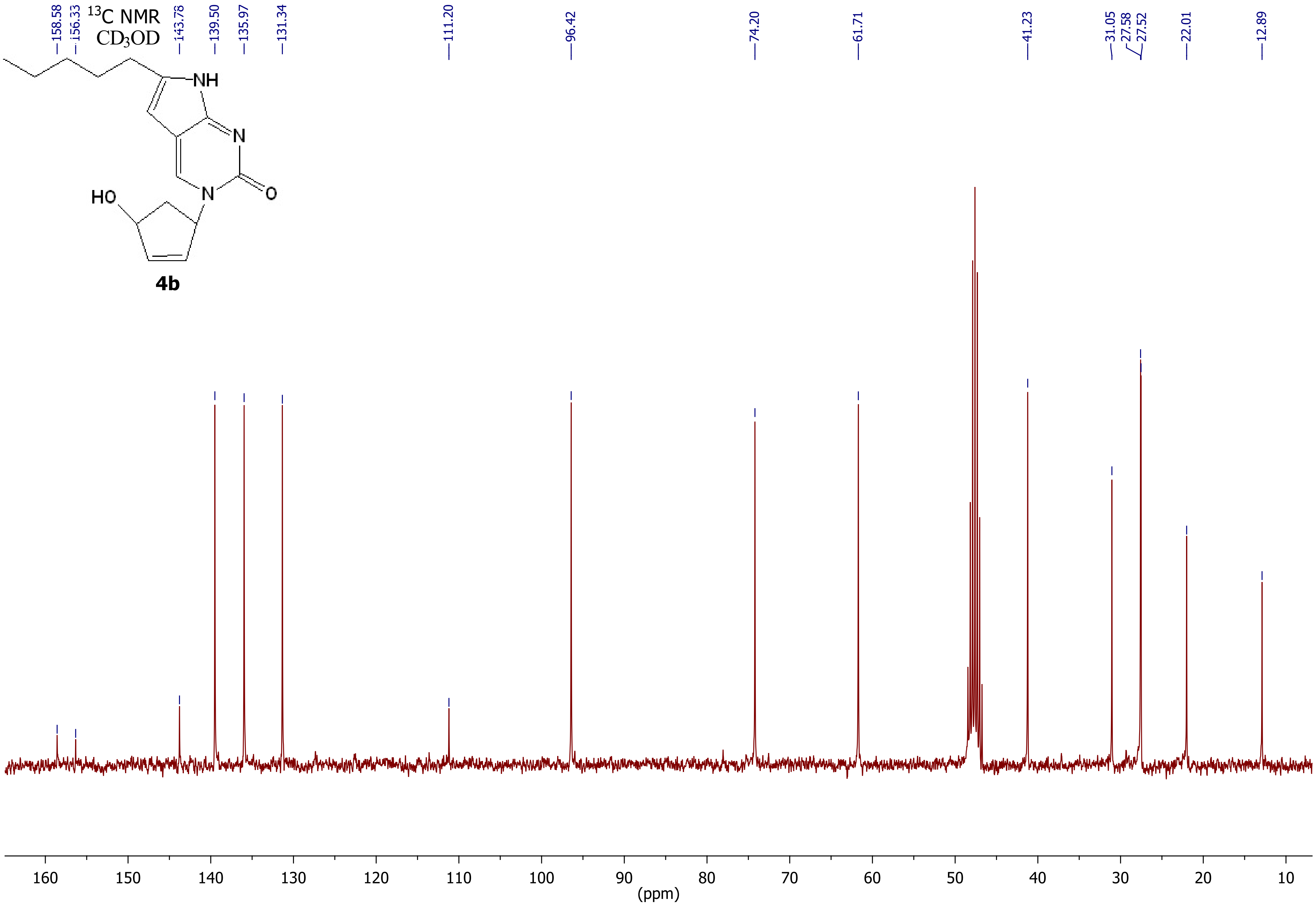

^{13}C NMR
CD_3OD
4b
NH
N
N
O
HO
158.58
156.33
143.78
139.50
135.97
131.34
111.20
96.42
74.20
61.71
41.23
31.05
27.58
27.52
22.01
12.89
160
150
140
130
120
110
100
90
80
70
60
50
40
30
20
10
(ppm)

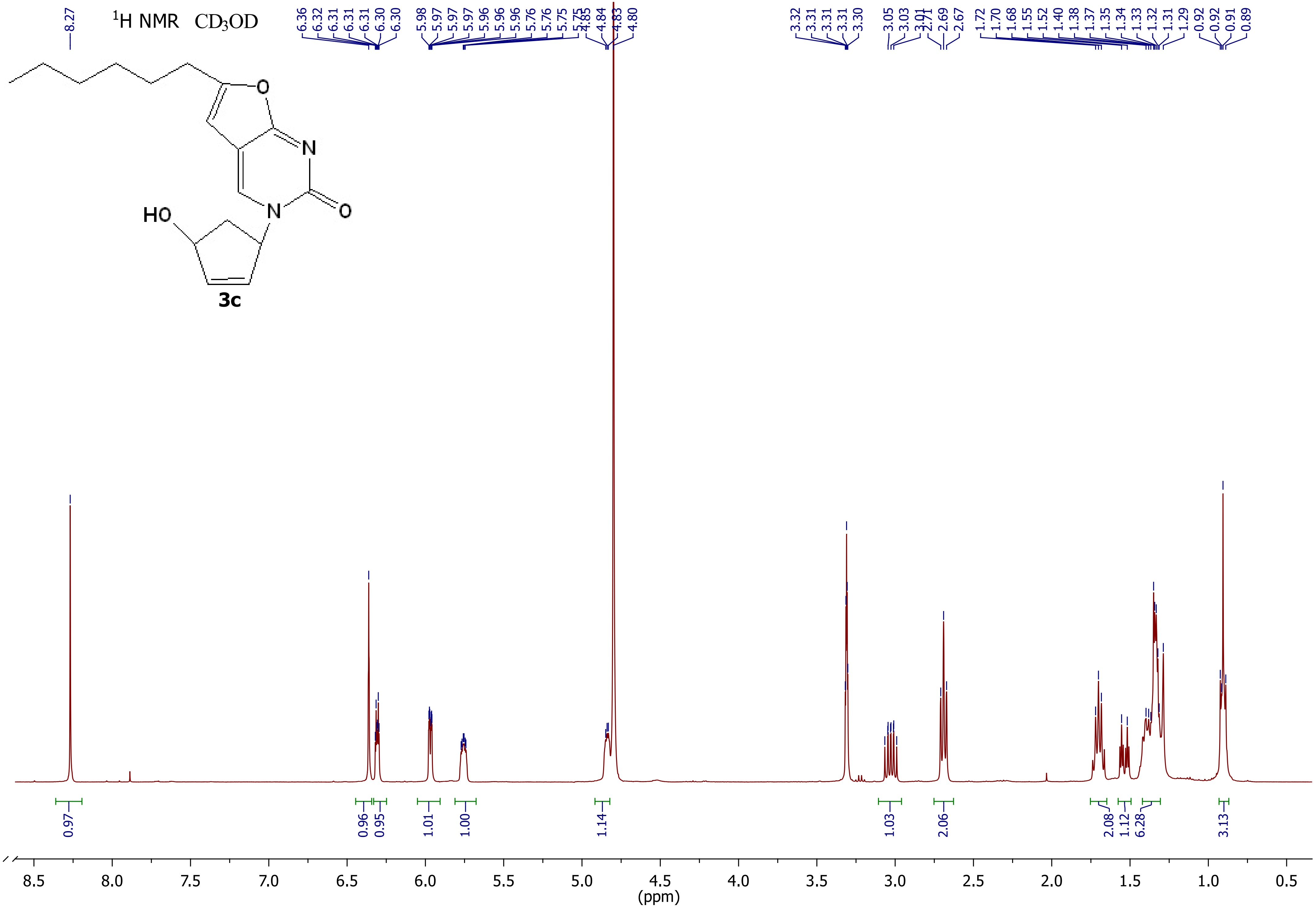

1H NMR CD3OD
HO
O
N
N
O
3c
8.27
6.36 6.32 6.31 6.31 6.31 6.30 6.30
5.98 5.97 5.97 5.97 5.96 5.96 5.96 5.76 5.76 5.75 5.75 4.85 4.84 4.83 4.80
3.32 3.31 3.31 3.31 3.30
3.05 3.03 3.01 2.71 2.69 2.67
1.72 1.70 1.68 1.55 1.52 1.40 1.38 1.37 1.35 1.34 1.33 1.32 1.31 1.29 0.92 0.92 0.91 0.89
0.97 0.96 0.95 1.01 1.00 1.14 1.03 2.06 2.08 1.12 6.28 3.13
8.5 8.0 7.5 7.0 6.5 6.0 5.5 5.0 4.5 4.0 3.5 3.0 2.5 2.0 1.5 1.0 0.5
(ppm)

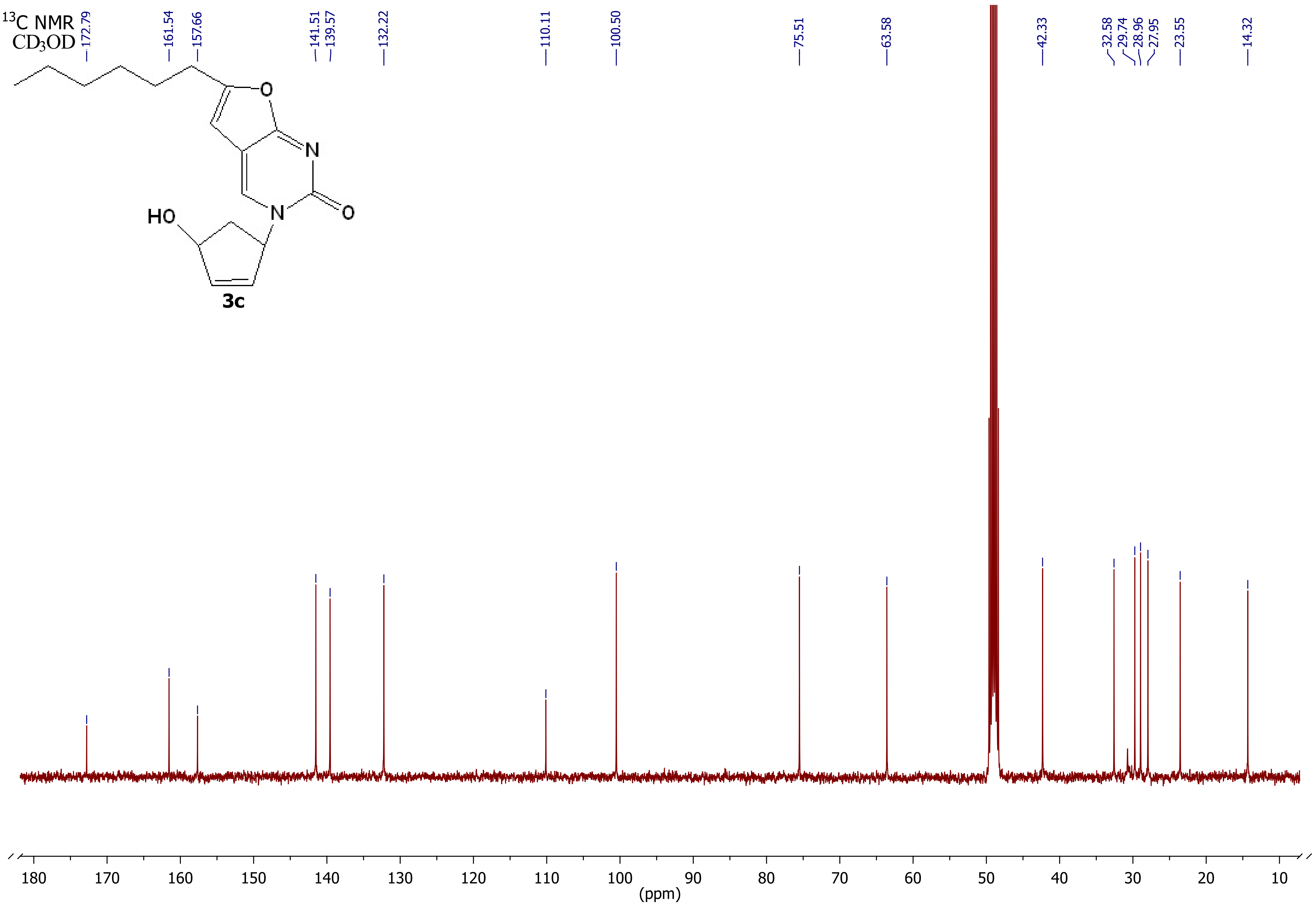

13C NMR
CD3OD
172.79
161.54
157.66
141.51
139.57
132.22
110.11
100.50
75.51
63.58
42.33
32.58
29.74
28.96
27.95
23.55
14.32
HO
O
N
N
O
3c
180
170
160
150
140
130
120
110
100
90
80
70
60
50
40
30
20
10
(ppm)

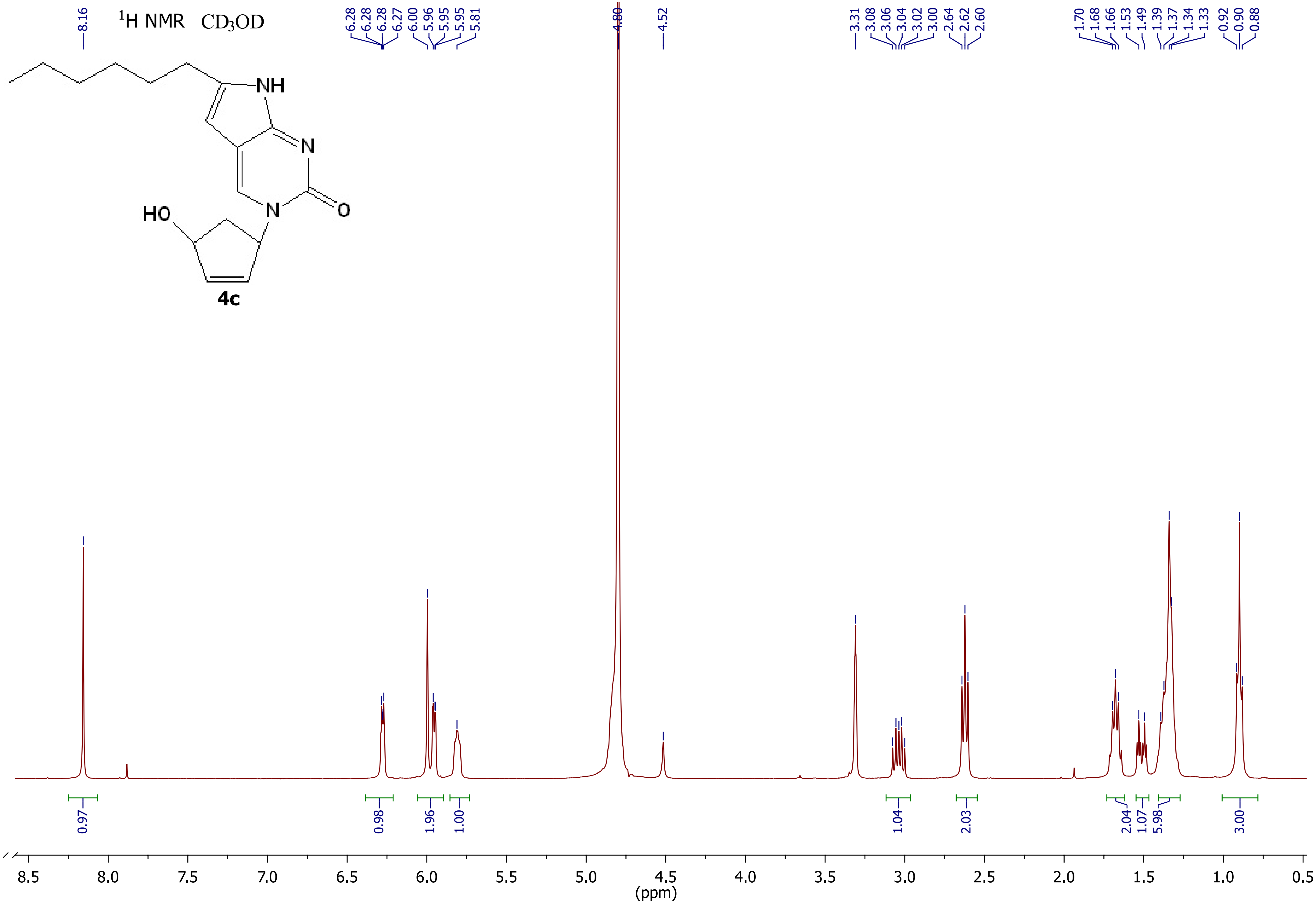

1H NMR CD3OD
NH
N
O
N
HO
4c
8.16
6.28 6.28 6.28 6.27 6.00 5.96 5.95 5.95 5.81
4.80
4.52
3.31 3.08 3.06 3.04 3.02 3.00 2.64 2.62 2.60
1.70 1.68 1.66 1.53 1.49 1.39 1.37 1.34 1.33 0.92 0.90 0.88
0.97
0.98
1.96
1.00
1.04
2.03
2.04
1.07
5.98
3.00
8.5 8.0 7.5 7.0 6.5 6.0 5.5 5.0 4.5 4.0 3.5 3.0 2.5 2.0 1.5 1.0 0.5
(ppm)

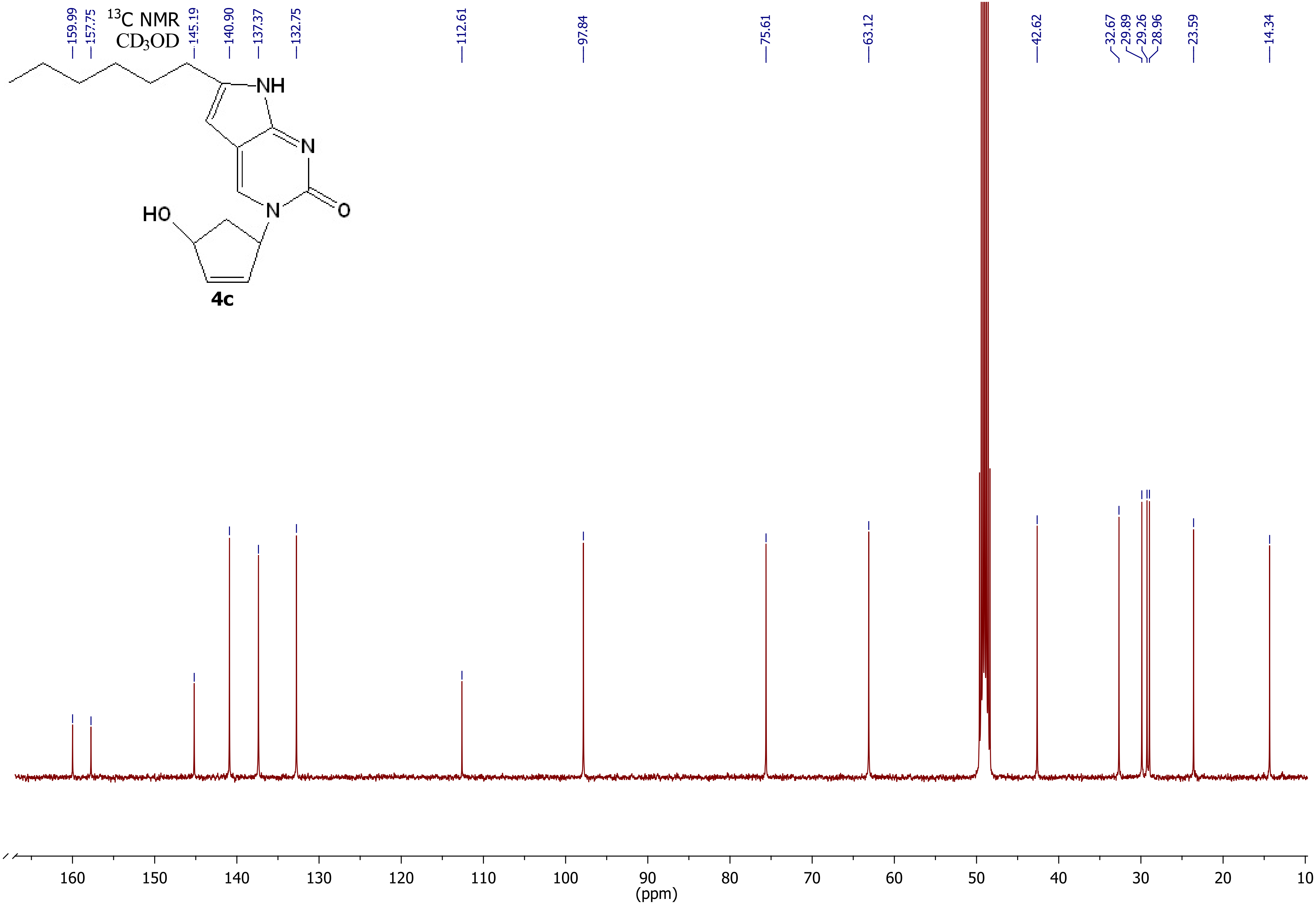

13C NMR
CD3OD
159.99
157.75
145.19
140.90
137.37
132.75
112.61
97.84
75.61
63.12
42.62
32.67
29.89
29.26
28.96
23.59
14.34
NH
N
O
N
HO
160
150
140
130
120
110
100
90
80
70
60
50
40
30
20
10
(ppm)

4c

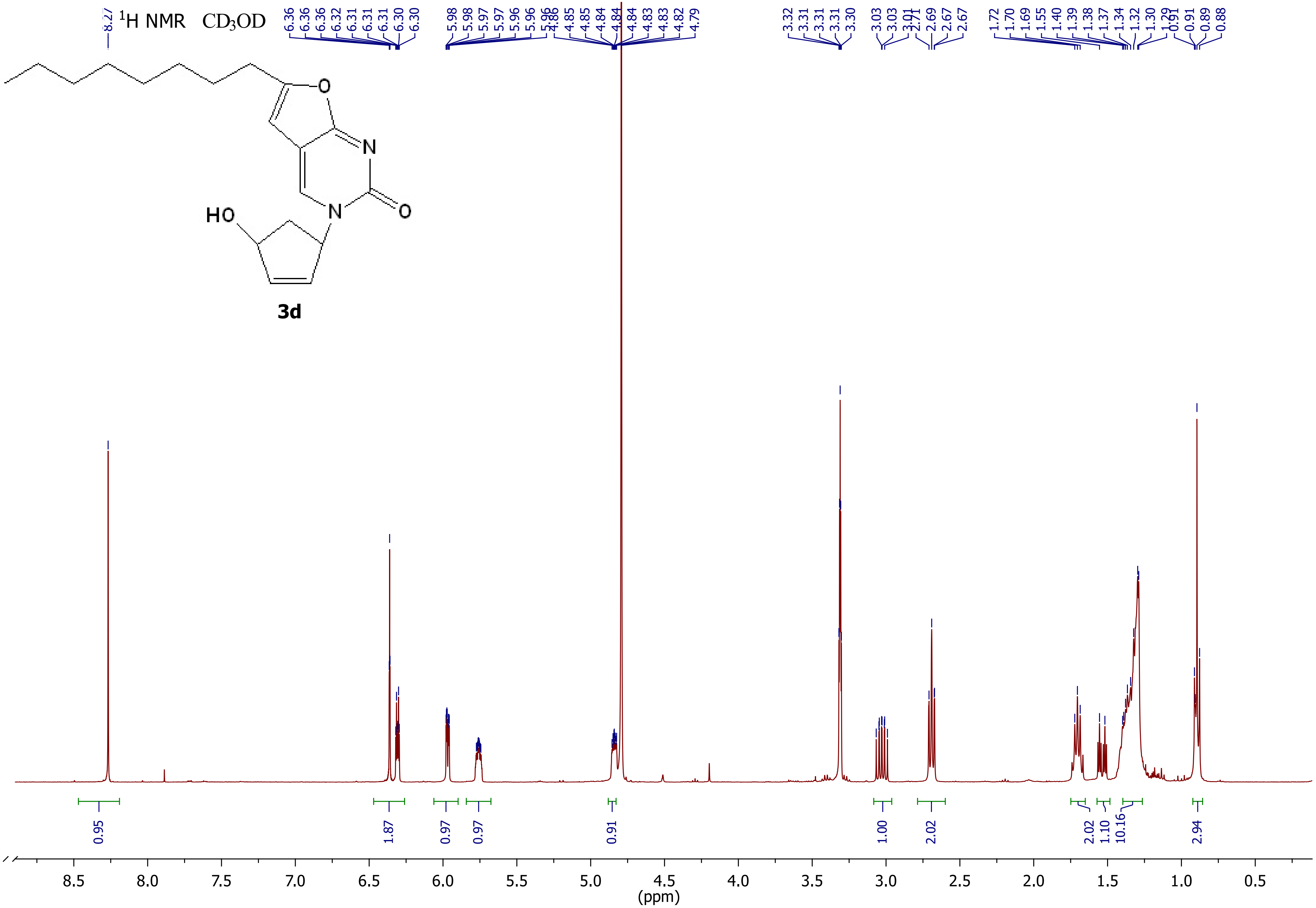

1H NMR CD3OD
HO
N
O
3d
8.5
8.0
7.5
7.0
6.5
6.0
5.5
5.0
4.5
4.0
3.5
3.0
2.5
2.0
1.5
1.0
0.5
(ppm)
0.95
1.87
0.97
0.97
0.91
1.00
2.02
2.02
1.10
10.16
2.94

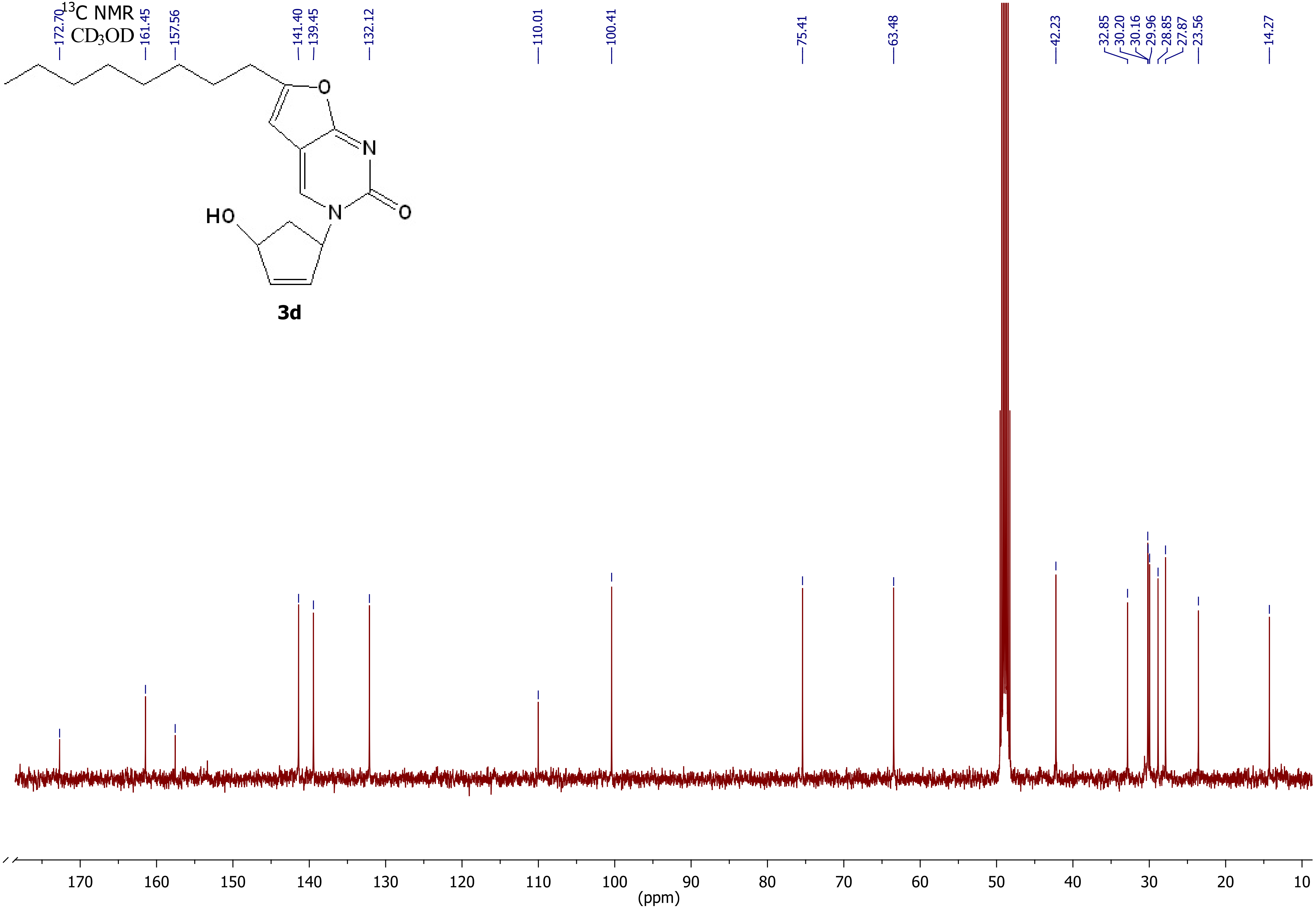

13C NMR
CD3OD
172.70
161.45
157.56
141.40
139.45
132.12
110.01
100.41
75.41
63.48
42.23
32.85
30.20
30.16
29.96
28.85
27.87
23.56
14.27
HO
N
O
3d
170
160
150
140
130
120
110
100
90
80
70
60
50
40
30
20
10
(ppm)

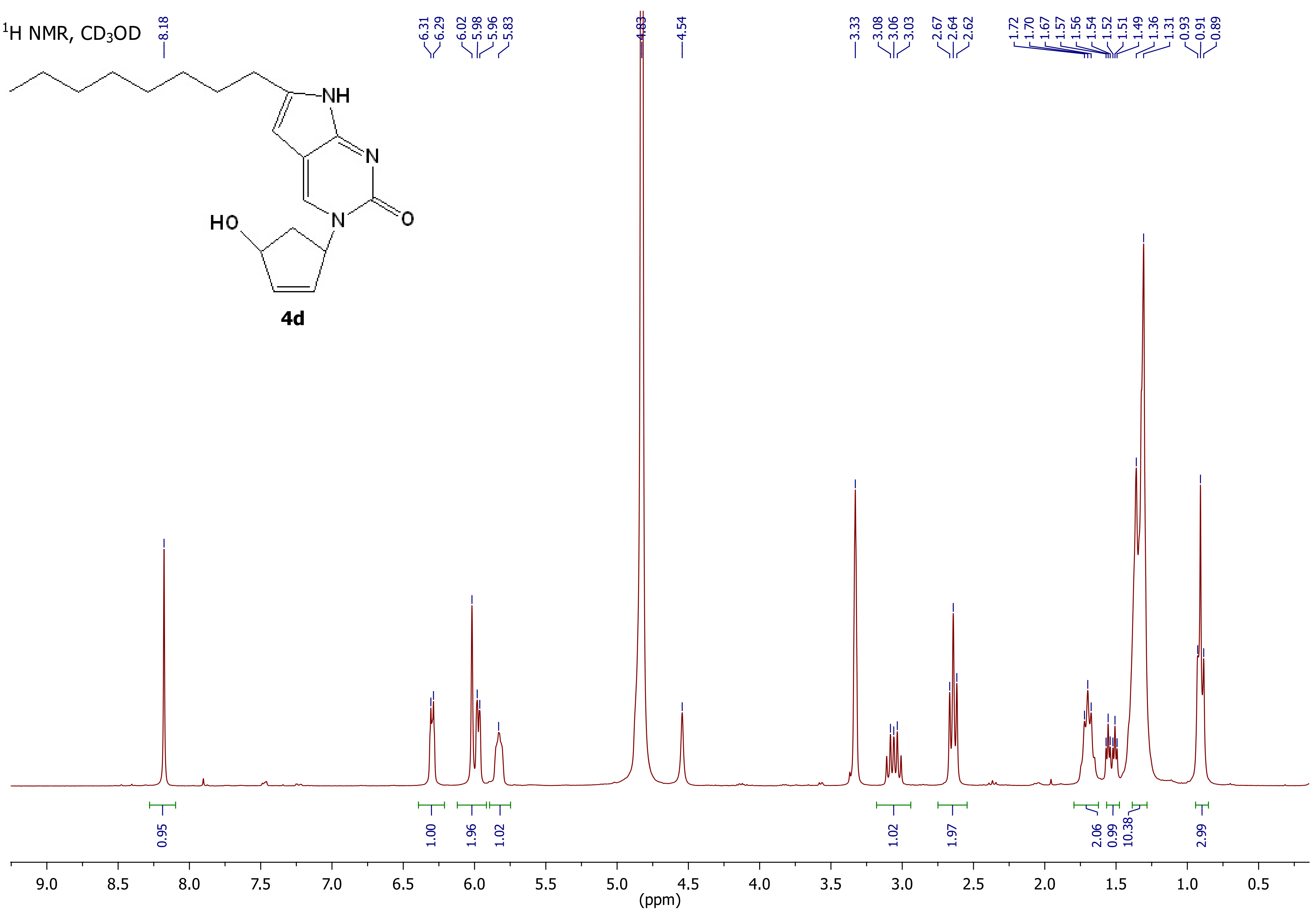

^{1}H NMR, CD_3OD
8.18
6.31 6.29 6.02 5.98 5.96 5.83
4.83 4.54
3.33 3.08 3.06 3.03 2.67 2.64 2.62
1.72 1.70 1.67 1.57 1.56 1.54 1.52 1.51 1.49 1.36 1.31 0.93 0.91 0.89
NH
N
O
N
HO
4d
0.95 1.00 1.96 1.02 1.02 1.97 2.06 0.99 10.38 2.99
9.0 8.5 8.0 7.5 7.0 6.5 6.0 5.5 5.0 4.5 4.0 3.5 3.0 2.5 2.0 1.5 1.0 0.5
(ppm)

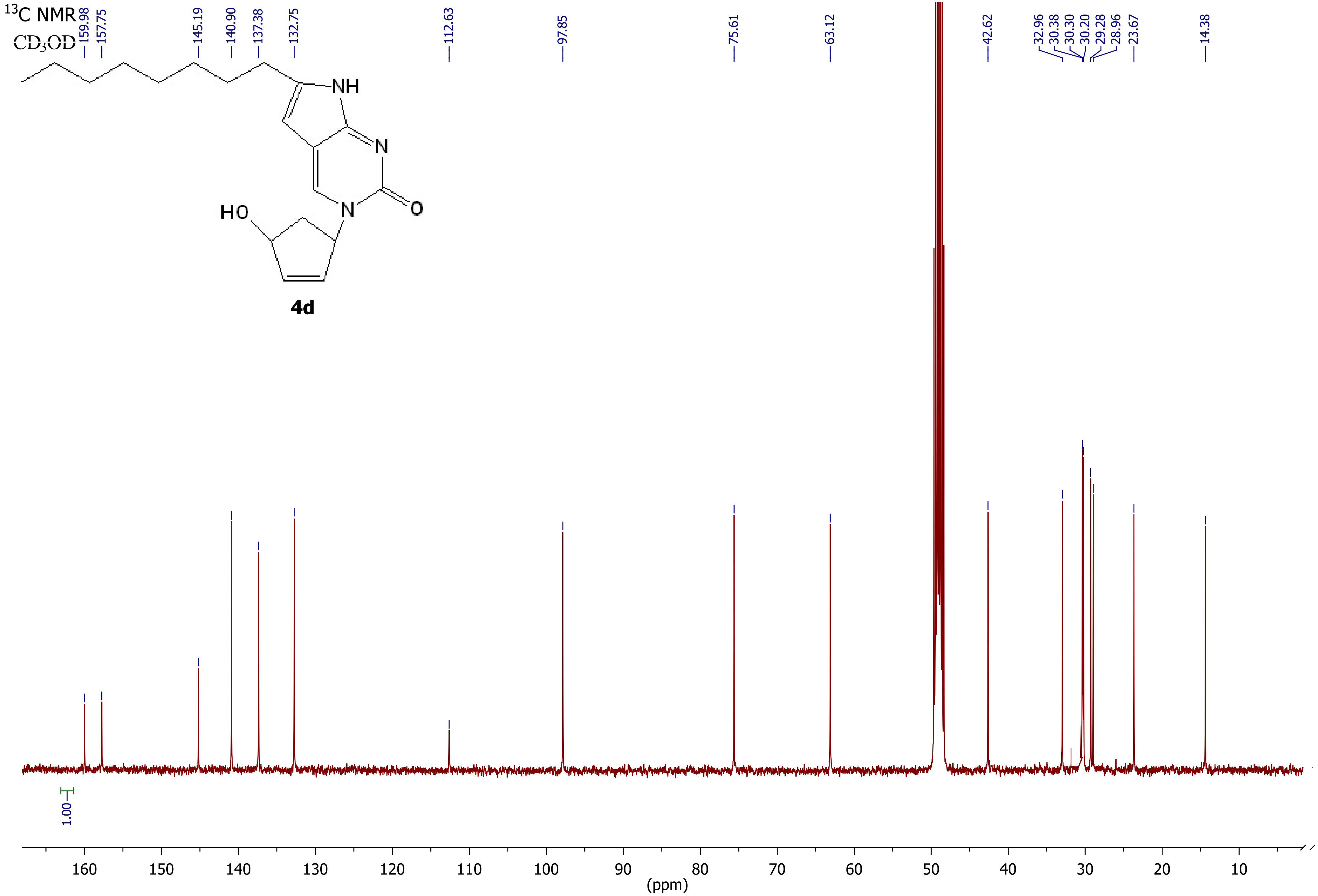

13C NMR
CD3OD
159.98
157.75
145.19
140.90
137.38
132.75
112.63
97.85
75.61
63.12
42.62
32.96
30.38
30.30
30.20
29.28
28.96
23.67
14.38
NH
N
N
O
HO
4d
1.00
160 150 140 130 120 110 100 90 80 70 60 50 40 30 20 10
(ppm)

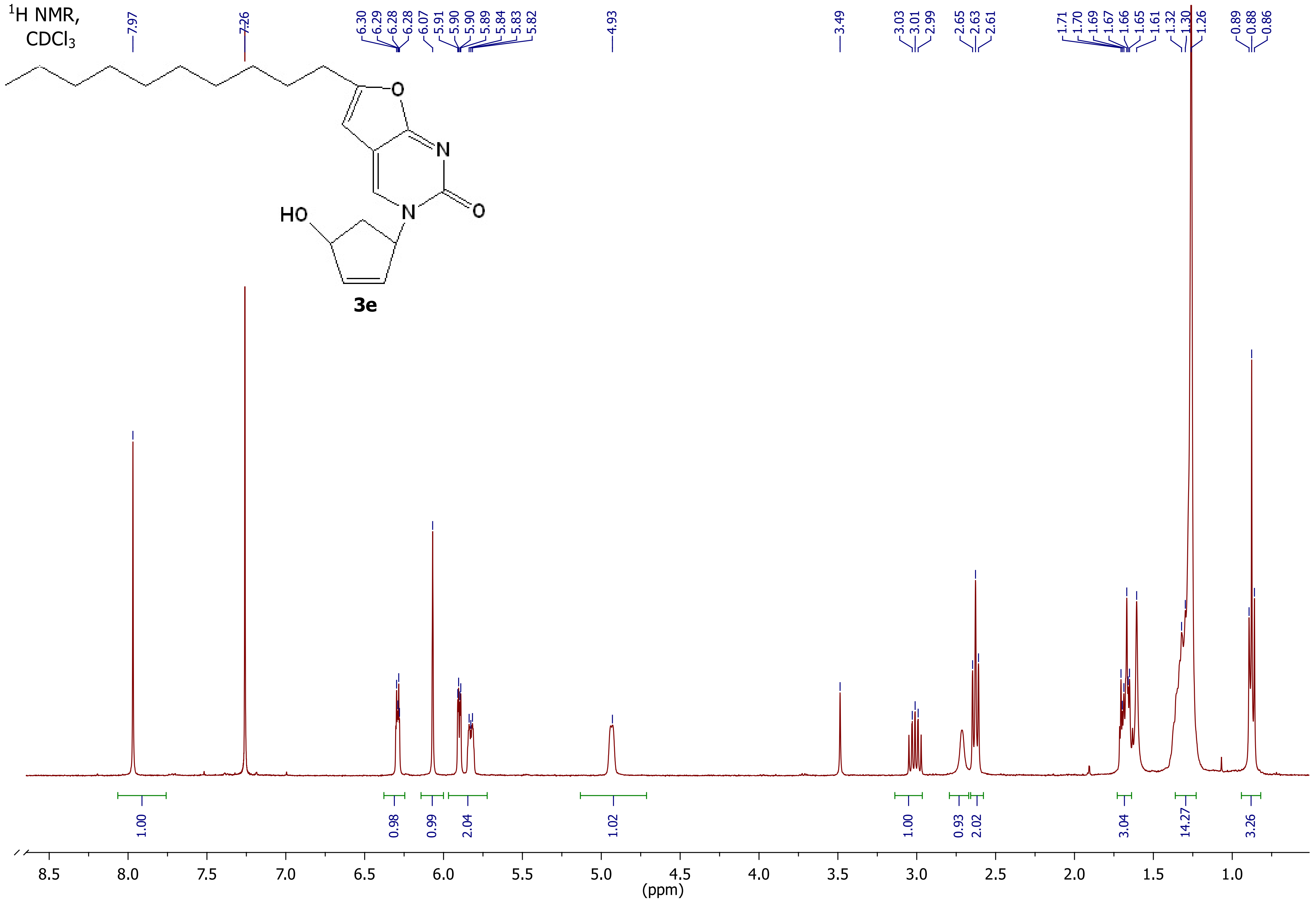

1H NMR,
CDCl3
3e
HO
N
N
O
O
7.97
7.26
6.30 6.29 6.28 6.28 6.07 5.91 5.90 5.90 5.89 5.84 5.83 5.82
4.93
3.49
3.03 3.01 2.99 2.65 2.63 2.61
1.71 1.70 1.69 1.67 1.66 1.65 1.61 1.32 1.30 1.26
0.89 0.88 0.86
1.00 0.98 0.99 2.04 1.02 1.00 0.93 2.02 3.04 14.27 3.26
8.5 8.0 7.5 7.0 6.5 6.0 5.5 5.0 4.5 4.0 3.5 3.0 2.5 2.0 1.5 1.0
(ppm)

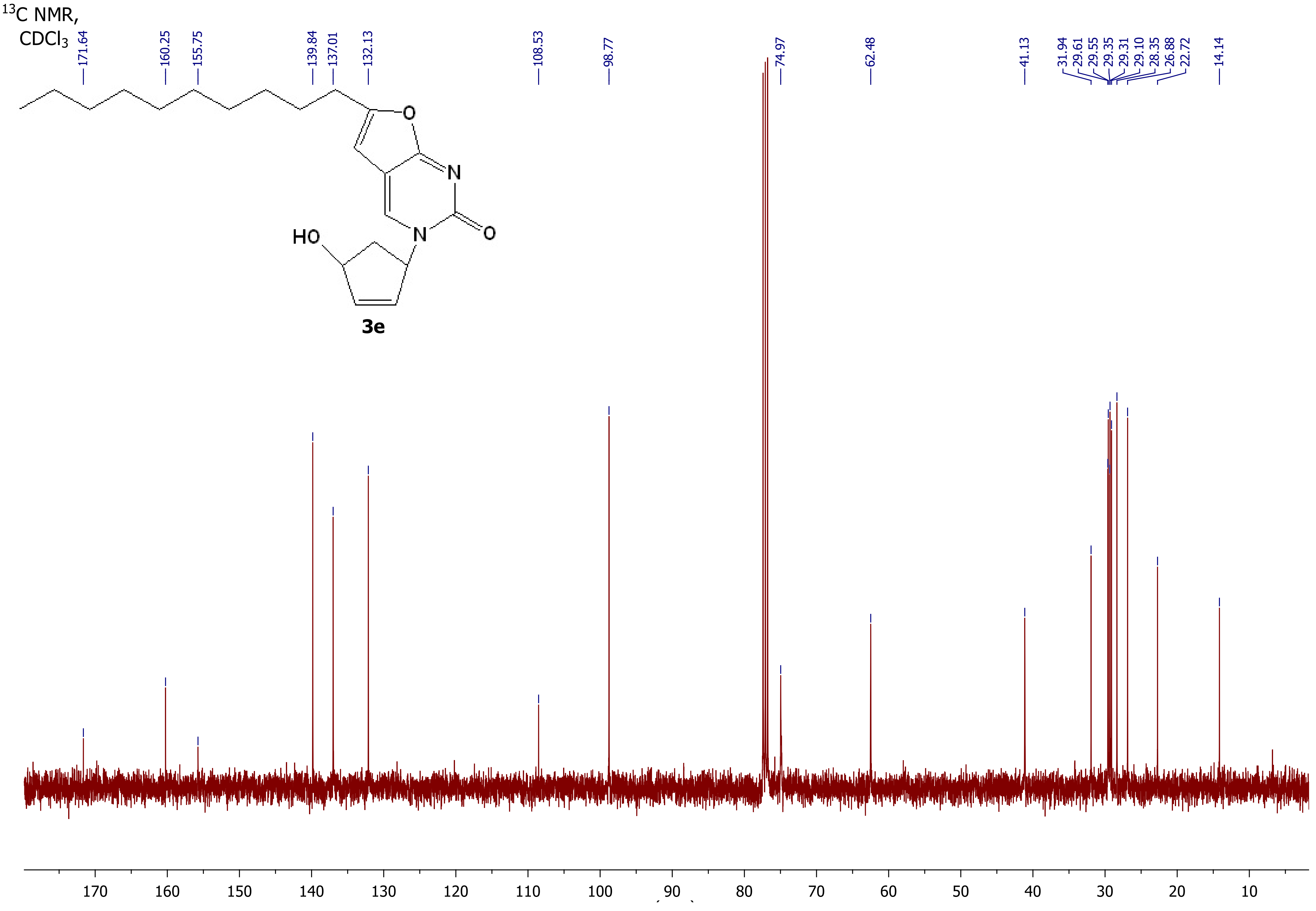

13C NMR,
CDCl3
171.64
160.25
155.75
139.84
137.01
132.13
108.53
98.77
74.97
62.48
41.13
31.94
29.61
29.55
29.35
29.31
29.10
28.35
26.88
22.72
14.14
HO
O
N
N
O
3e
170
160
150
140
130
120
110
100
90
80
70
60
50
40
30
20
10

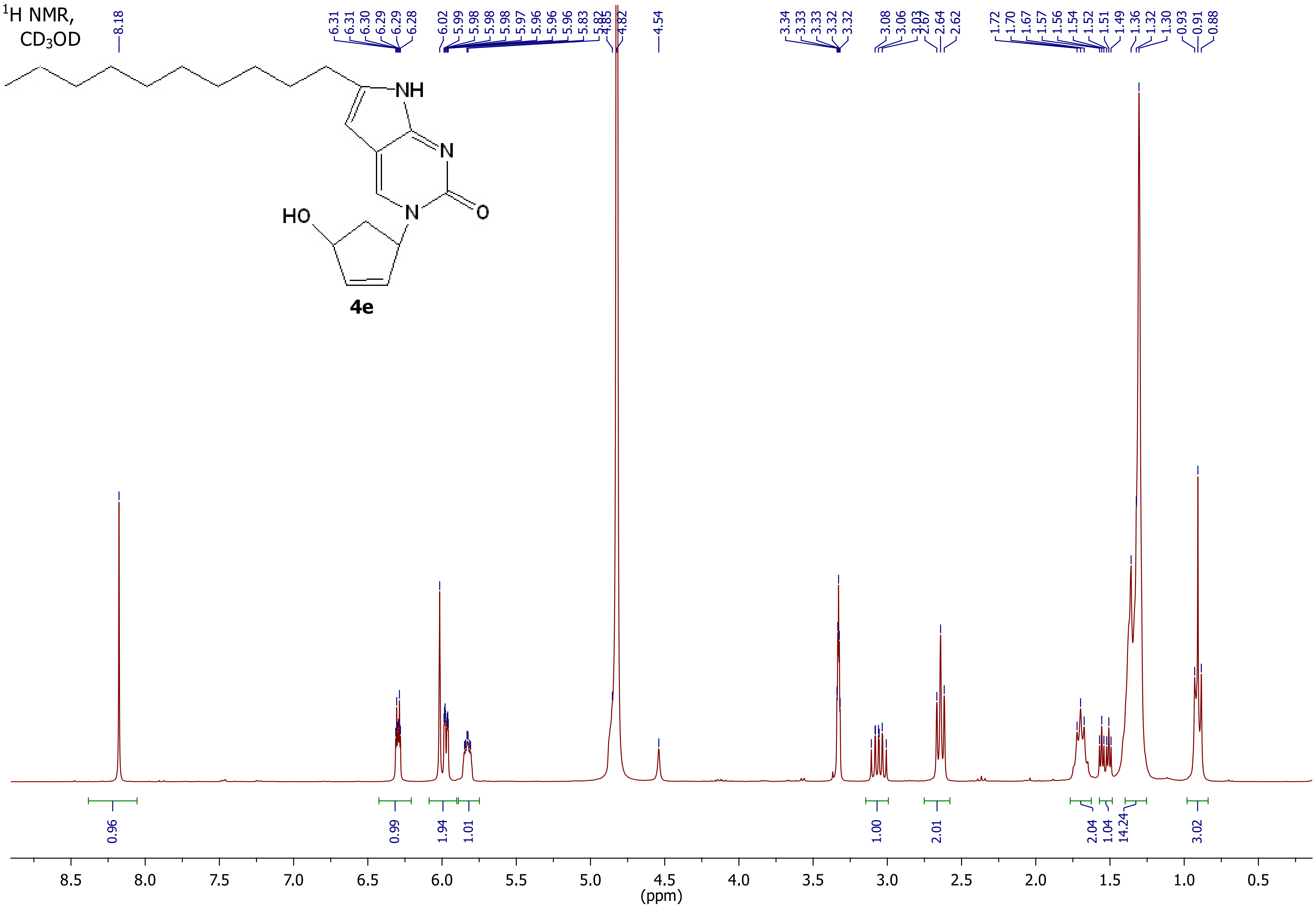

^{1}H NMR,
CD_3OD
4e
NH
N
N
O
HO
8.18
6.31 6.31 6.30 6.29 6.29 6.28
6.02 5.99 5.98 5.98 5.98 5.97 5.96 5.96 5.96 5.83 5.82 4.85 4.82
4.54
3.34 3.33 3.33 3.32 3.32
3.08 3.06 3.03 2.67 2.64 2.62
1.72 1.70 1.67 1.57 1.56 1.54 1.52 1.51 1.49 1.36 1.32 1.30 0.93 0.91 0.88
0.96
0.99
1.94
1.01
1.00
2.01
2.04
1.04
14.24
3.02
8.5 8.0 7.5 7.0 6.5 6.0 5.5 5.0 4.5 4.0 3.5 3.0 2.5 2.0 1.5 1.0 0.5
(ppm)

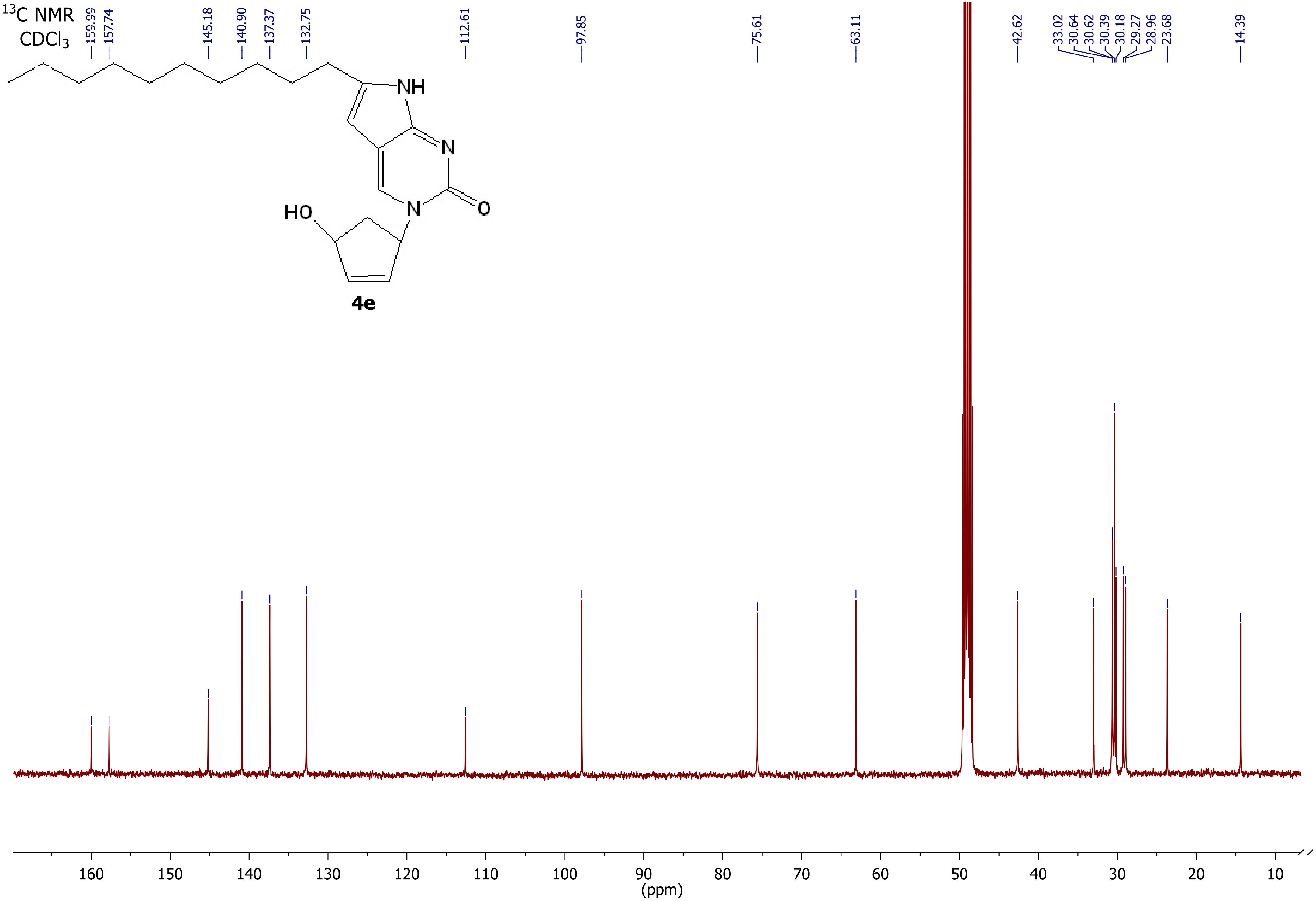

^{13}C NMR
$CDCl_3$
159.99
157.74
145.18
140.90
137.37
132.75
112.61
97.85
75.61
63.11
42.62
33.02
30.64
30.62
30.39
30.18
29.27
28.96
23.68
14.39
NH
N
N
O
HO
4e
160
150
140
130
120
110
100
90
80
70
60
50
40
30
20
10
(ppm)

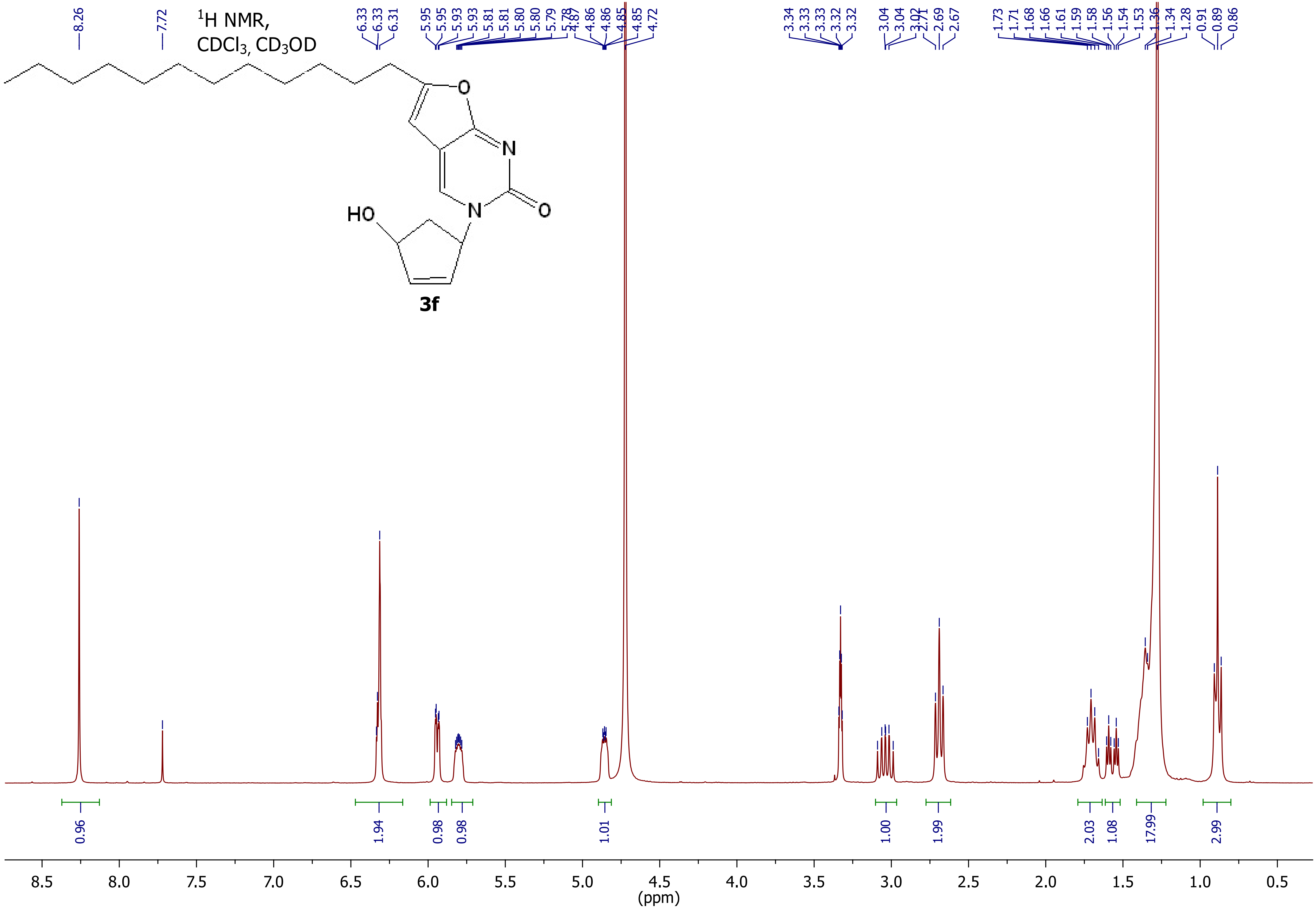

1H NMR,
CDCl3, CD3OD
HO
O
N
N
O
3f
8.26
7.72
6.33
6.33
6.31
5.95
5.95
5.93
5.93
5.81
5.81
5.80
5.80
5.79
5.78
4.87
4.86
4.86
4.85
4.85
4.72
3.34
3.33
3.33
3.32
3.32
3.04
3.04
3.02
2.71
2.69
2.67
1.73
1.71
1.68
1.66
1.61
1.59
1.58
1.56
1.54
1.53
1.36
1.34
1.28
0.91
0.89
0.86
0.96
1.94
0.98
0.98
1.01
1.00
1.99
2.03
1.08
17.99
2.99
8.5
8.0
7.5
7.0
6.5
6.0
5.5
5.0
4.5
4.0
3.5
3.0
2.5
2.0
1.5
1.0
0.5
(ppm)

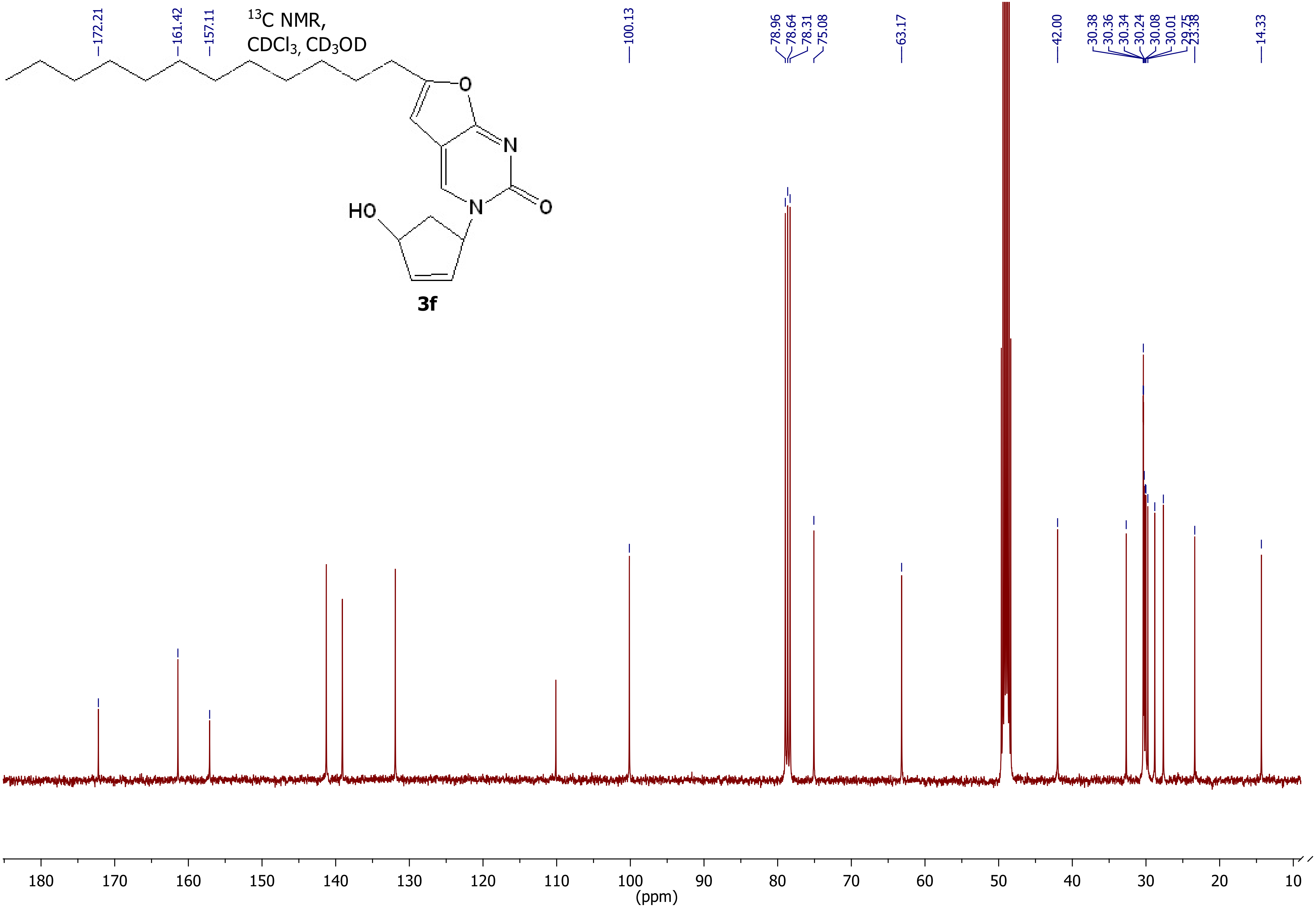

13C NMR,
CDCl3, CD3OD
HO
O
N
N
O
3f
172.21
161.42
157.11
100.13
78.96
78.64
78.31
75.08
63.17
42.00
30.38
30.36
30.34
30.24
30.08
30.01
29.75
23.38
14.33
(ppm)

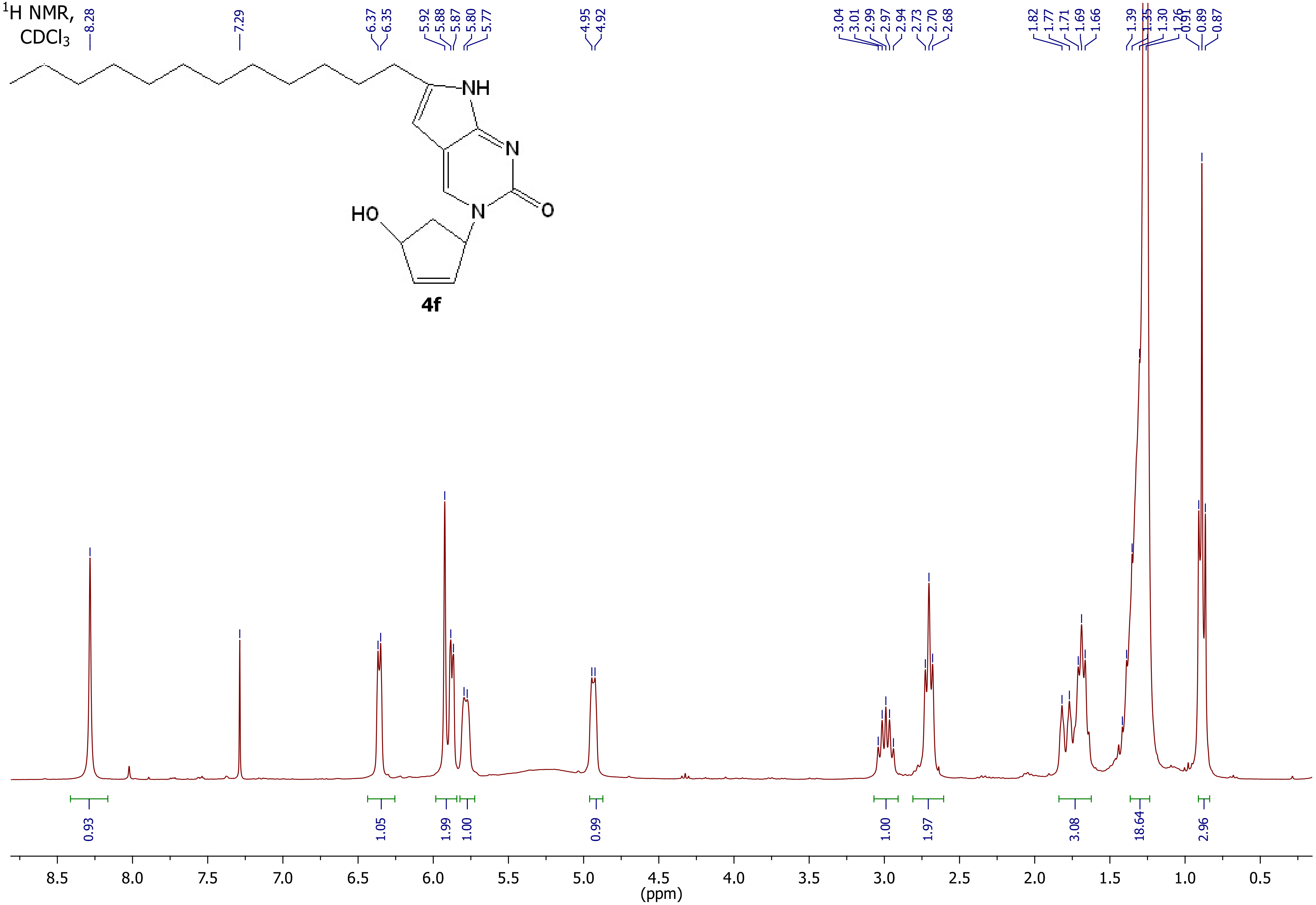

^{1}H NMR, CDCl$_3$
8.28
7.29
6.37 6.35
5.92 5.88 5.87 5.80 5.77
4.95 4.92
3.04 3.01 2.99 2.97 2.94
2.73 2.70 2.68
1.82 1.77 1.71 1.69 1.66
1.39 1.35 1.30 1.26 0.91 0.89 0.87
NH
N
O
N
HO
4f
0.93
1.05
1.99
1.00
0.99
1.00
1.97
3.08
18.64
2.96
8.5 8.0 7.5 7.0 6.5 6.0 5.5 5.0 4.5 4.0 3.5 3.0 2.5 2.0 1.5 1.0 0.5
(ppm)

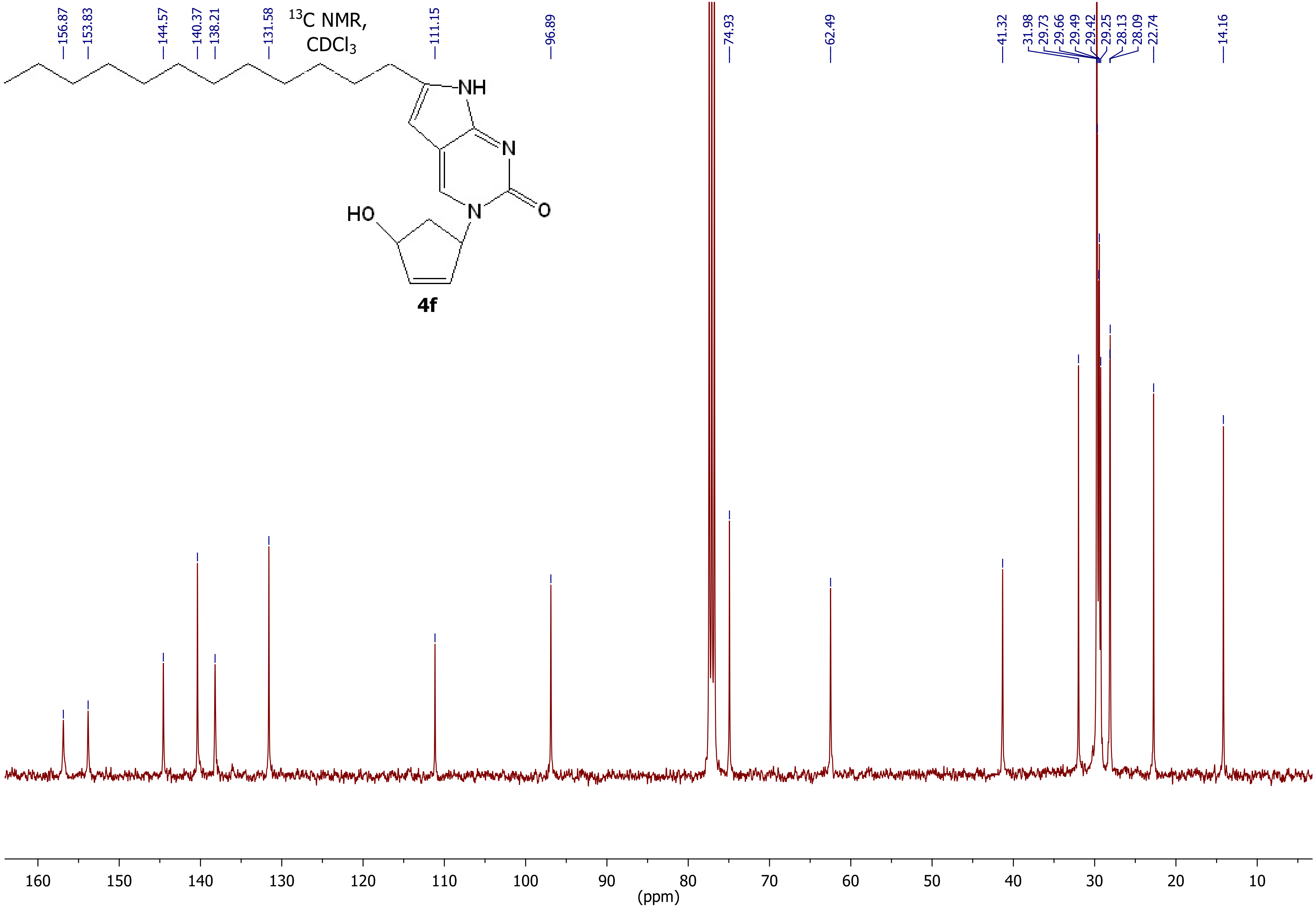

^{13}C NMR, CDCl$_3$
4f
156.87
153.83
144.57
140.37
138.21
131.58
111.15
96.89
74.93
62.49
41.32
31.98
29.73
29.66
29.49
29.42
29.25
28.13
28.09
22.74
14.16
(ppm)

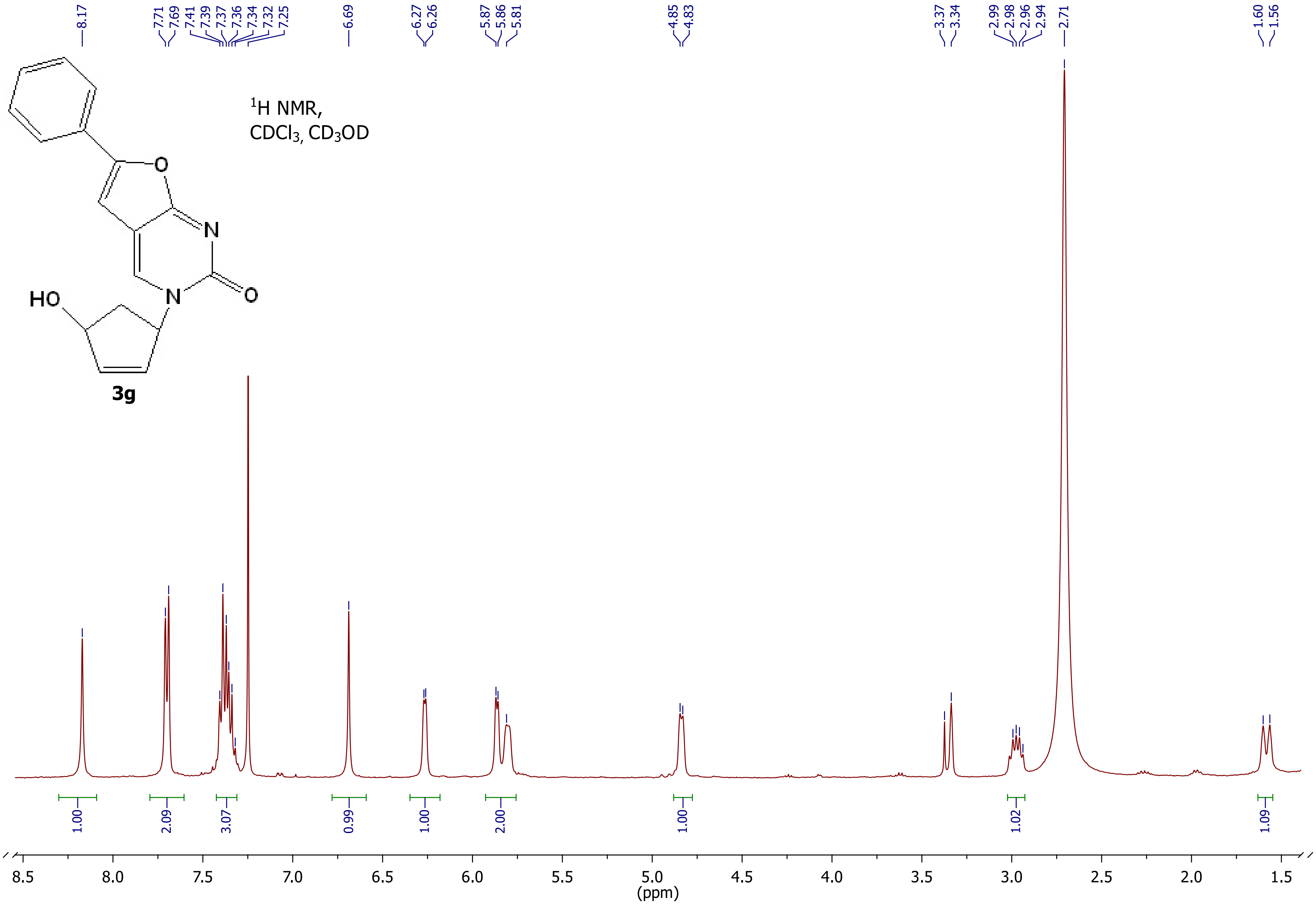

3g
¹H NMR,
CDCl3, CD3OD
8.17
7.71 7.69 7.41 7.39 7.37 7.36 7.34 7.32 7.25
6.69
6.27 6.26
5.87 5.86 5.81
4.85 4.83
3.37 3.34
2.99 2.98 2.96 2.94 2.71
1.60 1.56
1.00 2.09 3.07 0.99 1.00 2.00 1.00 1.02 1.09
8.5 8.0 7.5 7.0 6.5 6.0 5.5 5.0 4.5 4.0 3.5 3.0 2.5 2.0 1.5
(ppm)

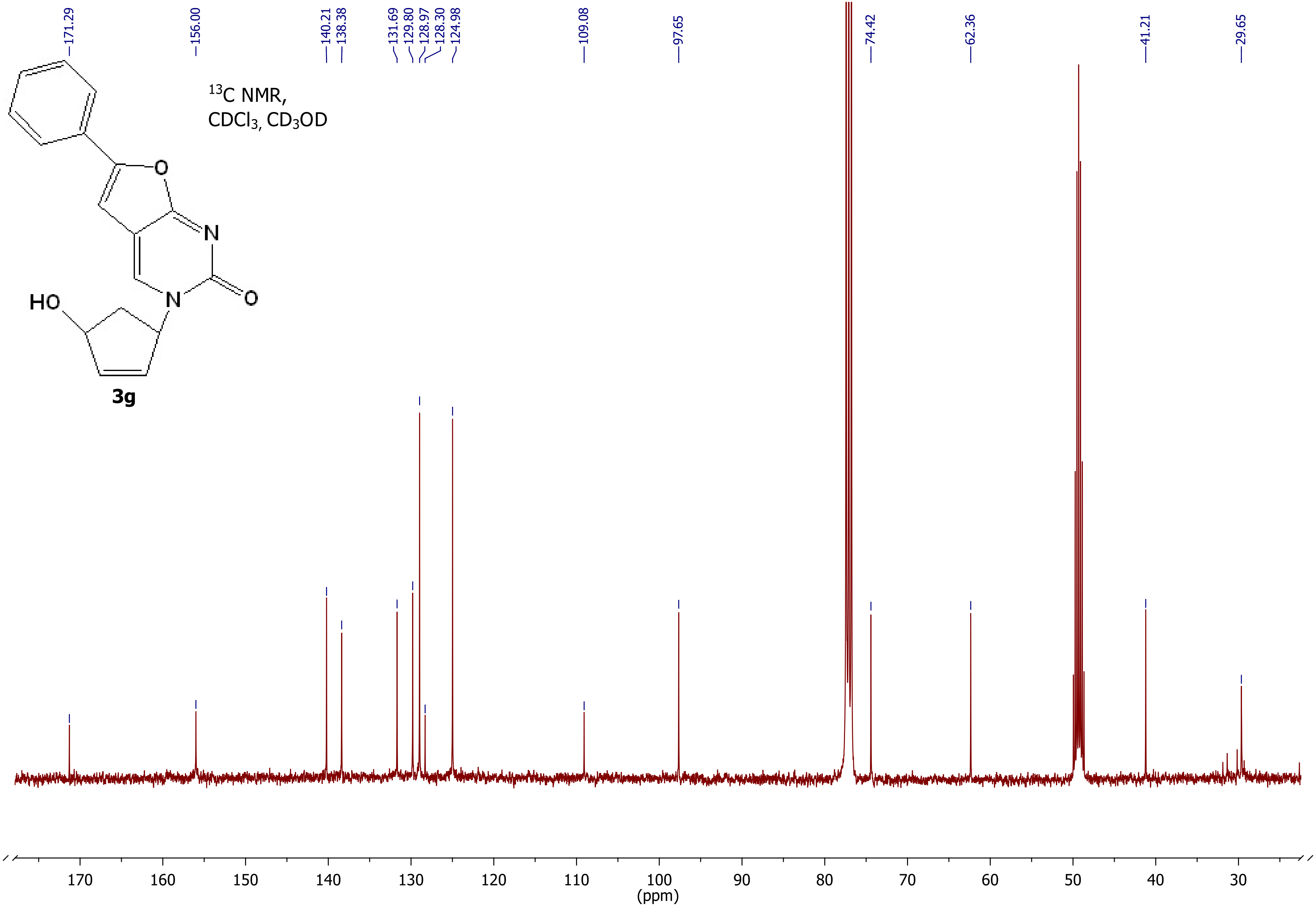

171.29
156.00
140.21
138.38
131.69
129.80
128.97
128.30
124.98
109.08
97.65
74.42
62.36
41.21
29.65
13C NMR,
CDCl3, CD3OD
O
N
O
HO
N
3g
170
160
150
140
130
120
110
100
90
80
70
60
50
40
30
(ppm)

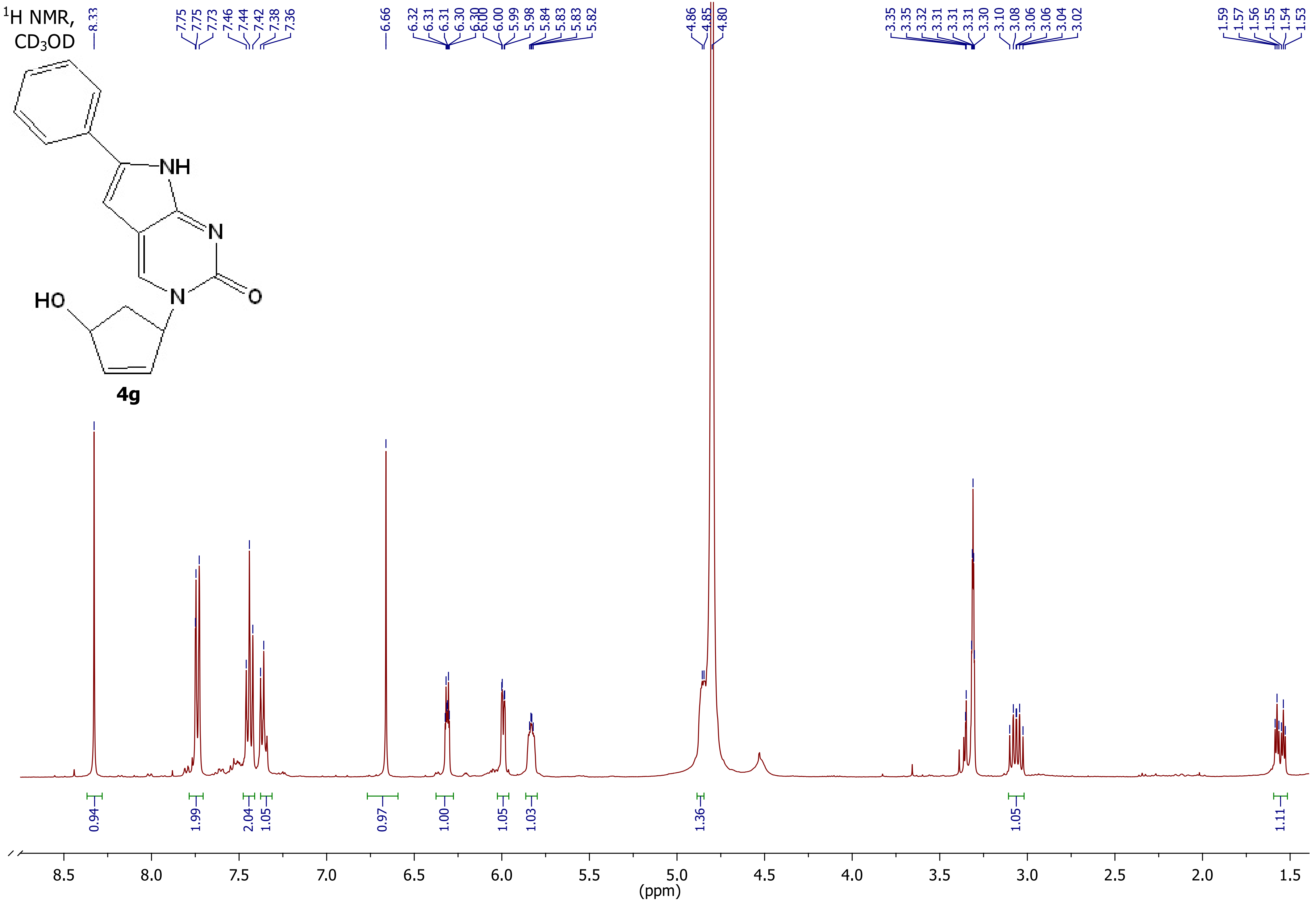

1H NMR, CD3OD
NH
N
N
O
HO
4g
8.33
7.75
7.75
7.73
7.46
7.44
7.42
7.38
7.36
6.66
6.32
6.31
6.31
6.30
6.30
6.00
6.00
5.99
5.98
5.84
5.83
5.83
5.82
4.86
4.85
4.80
3.35
3.35
3.32
3.31
3.31
3.31
3.30
3.10
3.08
3.06
3.06
3.04
3.02
1.59
1.57
1.56
1.55
1.54
1.53
0.94
1.99
2.04
1.05
0.97
1.00
1.05
1.03
1.36
1.05
1.11
8.5
8.0
7.5
7.0
6.5
6.0
5.5
5.0
4.5
4.0
3.5
3.0
2.5
2.0
1.5
(ppm)

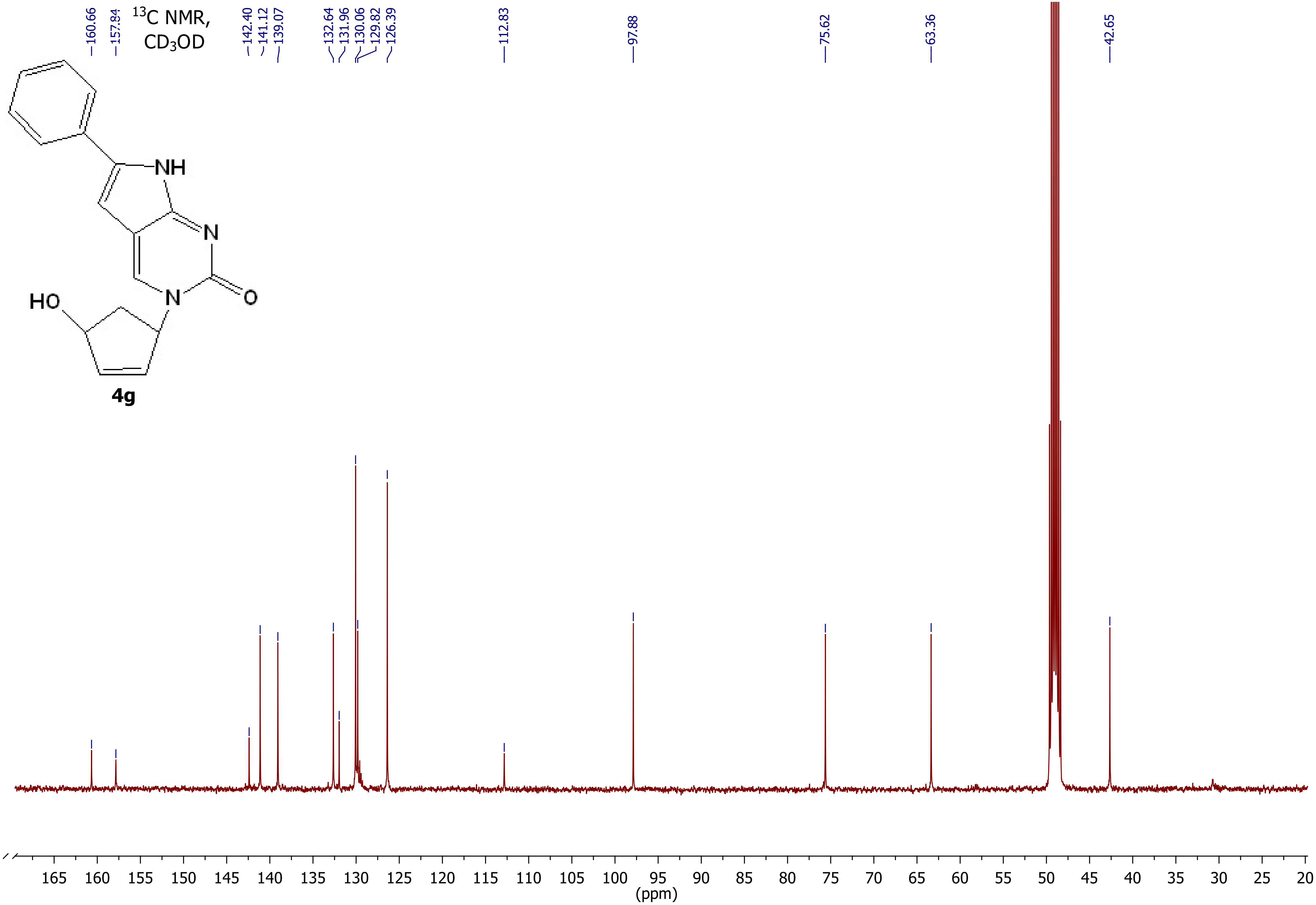

13C NMR,
CD3OD
160.66
157.84
142.40
141.12
139.07
132.64
131.96
130.06
129.82
126.39
112.83
97.88
75.62
63.36
42.65
NH
N
N
O
HO
4g
165 160 155 150 145 140 135 130 125 120 115 110 105 100 95 90 85 80 75 70 65 60 55 50 45 40 35 30 25 20
(ppm)

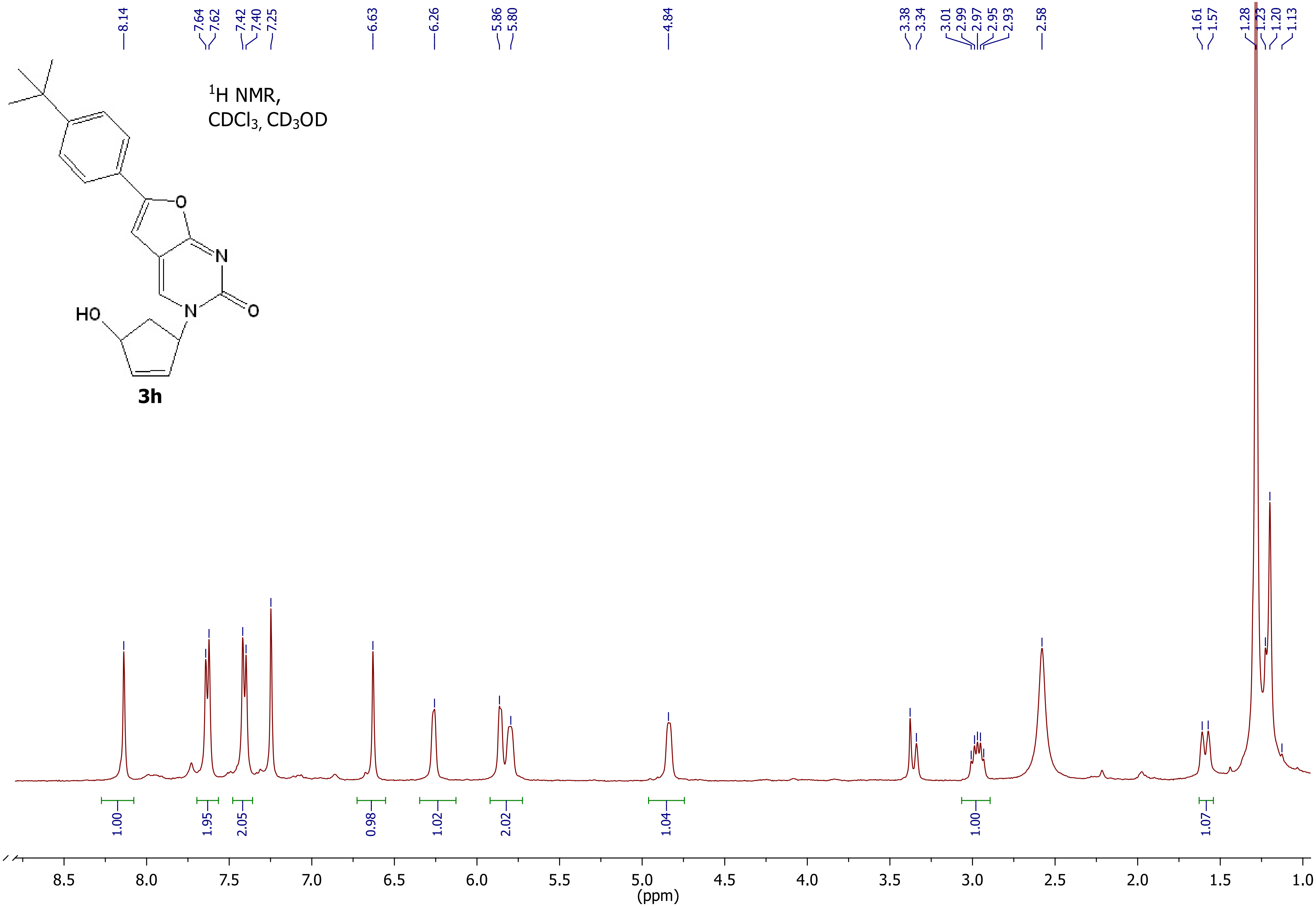

1H NMR,
CDCl3, CD3OD
3h
HO
N
N
O
O
8.14
7.64
7.62
7.42
7.40
7.25
6.63
6.26
5.86
5.80
4.84
3.38
3.34
3.01
2.99
2.97
2.95
2.93
2.58
1.61
1.57
1.28
1.23
1.20
1.13
1.00
1.95
2.05
0.98
1.02
2.02
1.04
1.00
1.07
8.5 8.0 7.5 7.0 6.5 6.0 5.5 5.0 4.5 4.0 3.5 3.0 2.5 2.0 1.5 1.0
(ppm)

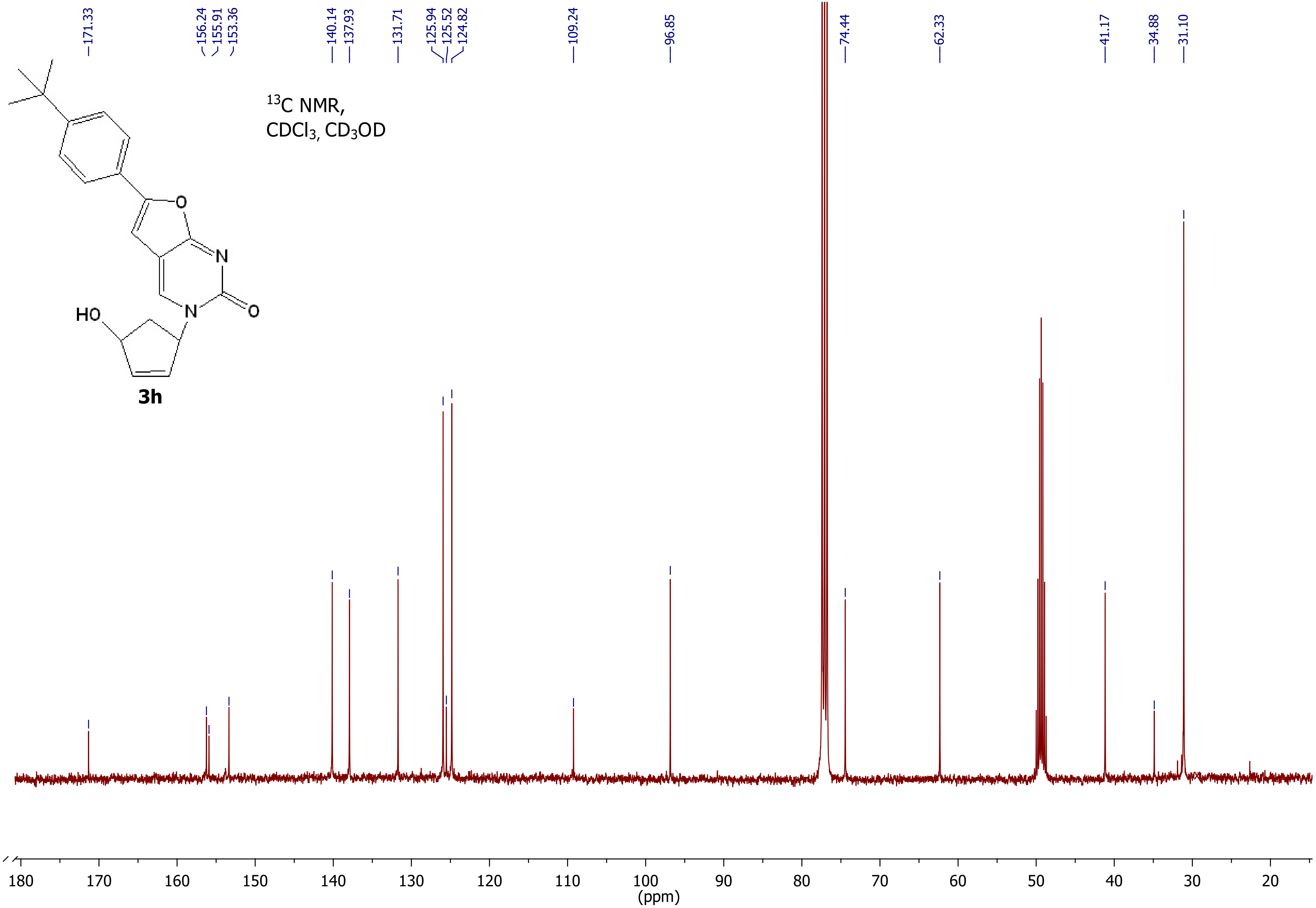

171.33
156.24
155.91
153.36
140.14
137.93
131.71
125.94
125.52
124.82
109.24
96.85
74.44
62.33
41.17
34.88
31.10
^{13}C NMR,
$CDCl_3$, CD_3OD
HO
N
N
O
O
3h
180
170
160
150
140
130
120
110
100
90
80
70
60
50
40
30
20
(ppm)

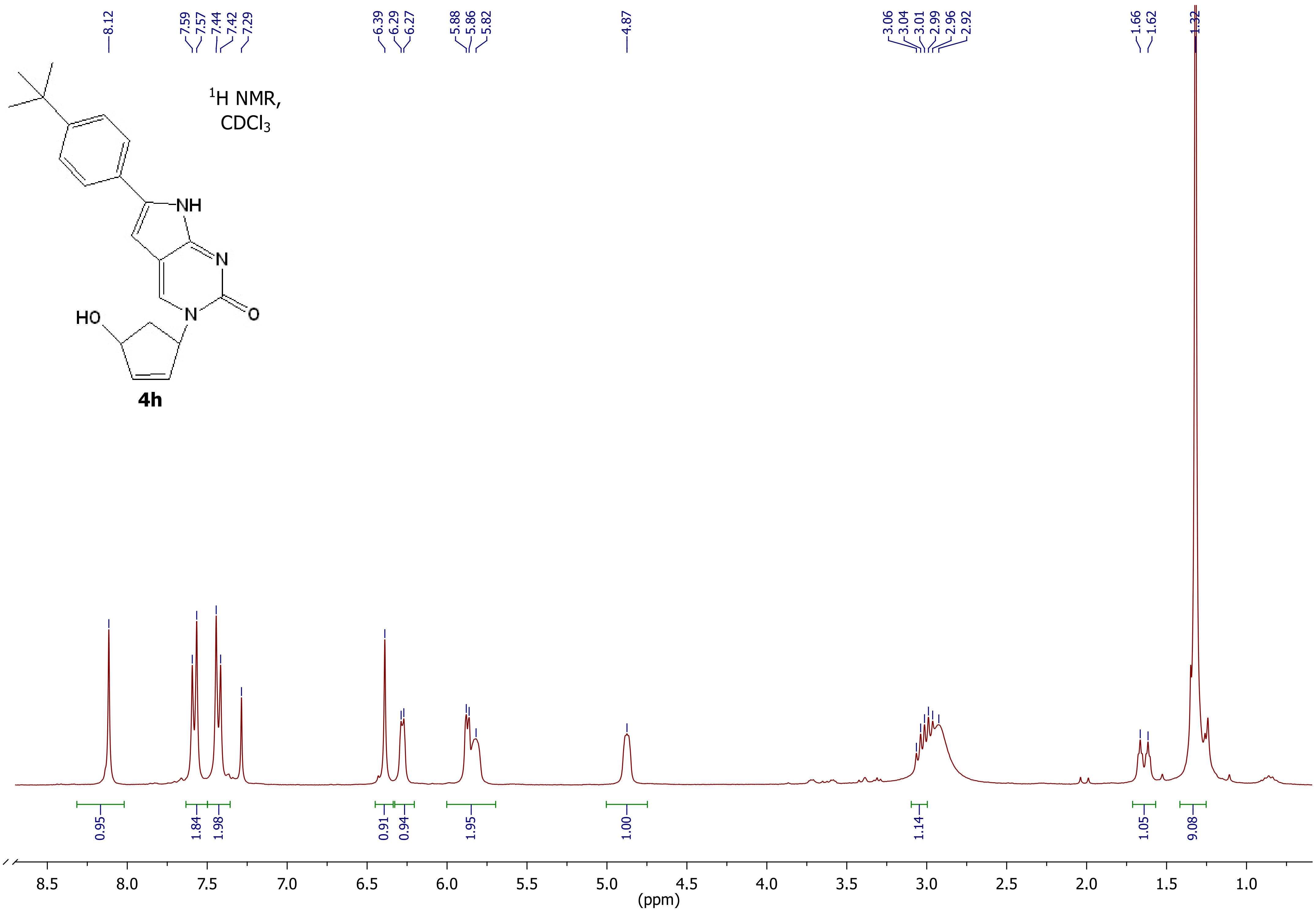
8.12
7.59 7.57 7.44 7.42 7.29
6.39 6.29 6.27
5.88 5.86 5.82
4.87
3.06 3.04 3.01 2.99 2.96 2.92
1.66 1.62
1.32
^{1}H NMR, $CDCl_3$
NH
N
O
N
HO
4h
0.95 1.84 1.98 0.91 0.94 1.95 1.00 1.14 1.05 9.08
8.5 8.0 7.5 7.0 6.5 6.0 5.5 5.0 4.5 4.0 3.5 3.0 2.5 2.0 1.5 1.0
(ppm)

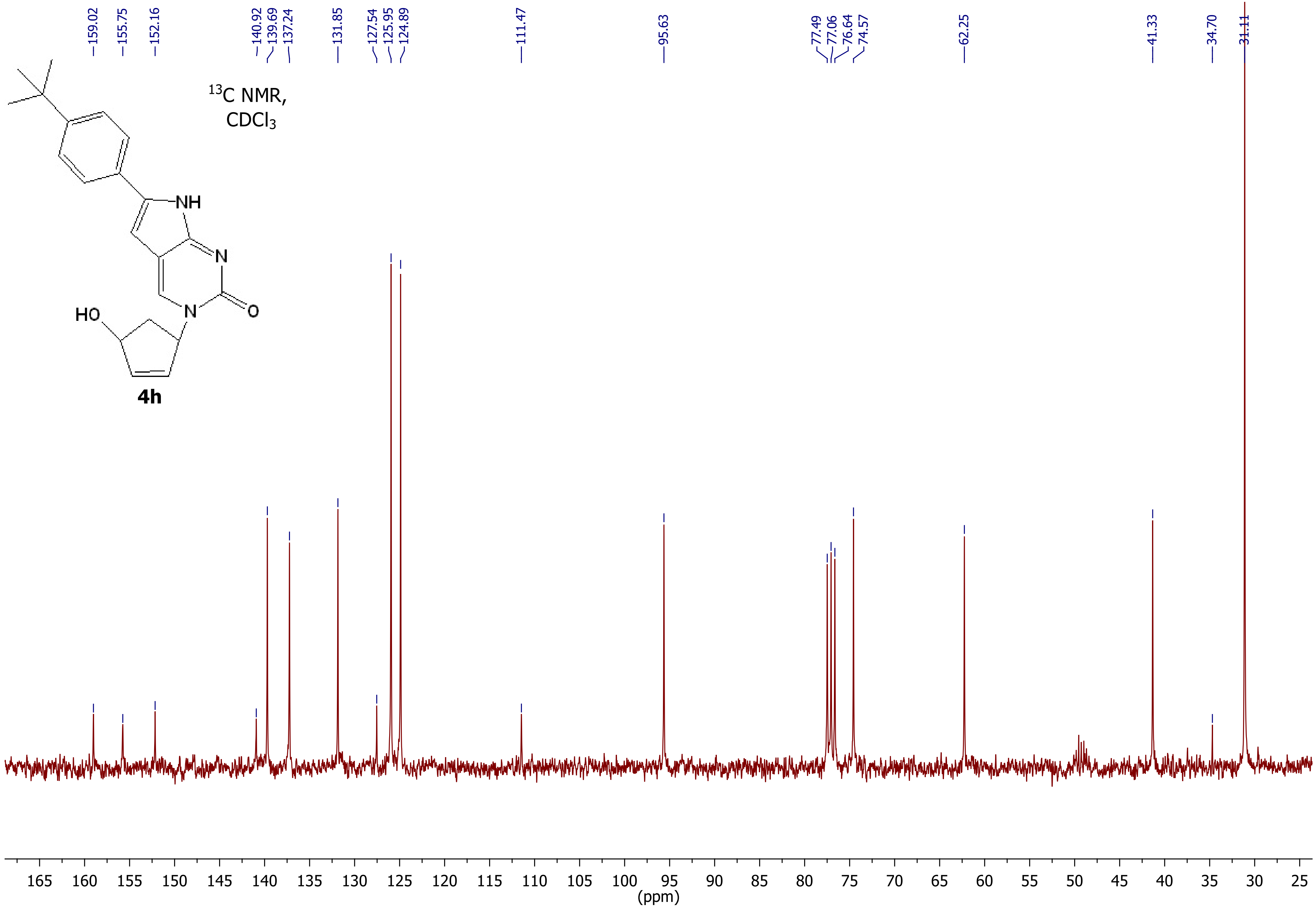
159.02
155.75
152.16
140.92
139.69
137.24
131.85
127.54
125.95
124.89
111.47
95.63
77.49
77.06
76.64
74.57
62.25
41.33
34.70
31.11
^{13}C NMR,
$CDCl_3$
NH
N
HO
N
O
4h
165 160 155 150 145 140 135 130 125 120 115 110 105 100 95 90 85 80 75 70 65 60 55 50 45 40 35 30 25
(ppm)

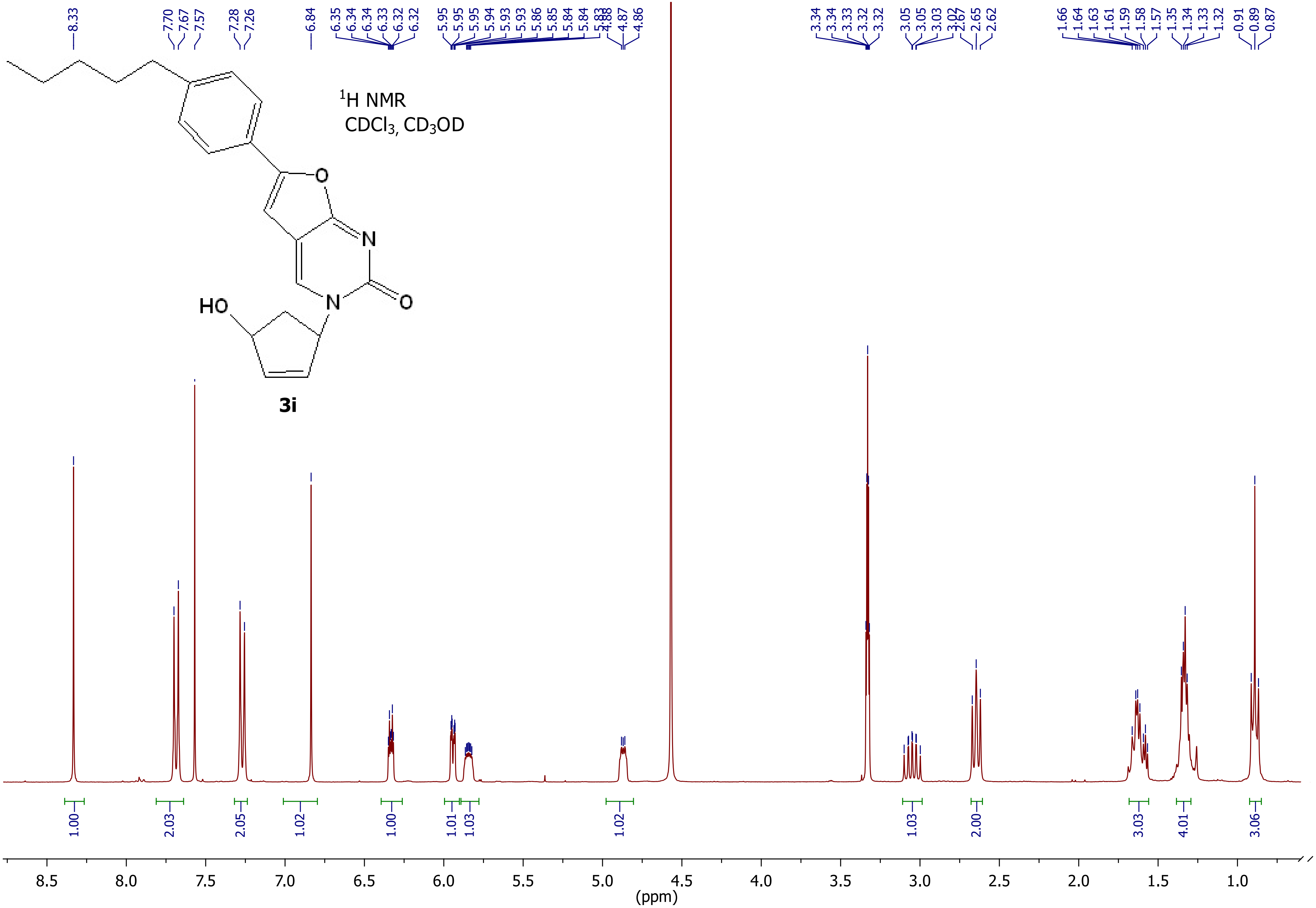

1H NMR
CDCl3, CD3OD
HO
O
N
N
O
3i
(ppm)

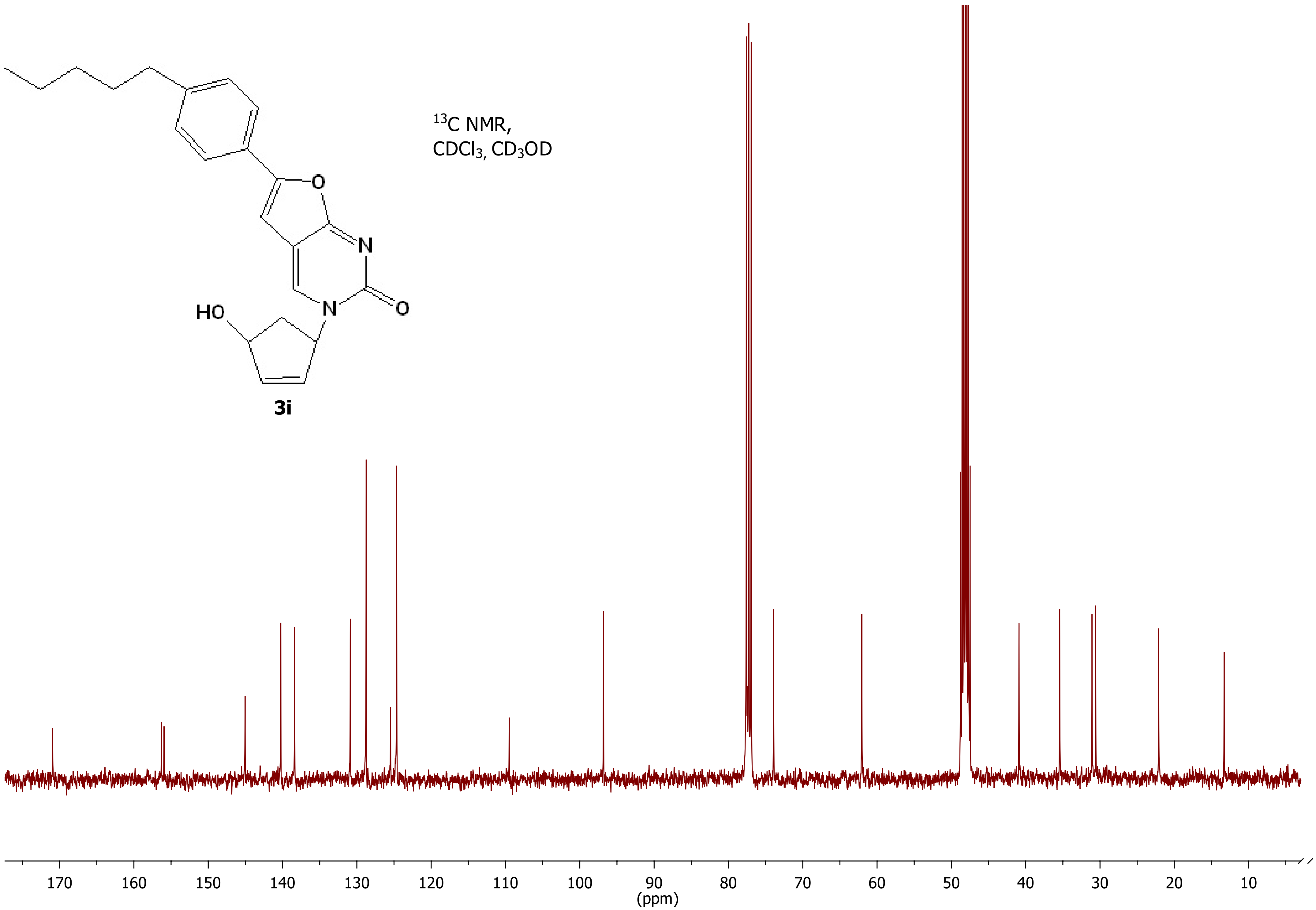

^{13}C NMR,
$CDCl_3$, CD_3OD
HO
O
N
N
O
3i
170 160 150 140 130 120 110 100 90 80 70 60 50 40 30 20 10
(ppm)

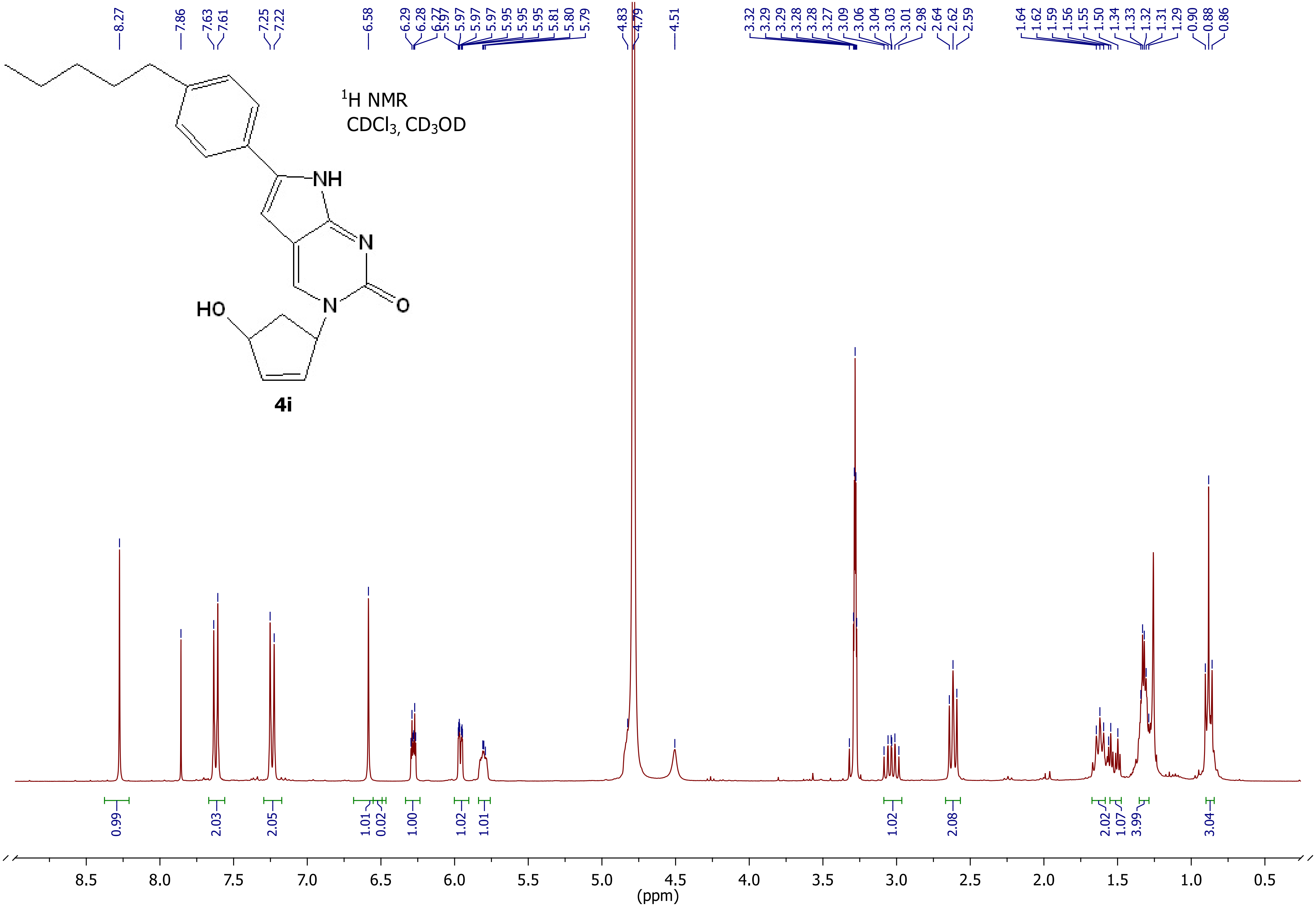

¹H NMR
CDCl3, CD3OD
NH
N
O
N
HO
4i
8.27
7.86
7.63
7.61
7.25
7.22
6.58
6.29
6.28
6.27
5.97
5.97
5.97
5.97
5.95
5.95
5.95
5.81
5.80
5.79
4.83
4.79
4.51
3.32
3.29
3.29
3.28
3.28
3.27
3.09
3.06
3.04
3.03
3.01
2.98
2.64
2.62
2.59
1.64
1.62
1.59
1.56
1.55
1.50
1.34
1.33
1.32
1.31
1.29
0.90
0.88
0.86
0.99
2.03
2.05
1.01
0.02
1.00
1.02
1.01
1.02
2.08
2.02
1.07
3.99
3.04
8.5
8.0
7.5
7.0
6.5
6.0
5.5
5.0
4.5
4.0
3.5
3.0
2.5
2.0
1.5
1.0
0.5
(ppm)

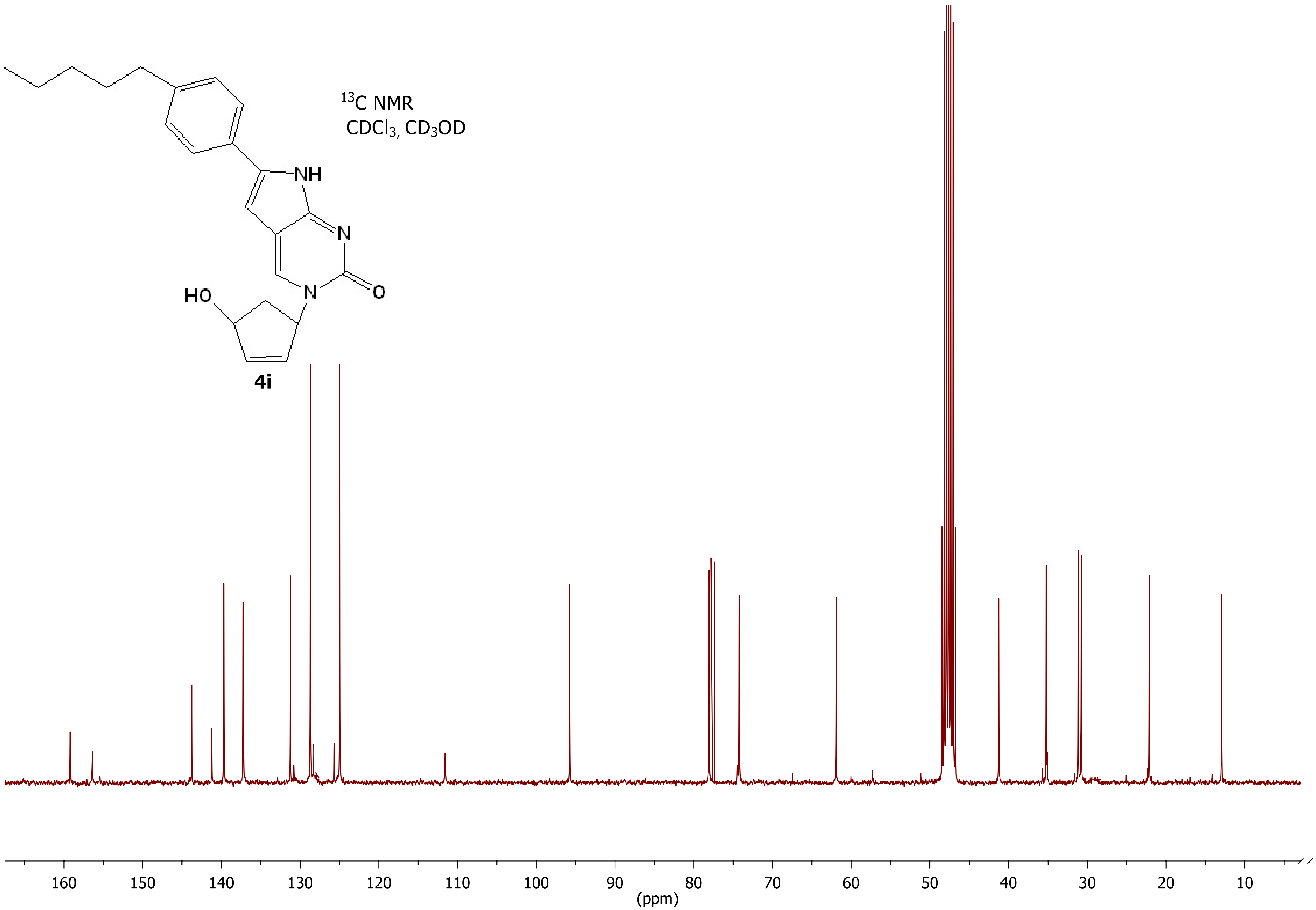
NH
N
O
N
HO
4i
^{13}C NMR
$CDCl_3$, CD_3OD
160 150 140 130 120 110 100 90 80 70 60 50 40 30 20 10
(ppm)

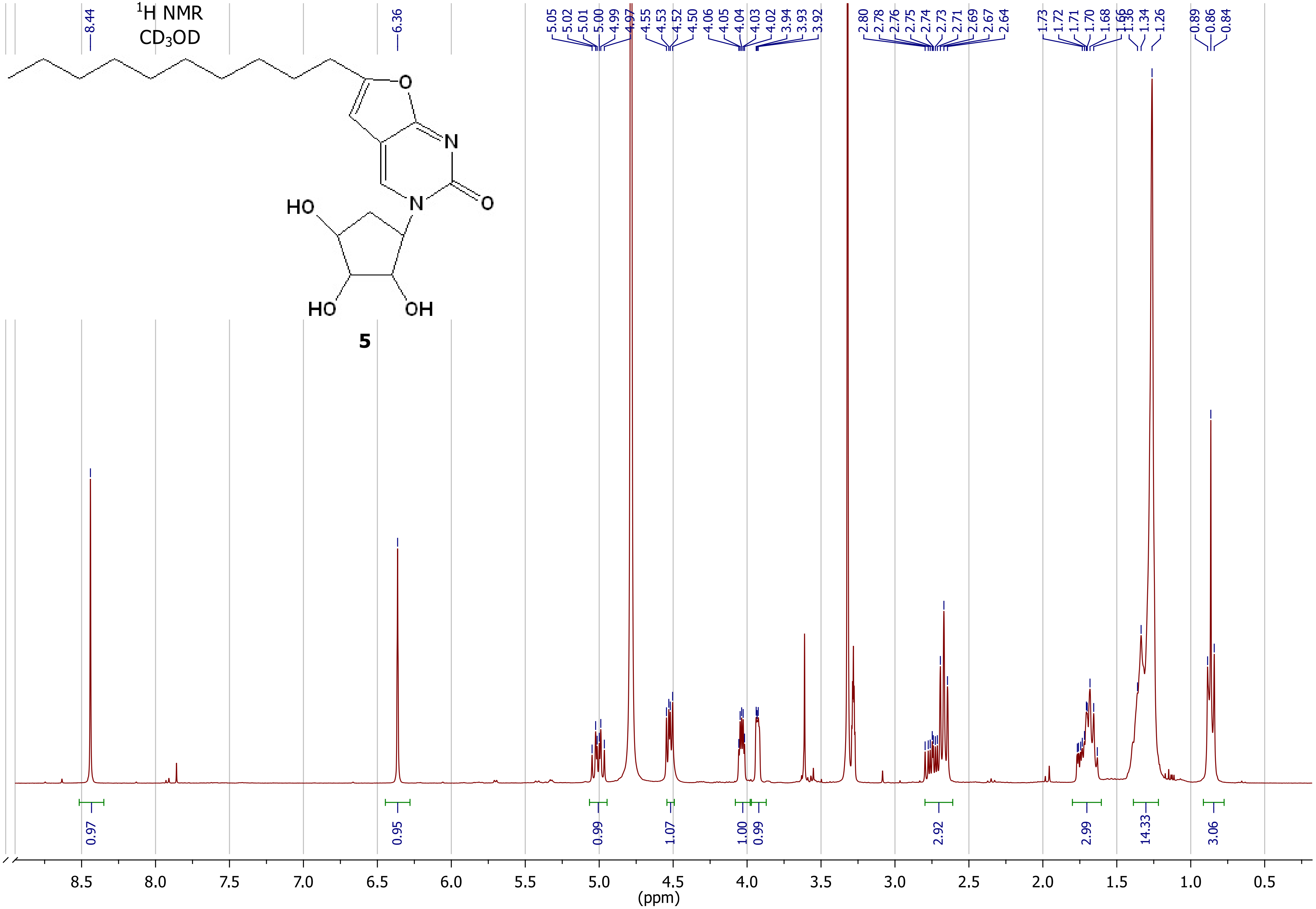
1H NMR
CD3OD
8.44
6.36
5.05 5.02 5.01 5.00 4.99 4.97
4.55 4.53 4.52 4.50
4.06 4.05 4.04 4.03 4.02 3.94 3.93 3.92
2.80 2.78 2.76 2.75 2.74 2.73 2.71 2.69 2.67 2.64
1.73 1.72 1.71 1.70 1.68 1.66 1.36 1.34 1.26
0.89 0.86 0.84
O
N
O
N
HO
HO
OH
5
0.97
0.95
0.99
1.07
1.00
0.99
2.92
2.99
14.33
3.06
8.5 8.0 7.5 7.0 6.5 6.0 5.5 5.0 4.5 4.0 3.5 3.0 2.5 2.0 1.5 1.0 0.5
(ppm)

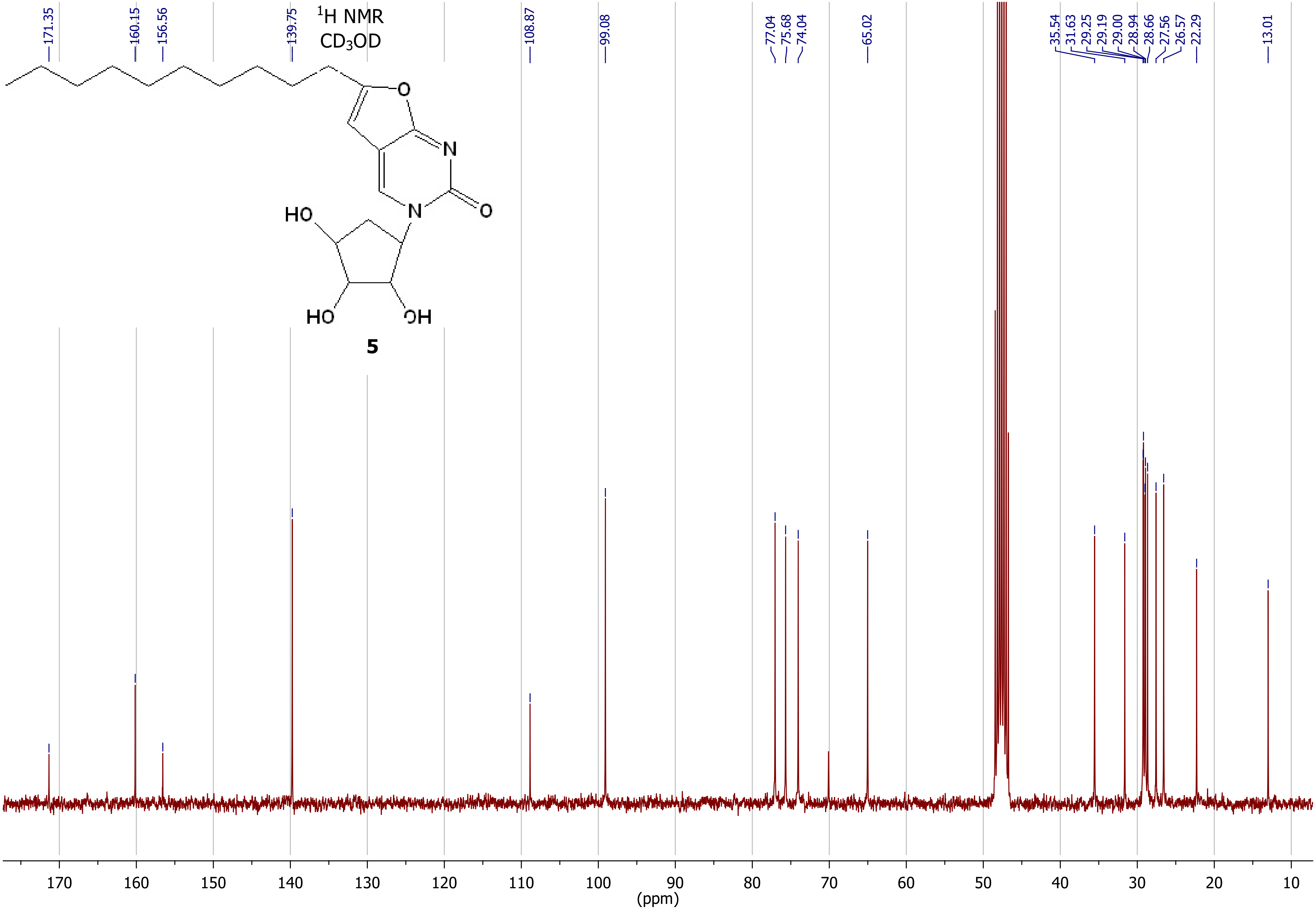

1H NMR
CD3OD
171.35
160.15
156.56
139.75
108.87
99.08
77.04
75.68
74.04
65.02
35.54
31.63
29.25
29.19
29.00
28.94
28.66
27.56
26.57
22.29
13.01
O
N
N
O
HO
HO
OH
5
170 160 150 140 130 120 110 100 90 80 70 60 50 40 30 20 10
(ppm)
